# *Camk2n1* Is a Negative Regulator of Blood Pressure, Left Ventricular Mass, Insulin Sensitivity, and Promotes Adiposity

**DOI:** 10.1161/HYPERTENSIONAHA.118.12409

**Published:** 2019-07-22

**Authors:** Neza Alfazema, Marjorie Barrier, Sophie Marion de Procé, Robert I. Menzies, Roderick Carter, Kevin Stewart, Ana Garcia Diaz, Ben Moyon, Zoe Webster, Christopher O.C. Bellamy, Mark J. Arends, Roland H. Stimson, Nicholas M. Morton, Timothy J. Aitman, Philip M. Coan

**Affiliations:** 1 From the MRC Institute of Genetics and Molecular Medicine, Edinburgh, United Kingdom (N.A., M.B., S.M.d.P., T.J.A., P.M.C.); 2 Centre for Cardiovascular Science, Queen’s Medical Research Institute, University of Edinburgh, United Kingdom (R.I.M., R.C., K.S., R.H.S., N.M.M.); 3 MRC London Institute of Medical Sciences, Imperial College London, United Kingdom (A.G.D., B.M., Z.W.); 4 Division of Pathology, Centre for Comparative Pathology, Edinburgh CRUK Cancer Centre, United Kingdom (C.O.C.B., M.J.A.).

**Keywords:** adiposity, blood pressure, hypertrophy, metabolic syndrome, rats

## Abstract

Metabolic syndrome is a cause of coronary artery disease and type 2 diabetes mellitus. *Camk2n1* resides in genomic loci for blood pressure, left ventricle mass, and type 2 diabetes mellitus, and in the spontaneously hypertensive rat model of metabolic syndrome, *Camk2n1* expression is cis-regulated in left ventricle and fat and positively correlates with adiposity. Therefore, we knocked out *Camk2n1* in spontaneously hypertensive rat to investigate its role in metabolic syndrome. Compared with spontaneously hypertensive rat, *Camk2n1*^−/−^ rats had reduced cardiorenal CaMKII (Ca^2+^/calmodulin-dependent kinase II) activity, lower blood pressure, enhanced nitric oxide bioavailability, and reduced left ventricle mass associated with altered hypertrophic networks. Camk2n1 deficiency reduced insulin resistance, visceral fat, and adipogenic capacity through the altered cell cycle and complement pathways, independent of CaMKII. In human visceral fat, *CAMK2N1* expression correlated with adiposity and genomic variants that increase *CAMK2N1* expression associated with increased risk of coronary artery disease and type 2 diabetes mellitus. Camk2n1 regulates multiple networks that control metabolic syndrome traits and merits further investigation as a therapeutic target in humans.

## Abstract 2

Supplemental Digital Content is available in the text.

**See Editorial, pp 495–496**

Metabolic syndrome (MetS) affects one in 4 people and is a major cause of coronary artery disease and type 2 diabetes mellitus (T2DM).^1^ MetS genetic determinants have been successfully elucidated in the spontaneously hypertensive rat (SHR), an established model of human polygenic MetS.^2^ Our previous studies identified *Camk2n1* as a cis-regulated expression quantitative trait locus (cis-eQTL) in left ventricle (LV) and epididymal adipose tissue (EAT),^3^ and as a quantitative trait transcript that significantly positively correlates with relative fat pad weight (*r*^2^=0.67, *P*_(adj)_=0.0002) and adipocyte volume (*r*^2^=0.69, *P*_(adj)_=0.0002).^4^ Furthermore, *Camk2n1* is close to the peak logarithm of the odds (to the base 10) score in QTLs for systolic blood pressure(BP; Bp292, Bp180, 433, and 441 Kb from peak) and relative LV weight (Cm24 and 441 Kb from peak) and resides in a QTL for T2DM (Niddm30).^5^

Camk2n1 has been reported through in vitro and in vivo peptide inhibition studies to be a specific inhibitor of CaMKII (Ca^2+^/calmodulin-dependent kinase II),^6^ an enzyme activated by Ca^2+^/calmodulin binding, which regulates multiple signaling pathways that control vascular tone,^7^ in vitro adipogenesis,^8^ and insulin-stimulated glucose uptake.^9^ However, the in vivo functions of endogenous Camk2n1 in cardiometabolic disease have not been studied directly, whereas experimental inhibition of CaMKII has been extensively studied because of the strong association between CaMKII hyperactivity, LV hypertrophy, and heart failure in humans.^10^ CaMKII inhibition has been induced experimentally using synthetic and transgenic CaMKII inhibitors. These studies have shown that such inhibitors can protect from Ang II (angiotensin II)–induced hypertension^7^ and LV hypertrophy.^11^ However, these CaMKII inhibitors have known off-target effects independent of CaMKII and may not mimic fully endogenous Camk2n1 functions.^12^

In other studies, knockout of CaMKII has been shown to protect from pressure-overload LV dysfunction but not prevent LV hypertrophy.^13^ CaMKII knockout improved hepatic insulin signaling in obese mice, while enhanced CaMKII activation has been shown to induce hyperinsulinemia and glucose intolerance.^14^ Taken together, these data suggest a potential causal role for Camk2n1 in cardiometabolic disease.

Here, we test the hypothesis that *Camk2n1* regulates cardiometabolic traits by generating a *Camk2n1* knockout in the SHR model of MetS.

## Methods

The authors declare that all supporting data are available within the article and detailed methods and supplementary results in the online-only Data Supplement.

### Rats

SHR-*Camk2n1*^−/−^ knockout rats (referred to hereafter as *Camk2n1*^−/−^ rats) were generated on an SHR/NCrl background (Charles River, Margate, United Kingdom), containing a 38bp deletion in exon 1 of *Camk2n1* confirmed by whole genome sequencing, polymerase chain reaction, and Immunoblot (Figure S1A through S1C in the online-only Data Supplement). All procedures were performed in accordance with UK Home Office regulations.

### Human Participants

Visceral adipose tissue was obtained intraoperatively following ethical approval from 28 lean, obese, and obese type 2 diabetic subjects who were attending the Royal Infirmary of Edinburgh for elective abdominal surgery for nonmalignant disease (Lothian NRS Human Annotated Bioresource [15/ES/0094]).

### Statistical Analysis

Unpaired *t* tests, 1-way, or 2-way ANOVA followed by Fisher least significant difference post hoc test were used to assess differences between SHR, *Camk2n1*^−/−^ and treatment, and differences between lean, obese, and obese diabetic subjects. All statistics were performed using Minitab Express (v1.5.1).

## Results

### *Camk2n1* Knockout Rat

We generated a *Camk2n1*^−/−^ rat using zinc finger nuclease that created a 38 bp deletion in exon 1 of *Camk2n1* confirmed by a polymerase chain reaction, Sanger sequencing, and whole genome sequencing (Figure S1A). Truncation of the transcript and absence of Camk2n1 protein were confirmed by a polymerase chain reaction and immunoblot, respectively (Figure S1B and S1C).

### Blood Pressure

**Figure 1.**
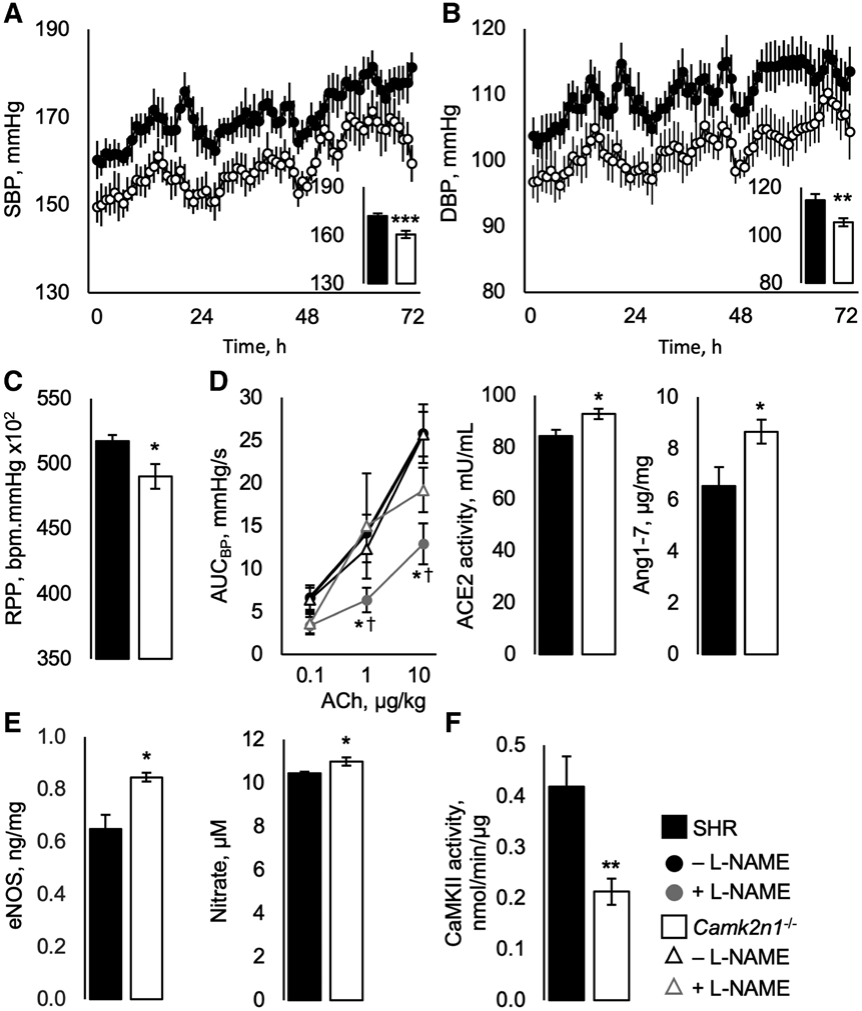
Cardiovascular physiology and in vivo vasoreactivity in spontaneously hypertensive rat (SHR) and *Camk2n1*^-/-^ rats. **A**, Systolic (SBP) and (**B**) diastolic blood pressures (DBP) and (**C**) rate pressure product (RPP). **D**, In vivo physiological analysis of ACE2 (angiotensin II–converting enzyme)-Ang-(1–7)-Mas signaling, area under the BP curve (AUC_BP_) against acetylcholine (ACh) in the presence or absence of Nω-Nitro-L-arginine methyl ester hydrochloride (L-NAME) with renal angiotensin II–converting enzyme (ACE2) activity and angiotensin-(1–7) concentrations. **E**, Renal eNOS (endothelial nitric oxide synthase) and serum nitrate concentrations. **F**, Renal CaMKII activity. Mean±SEM, n=13/group for telemetry, n=7–9/group for renal and serum measurements. Significant differences by genotype (SHR and *Camk2n1*^−/−^) (*P<0.05, **P<0.005, and ***P<0.001) and treatment (†*P*<0.05).

To determine the cardiovascular consequences of *Camk2n1* deletion, we measured BP in SHR and *Camk2n1*^−/−^ rats. Mean systolic BP (−Δ12 mm Hg, *P*<0.001) and diastolic BP (−Δ10 mm Hg, *P*<0.005) BPs were significantly lower in *Camk2n1*^−/−^ than SHR, and although heart rate was similar (SHR=299±3 *Camk2n1*^−/−^=305±6, *P*>0.05), rate pressure product was reduced significantly by 5% (Figure 1A through 1C).

To test whether lower BP in *Camk2n1*^−/−^ rats was associated with altered vasodilatory mechanisms, we tested in vivo vasoreactivity to acetylcholine in the presence and absence of Nω-Nitro-L-arginine methyl ester hydrochloride or Ang(1–7) antagonist A-779 and analyzed the ACE2 (angiotensin II–converting enzyme)-Ang-(1–7)-Mas pathway. Area under the blood pressure curve responses to the lowest level of acetylcholine infusion were similar in SHR and *Camk2n1*^−/−^ (Figure 1D). At higher doses of acetylcholine, *Camk2n1*^−/−^ had a similar response in the presence or absence of Nω-Nitro-L-arginine methyl ester hydrochloride, whereas responses in SHR treated with Nω-Nitro-L-arginine methyl ester hydrochloride were reduced (Figure 1D). These changes were associated with increased renal ACE2 activity and Ang (1–7) concentrations in *Camk2n1*^−/−^ kidney and serum (Figure 1D; Table). In addition, renal and serum eNOS (endothelial nitric oxide synthase) and serum nitrate levels were elevated in *Camk2n1*^−/−^ compared with SHR (Figure 1E; Table). Conversely, CaMKII activity was reduced by 50% (Figure 1F). A-779 did not affect BP modulation (*P*>0.05, Figure S2E).

**Table.**
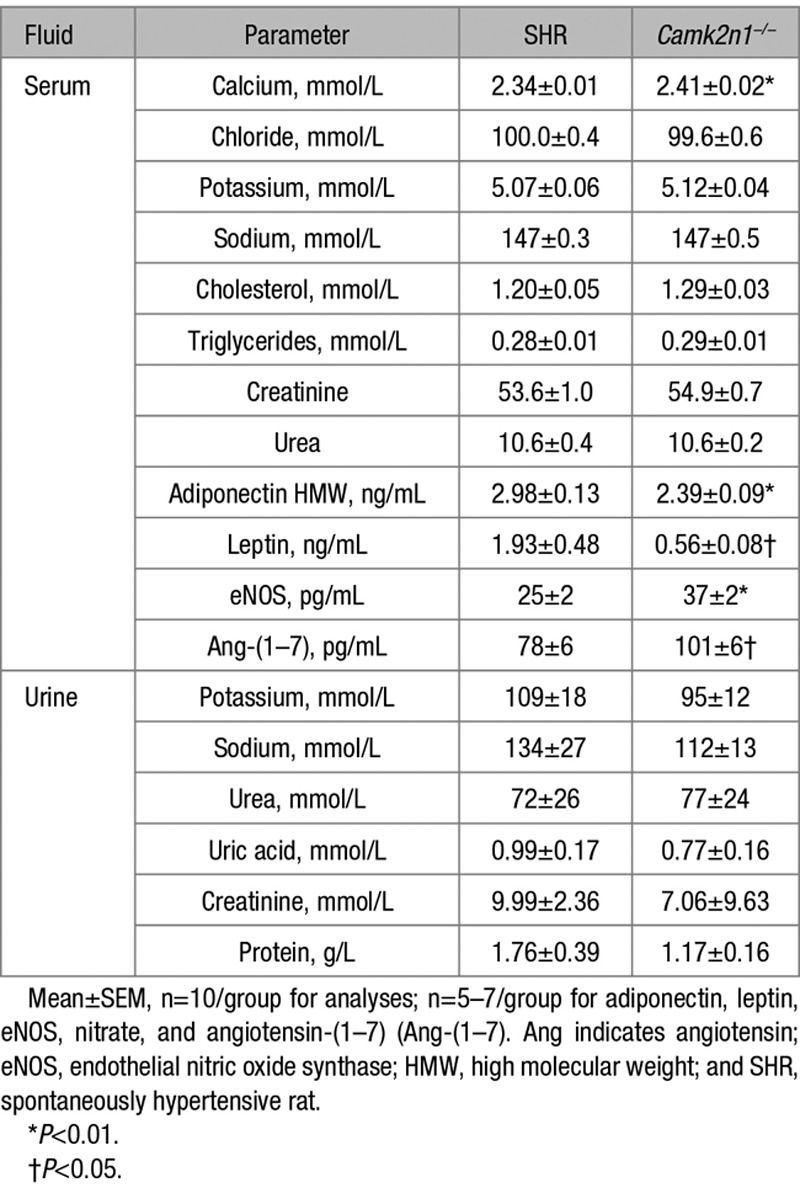
Serum and Urinary Biochemical Analyses

In *Camk2n1*^−/−^ rats, kidney wet mass was reduced by 4%, but no differences in hypertension-related vascular damage or kidney function markers were observed (Figure S2A and S2B; Table).

### LV Mass

To determine the effects of *Camk2n1* knockout on LVH, we investigated LV structure and function at baseline and with isoproterenol-induced (CaMKII-associated) hypertrophy in SHR and *Camk2n1*^−/−^ rats.

**Figure 2.**
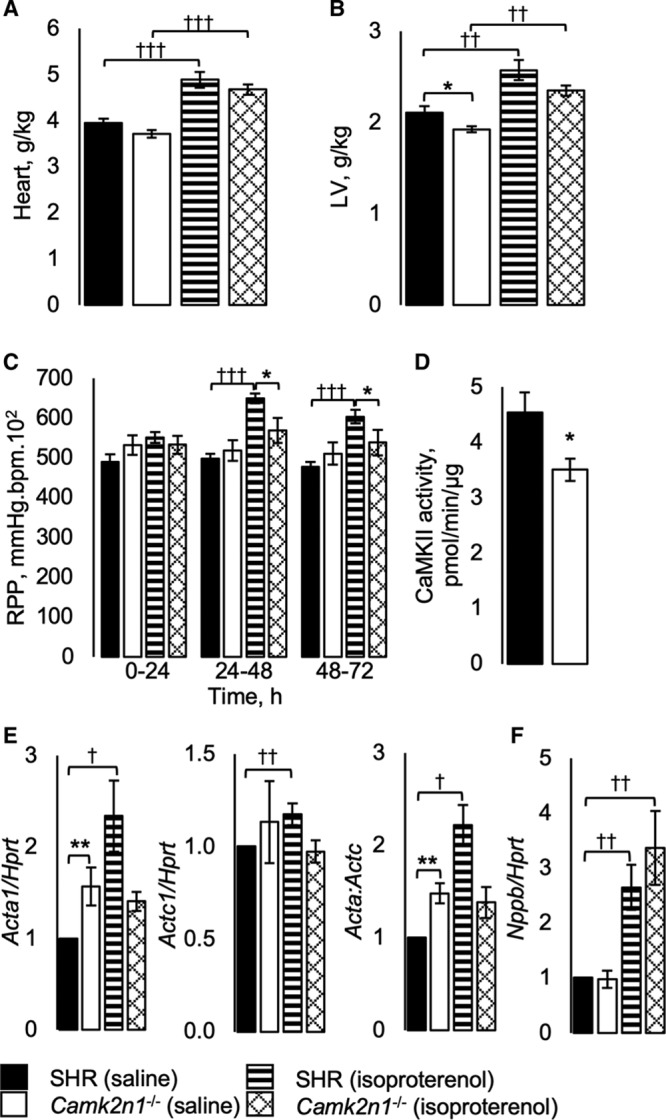
Cardiac phenotype in spontaneously hypertensive rat (SHR) and *Camk2n1*^−/−^ rats. **A**, Heart and (**B**) left ventricle (LV) wet masses. **C**, Rate pressure product (RPP). **D**, LV CaMKII (Ca^2+^/calmodulin-dependent kinase II) activity. Average transcript levels normalized to hypoxanthine-guanine phosphoribosyltransferase, *Hprt* for (**E**) α-skeletal actin, *Acta*, α-cardiac actin, *Actc*, the *Acta*:*Actc* ratio and (**F**) natriuretic protein b, *Nppb*. Mean±SEM n=5–7/group. Significant differences by genotype (SHR and *Camk2n1*^−/−^) (*P<0.05, **P<0.01, and ***P<0.001) and treatment (†*P*<0.05, ††*P*<0.01, and †††*P*<0.001).

LV mass at baseline was reduced by 9% in *Camk2n1*^−/−^ compared with SHR, whereas baseline heart mass was similar (Figure 2A and 2B). Histological signs of inflammation and fibrosis, blood vessel and cardiomyocyte density and morphology in LV were similar for both genotypes (Figure S2C and S2D).

To test whether *Camk2n1* deficiency would protect against isoproterenol-stimulated LVH, we performed a 72 hours isoproterenol hypertrophic challenge. Isoproterenol treatment caused similar increases in heart mass and rate and reductions in BP in SHR and *Camk2n1*^−/−^ (Figure 2A and 2B; Figure S3A through S3C). However, rate pressure product increased in SHR only (Figure 2C).

CaMKII activity and hypertrophy-related transcripts *Acta*, *Actc*, and *Nppb* were assessed in LV. CaMKII activity was reduced by 23% in *Camk2n1*^−/−^ compared with SHR LV (Figure 2D). *Acta1* transcripts were 1.5-fold greater in saline-treated *Camk2n1*^−/−^ than in SHR, and after treatment with isoproterenol, *Acta* and *Actc* increased in SHR LV only, thereby increasing the ratio of *Acta1*:*Actc1* in SHR compared with *Camk2n1*^−/−^ LV (Figure 2E). Camk2n1 deletion did not affect *Nppb* expression (Figure 2F).

### Insulin Sensitivity

**Figure 3.**
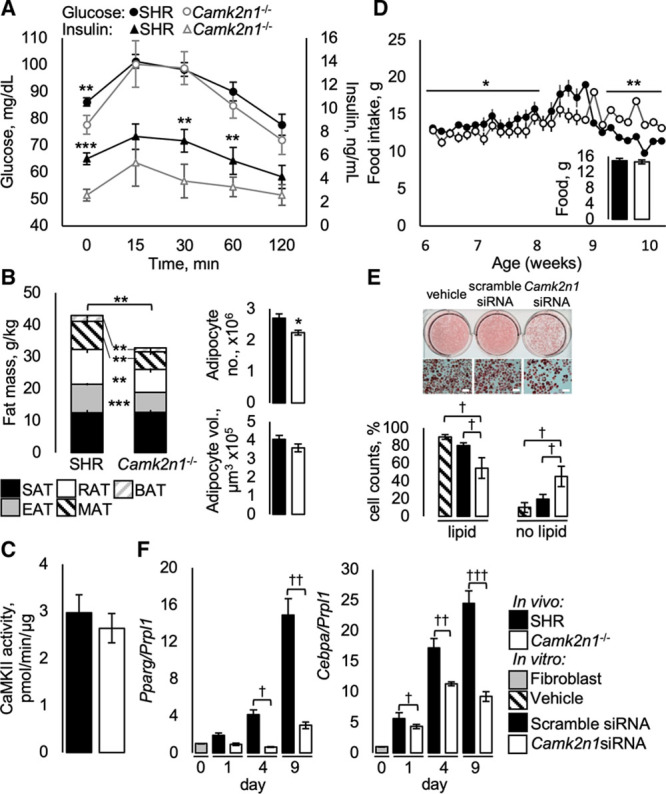
Metabolic phenotype in spontaneously hypertensive rat (SHR) and *Camk2n1*^−/−^ rats. **A**, Blood glucose and insulin curves during oral glucose tolerance testing. **B**, Relative adipose tissue wet masses for subcutaneous (SAT), epididymal (EAT), retroperitoneal fat (RAT), mesenteric fat (MAT), and brown adipose tissues (BAT), EAT adipocyte volume and number. **C**, EAT CaMKII (Ca^2+^/calmodulin-dependent kinase II) activity. **E**, Representative wells and light micrograph images (scale bar 200 µm) of 3T3-L1 adipocytes and percentage of cells with or without lipid accumulation. **F**, *Cebpa* and *Pparg* transcript levels 0, 1, 4, and 9 d after knockdown. In vivo: mean±SEM n=7/group, significant differences (*P<0.05, **P<0.01, and ***P<0.001). In vitro mean±SEM n=3 independent experiments, significant differences (†*P*<0.05, ††*P*<0.01, and †††*P*<0.001).

To establish a function for Camk2n1 in glucose metabolism, we measured the effectiveness of endogenous insulin to stimulate peripheral tissues glucose uptake, after an oral glucose bolus. Fasting plasma glucose concentrations in *Camk2n1*^−/−^ were significantly lower than SHR (*P*<0.01; Figure 3A), although, area under the glucose curve was similar (*P*>0.05). Plasma insulin concentrations at t_0_ and t_30_–t_60_ minutes and area under the insulin curve following glucose bolus were significantly reduced together with homeostatic model assessment of insulin resistance (SHR, 1.22±0.06, *Camk2n1*^−/−^, 0.51±0.06, *P*=0.00001) in *Camk2n1*^−/−^ compared with SHR (Figure 3A).

### Adiposity

**Figure 4.**
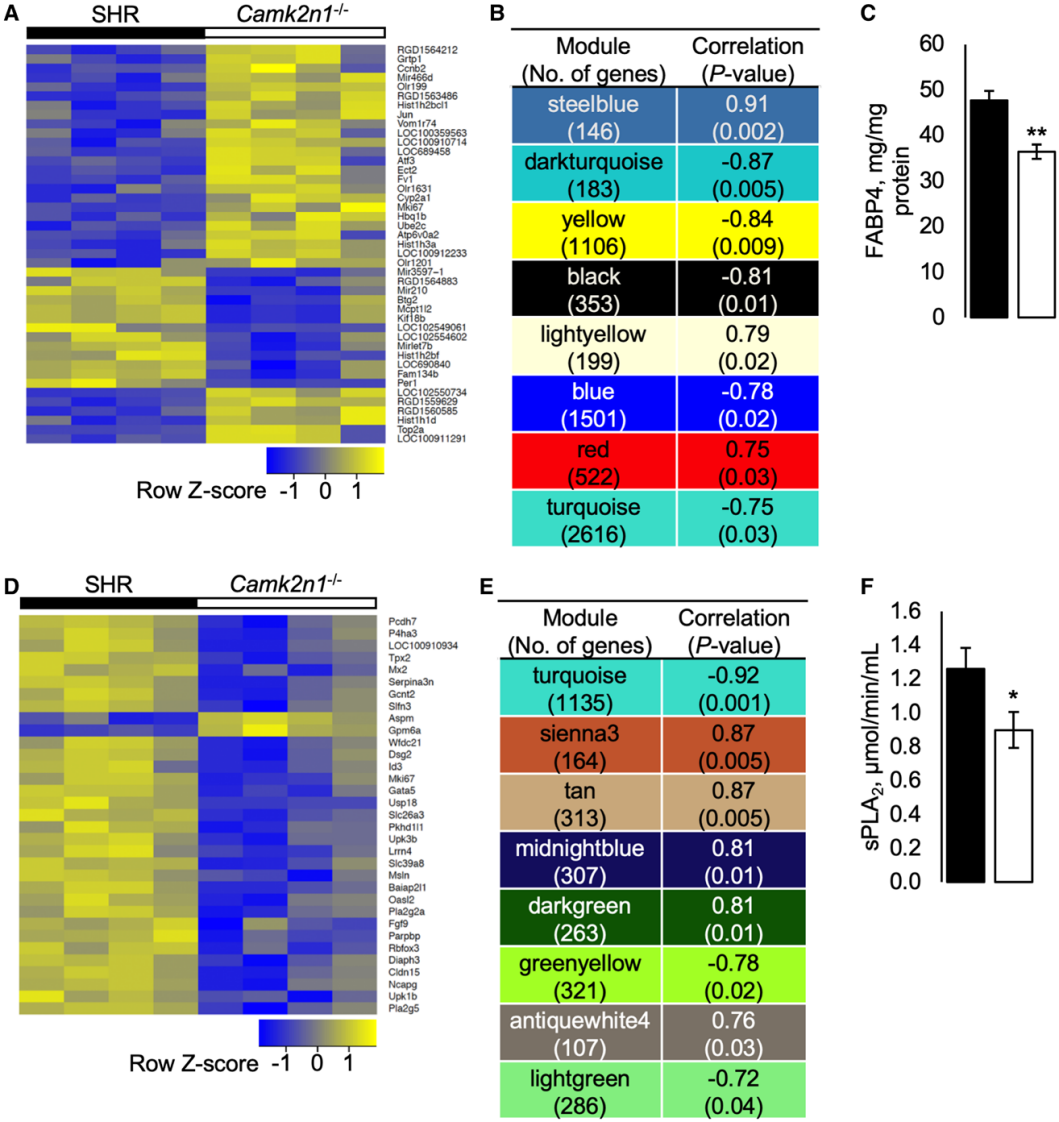
Weighted coexpression network analysis (WGCNA) of spontaneously hypertensive rat (SHR) and *Camk2n1*^−/−^ left ventricle (LV) and epididymal adipose tissue (EAT) transcriptomes. **A**, Heat map of significantly differentially expressed genes (>2-fold) (DEG) in LV. **B**, Significantly enriched modules in LV correlated with *Camk2n1*. **C**, LV FABP4 (fatty acid binding protein 4) concentrations. **D**, Heat map of DEG (>2-fold) in EAT. **E**, Significantly enriched modules in EAT correlated with *Camk2n1*. **F**, Serum soluble phospholipase A2 (sPLA_2_) activity. Transcriptomics n=4 rat/tissue; in vivo validation n=9. Mean±SEM, significant differences (*P<0.05 and **P<0.01).

To assess a causal role for *Camk2n1* in adiposity, we assessed adipose tissue mass, morphology, and adipocyte function of SHR and *Camk2n1*^−/−^ fat pads. Relative masses of visceral (EAT, mesenteric, and retroperitoneal adipose tissue) and brown adipose tissue were significantly reduced in *Camk2n1*^−/−^ compared with SHR, despite similar growth rate and total body mass (*P*>0.05; FigureS4A) with an overall 23% reduction (*P*=0.004) in total relative fat mass (Figure 3B). Subcutaneous fat mass was unaltered by *Camk2n1* deletion (Figure 3B). Morphometric assessment of EAT showed that the reduced fat mass in *Camk2n1*^−/−^ rats was associated with a reduction in adipocyte number rather than adipocyte volume (Figure 3B).

To determine whether *Camk2n1* knockout altered CaMKII activity, visceral fat respiration, and whole-body adipokine production, we analyzed CaMKII activity, cellular energetics in epididymal adipocytes, and quantified serum concentrations of high molecular weight adiponectin and leptin in SHR and *Camk2n1*^−/−^ rats. EAT CaMKII activity was similar in SHR and *Camk2n1*^−/−^ rats (Figure 3D). Mitochondrial oxygen consumption: basal, ATP-linked, maximal, and leak respiration were similar in SHR and *Camk2n1*^−/−^ adipocytes (Table S3). However, we found a 20% decrease (*P*=0.038) in circulating HMW adiponectin and a significant 70% decrease (*P*=0.01) in circulating leptin (Table). Given the reduction in fat mass and circulating leptin, we assessed food intake, body temperature, and locomotor activity as indicators of energy homeostasis in SHR and *Camk2n1*^−/−^ rats. Food intake was reduced in *Camk2n1*^−/−^ from 6 to 8 weeks age, whereas from 9 to 10 weeks of age, food intake was greater compared with SHR (Figure 3D); across the 6 to 10 week period, average food intake was not significantly different (*P*>0.05; Figure 3D, inset). There were no significant differences in body temperature and locomotor activity (*P*>0.05; Figure S4B and S4C).

### In Vitro Camk2n1 Knockdown in 3T3-L1 Adipocytes

To establish whether Camk2n1 deficiency reduced adipogenic capacity, we knocked down *Camk2n1* expression in mouse 3T3-L1 fibroblasts. *Camk2n1* knocked down of 85% to 96% was confirmed from day 1 to 9 following lipofectamine treatment (Figure S5A) and was associated with a significant reduction in lipid formation assessed by oil red O staining intensity and reduction in the proportion of cells containing lipids by day 9 (*P*<0.05; Figure 3E). Markers of adipogenesis, *Cebpa* and *Pparg* were significantly altered by *Camk2n1* deficiency. *Cebpa* and *Pparg* expression increase from day 1 to 9 in scramble treated cells, whereas expression of these genes was significantly perturbed by *Camk2n1* siRNA by 63% (*P*<0.01) and 80%(*P*<0.001) on day 9, respectively (Figure 3F). By day 9, the mature adipocyte markers *Lep* and *Adipoq* in siRNA-treated cells were similar to control (Figure S5B).

### Transcriptomics

The molecular networks regulated by *Camk2n1* were defined by weighted gene coexpression analysis (WGCNA) of the SHR and *Camk2n1*^−/−^ LV and EAT transcriptomes. Differential expression was validated in LV and EAT by comparing expression of 12 genes (Table S4).

In LV, there were 192 differentially expressed genes (DEGs), 118 DEGs were ≥2-fold different between genotypes (*P*_adj_<0.05; Figure 4A; Table S5). Of the most significant DEG, *Kirrel3*, *Fabp4*, *Atf3*, *Capn3*, *Hdac4*, and *Plcb4* control cardiomyocyte size and function, whereas *Cfi*, *Irak3 Rnf144b*, and *Hspa8* regulate inflammatory processes. WGCNA of the LV transcriptome defined 8 modules with significant correlation to *Camk2n1* (Figure 4B). Kyoto Encyclopedia of Genes and Genomes functional enrichment analysis of these modules showed that *Camk2n1*^−/−^ LV are significantly enriched for cell function and maintenance, intracellular signaling, metabolism, and antigen presentation networks (Table S6). GO analysis showed that across modules, there were consistent themes relating to transcript and protein regulation (Table S7). Levels of FABP4 (fatty acid binding protein 4) in LV, a protein that regulates several enriched pathways, was reduced in *Camk2n1*^−/−^ compared with SHR (Figure 4C).

In EAT, there were 129 DEGs after adjustment for multiple testing; 90% of the most DEGs (≥2-fold) were downregulated with 20% associated with metabolic pathways (*Pla2g2a*, *P4ha3*, *Pla2g5*, *Slc39a8*, *Pcdh7*, and *Gcnt2*) and cell proliferation (*Cldn15*, *Fgf9*, *Diaph3*, and *Mki67*; Figure 4D; Table S8). WGCNA of the EAT transcriptome defined 8 modules correlated significantly with *Camk2n1* (Figure 4E; Table S9). Five modules were significantly enriched in *Camk2n1*^−/−^ EAT for KEGG pathways regulating cell maintenance and survival, and innate immunity and antigen presentation (Table S9), with transcript regulation and immune defense GO terms significantly enhanced (Table S10).

Soluble phospholipase A2 activity, which regulates adipogenesis and apoptosis, was reduced significantly in *Camk2n1*^−/−^ compared with SHR (Figure 4F).

### Human *CAMK2N1* Cis-eQTL and Cardiometabolic Trait Analysis

We analyzed human data from the GTEx and Type 2 Diabetes Knowledge Portals to investigate whether sequence variants that increase/decrease *CAMK2N1* expression were associated with cardiometabolic traits. We identified 263 cis-eQTLs regulating *CAMK2N1* (Table S11). The most significant single nucleotide polymorphism regulating *CAMK2N1* were found in Adipose–Visceral (VAT), in which there were 51 in total for this tissue. To test whether cis-eQTLs for *CAMK2N1* are enriched in VAT, we compared 1000 sets of 263 cis-eQTLs not associated with *CAMK2N1*. There were 21.7% of *CAMK2N1* cis-eQTLs in VAT, compared with 20.7% to 21.0% non-*CAMK2N1* cis-eQTLs in VAT in the 1000 sets of non-*CAMK2N1* cis-eQTLs (Figure S6).

**Figure 5.**
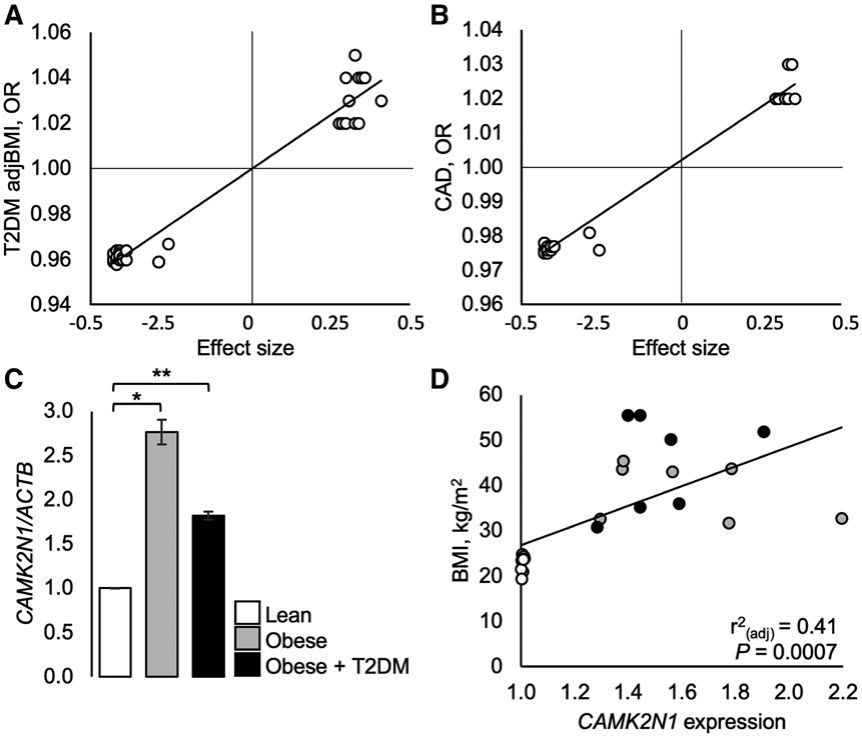
Expression quantitative trait locus (eQTLs) that correlate with cardiometabolic disease traits and *CAMK2N1* expression in human visceral fat. **A**, Variants in the GTex portal that alter *CAMK2N1* expression (defined by effect size) and correlate significantly with coronary artery disease (CAD). **B**, Variants in the GTex portal that alter *CAMK2N1* expression (effect size) and correlate significantly with type 2 diabetes mellitus adjusted for body mass index (T2DM adjBMI). **C**, *CAMK2N1* expression (normalized to *ACTB*) in visceral fat from lean, obese, and obese+T2DM subjects. Regression analysis: relative *CAMK2N1* expression in visceral fat with BMI. Mean±SEM n=10 lean, 9 obese, 9 obese+T2DM. Significant differences between lean and obese or obese+T2DM (*P=0.02 and **P<0.005). OR indicates odds ratio.

Of the *CAMK2N1* cis-eQTLs in VAT, 44 were significantly associated with cardiometabolic traits in the knowledge portals (Table S12). T2DM was the most frequent disease trait, linked to 39 variants (effect size range: −0.43 to 0.40, *P*_adj_=0.048–0.0052; Figure 5A), followed coronary artery disease linked to 27 variants (effect size range: −0.43 to 0.35, *P*_adj_=0.047–0.016; Figure 5B). All variants that are associated with *CAMK2N1* downregulation (negative effect size), are also associated with significant disease risk reduction, whereas variants that are associated with *CAMK2N1* upregulation (positive effect size), are associated with increased disease risk (Figure 5A and 5B; Table S11). To test whether *CAMK2N1* cis-eQTLs are enriched for cardiometabolic traits, we determined the association to traits of VAT cis-eQTLs in a randomly selected set of 263 cis-eQTLs not associated with *CAMK2N1* (Table S13). There was a significantly greater number of *CAMK2N1* cis-eQTLs associated with T2DM, coronary artery disease, and VAT volume than cis-eQTLs not associated with *CAMK2N1* (Table S14).

To establish the effect size of *CAMK2N1* expression on visceral fat mass (not reported in the knowledge portals, we quantified *CAMK2N1* expression in human visceral fat samples and found that compared with lean subjects, obese nondiabetics, and obese diabetics had a significantly greater body mass index, weight, and fat mass compared with lean subjects; although, BP across groups was not significantly different (Table S15). *CAMK2N1* expression in visceral fat was increased significantly by 1.82- to 2.76-fold in obese diabetic and nondiabetic subjects, respectively, compared to lean nondiabetics (effect size, obese =1.23, *P*=0.02, obese diabetic =1.56, *P*<0.005), but between obese groups, expression was similar (*P*>0.05; (Figure 5A). Regression analysis showed that *CAMK2N1* expression in visceral fat correlated significantly with body mass index (Figure 5D) and absolute fat mass (*r*^2^=0.34, *P*=0.0069).

## Discussion

The major results in this study demonstrate that *Camk2n1* knockout in SHR reduced CaMKII activity in the kidney and LV, but not in adipose tissue and that these changes lead to profound alterations in the cardiometabolic phenotype of the *Camk2n1*^−/−^ rat. Compared with SHR, *Camk2n1*^−/−^ rats had lower BP and increased vascular reactivity and lower LV mass and rate pressure product. *Camk2n1* knockout increased insulin sensitivity, whereas visceral fat mass in vivo and adipogenic capacity in vitro were decreased. These data support the previous eQTL and linkage studies in rat RI strains that showed strong associations between *Camk2n1* and these cardiometabolic traits.^3,4^

We show that reduced BP and increased vasoreactivity are associated with enhanced ACE2-Ang-(1–7)-Mas signaling in *Camk2n1*^−/−^ rats. In LV, CaMKII-associated prohypertrophic and upregulation antihypertrophic components of cell cycle were downregulated, specifying mechanisms for reduced LV mass, whereas in EAT, diminished pro-obesogenic cell cycle pathways and classical complement associated with insulin resistance provide mechanistic insights into reduced adiposity and ameliorated insulin sensitivity of *Camk2n1*^−/−^ rats. In human visceral fat, we found that *CAMK2N1* expression correlated with fat mass and body mass index, in keeping with previous reports of 34 cis-eQTLs (GTex Portal) that associated significantly with increased *CAMK2N1* expression and elevated risk of T2DM and coronary artery disease.

Experimental inhibition of CaMKII has been investigated to develop new treatments for hypertension and cardiac hypertrophy.^10–12,15^ In addition, indirectly, these and other studies have investigated the function of Camk2n1 while also testing CaMKII function. However, because of a number of off-target effects, including regulation of calcium signaling, masking docking sites on CaMKII, and inhibiting CaM-associated and other kinases, associated with KN-92, KN-93, autocamtide-derived inhibitory peptide (AC3-I), and CaMKIIN-tides, used in these inhibitor studies, there is an incomplete understanding of Camk2n1 function and its regulation of CaMKII.^12^ Furthermore, these inhibitory peptides are based on the inhibitory domain of CAMKIIN/Camk2n2, which has a different expression pattern and is likely conformationally and functionally distinct from endogenous Camk2n1. In our study, we show that endogenous Camk2n1 is required for full activity of CaMKII in kidney and LV but is dispensable for CaMKII activity in adipose tissue. We propose that the cardiorenal physiological and molecular effects of Camk2n1 deletion are, in part, likely determined by reduced CaMKII activity, but may also be due, at least in adipose tissue, to CaMKII-independent functions of Camk2n1.

Hypertension, like other features of MetS, has a polygenic basis and is controlled by multiple genetic variants in both rats and humans. For example, BP QTLs have been found on all rat chromosomes,^16^ just as GWAS hits for hypertension reside across all human chromosomes.^17^ Moreover, there is good agreement between rat BP QTLs and genes, and their syntenic regions and orthologs in humans, with the likelihood that the networks regulating BP are conserved across species.^16^ Hypertension can be mitigated by NO-mediated vasodilation through the ACE2-Ang(1–7)-Mas axis; in humans, *ACE2* and *NOS3* variants modulate BP, whereas renal eNOS deficiency in SHR, or ACE2 deletion in mice, contribute to hypertension and hypertension-related renal damage.^18–21^ CaMKIIN transfection in vitro into endothelial cells reduced Ca^2+^/CaM binding to eNOS and decreased NO production following bradykinin stimulation,^22^ whereas in vivo transgenic overexpression of *CaMKIIN* or AC3-I did not alter baseline BP nor NO-dependent vasodilation,^23^ only partially protecting from Ang II–induced hypertension, without affecting CaMKII overactivity.^7^ The relationship between CaMKII and ACE2 has not been elucidated. However, in our study, *Camk2n1* deletion reduced renal CaMKII activity, but increased renal ACE2 and eNOS, and their respective products, Ang-(1–7) and NO, which may, in part, be responsible for the lower BP in *Camk2n1*^−/−^. ACE2, eNOS, and CaMKII are regulated by Ca^2+^/CaM binding^24,25^; therefore, Camk2n1 may regulate the binding association of Ca^2+^/CaM with these enzymes, thereby affecting their activation and vasodilatory capacity.

CaMKII overactivity in humans with hypertension or T2DM is considered a cause of pathological LVH and heart failure.^10^ We found that *Camk2n1* deletion reduced cardiac CaMKII activity and LV mass in SHR and conferred partial protection from increased myocardial load. Thus, our data support previous studies showing reduced CaMKII activity ameliorates LV remodeling, but that this occurs through Camk2n1 deficiency. Our WGCNA defined altered hypertrophic pathways associate with *Camk2n1* knockout. For example, the cell cycle pathway that included inhibitors *Cdkn3* and *E2f8* was upregulated in *Camk2n1*^−/−^ LV.^26,27^ Furthermore, *Fabp4*/FABP4 were downregulated in *Camk2n1*^−/−^ LV and have been shown to promote cardiac hypertrophy in mice and regulate AMPK signaling, actin cytoskeleton, and oxidative phosphorylation,^28^ all of which were associated with *Camk2n1* deletion in our study.

Together, these changed pathways indicate mechanisms by which *Camk2n1* knockout has reduced LV mass and is protected from stress-related rate pressure product increases and remodeling through CaMKII modulation.

Insulin resistance, a key factor in MetS, was ameliorated in *Camk2n1*^−/−^ rats and occurs independently of CaMKII in adipose tissue. Improved insulin sensitivity is associated in humans with increased circulating NO^20^ and Ang-(1–7),^29^ and reduced leptin and visceral adiposity,^30^ all of which are features of *Camk2n1*^−/−^ rats. In addition, improved insulin sensitivity in SHR by *Camk2n1* deletion, mirrors the connection in humans, we found between cis variants that decrease *CAMK2N1* expression and are associated with increased insulin sensitivity.

In humans, we showed that *CAMK2N1* was reduced in visceral fat from lean compared with obese subjects, consistent with a previous study showing *CAMK2N1* upregulation in obese compared with lean Pima Indians.^31^ This is analogous to the reduced visceral fat and CaMKII-independent adipogenesis we have observed in *Camk2n1*^−/−^ rats. This is distinct from nonspecific CaMKII inhibitor studies suggesting CaMKII regulation of adipogenesis in vitro.^8,12^

Adipokine production is determined by adipocyte maturation, hypertrophy, and fat mass.^32^ Therefore, the reduced adiposity in *Camk2n1*^−/−^ rats is a likely cause of reduced adipokine production. Circulating adiponectin in humans has been found to correlate inversely with insulin resistance and T2DM.^33^ However, this relationship is inconsistent, with other studies that found elevated adiponectin increased T2DM risk and CVD mortality.^34,35^

WGCNA of the adipose transcriptome defined CaMKII-independent alterations in obesity- and MetS-related cell cycle, classical complement, and apoptosis pathways in *Camk2n1*^−/−^ rats. For example, downregulation in *Camk2n1*^−/−^ EAT of *Pla2g5* and *Pla2g2a* and reduced soluble phospholipase A2 activity supports the amelioration in MetS phenotypes in *Camk2n1*^−/−^ rats, as *Pla2g5* is upregulated in obese adipose tissue and promotes leptin secretion, whereas *Pla2g2a* is causally related to obesity and MetS.^30,36^ Moreover, soluble phospholipase A2 has been shown to promote adipogenesis and apoptosis associated with obesity.^30^ Apoptosis is an unlikely cause of reduced adiposity in *Camk2n1*^−/−^ rats as proapoptic genes (*Baiap2l1*/Birc5, *Dsg2*, and *Pcdh7*) were downregulated. Alternatively, reduced proliferation is suggested by upregulation of *Cdkn1a*/p21 in *Camk2n1*^−/−^ EAT, which has been shown elsewhere to prevent obesity and adipocyte hyperplasia,^37^ and downregulation of *Rbl1*/p107 and *Cdk1* that have been shown by others to be pro-obesogenic and upregulated in obesity.^38^ Furthermore, upregulation of *Id3* and downregulation of *C1s* and *C4* provide additional mechanisms for reduced adiponectin production,^39^ reduced adiposity, and increased insulin sensitivity.^32,40^

## Perspectives

This is the first study to report that in vivo deletion of *Camk2n1* diminishes CaMKII activity in kidney and heart, without affecting adipose CaMKII activity, and that *Camk2n1* deletion causes widespread ameliorations in cardiovascular and metabolic phenotypes. *Camk2n1* knockout in SHR, ameliorated multiple pathophysiological phenotypes including hypertension, LV mass, insulin sensitivity, and visceral adiposity, associated with reduced cardiorenal CaMKII activity and independent of adipose CaMKII activity. Together with our demonstration that visceral fat *CAMK2N1* expression increased in obese subjects and correlated with adiposity and our analysis of cis-acting variants that regulate human *CAMK2N1* and MetS traits, we conclude that Camk2n1 regulates multiple cardiovascular and metabolic processes, both dependently and independently of CaMKII, suggesting that endogenous Camk2n1/CAMK2N1 may not function exclusively as an inhibitor of CaMKII and requires a reappraisal of existing studies that have used nonspecific CaMKII inhibitors proposed to mimic Camk2n1 function. Furthermore, our data suggest that therapeutic targeting of CAMK2N1 may allow amelioration of MetS features in humans.

## Acknowledgments

We thank Nick Gilbert for support with the CaMKII (Ca^2+^/calmodulin-dependent kinase II) activity assay and Julie Moss for technical assistance. The shared university research facilities and Easter Bush Pathology provided histological and fluid analysis assistance. Edinburgh Genomics (Clinical), Edinburgh, carried out Whole genome sequencing. The Wellcome Trust Clinical Research Facility (WTCRF) and Edinburgh Genomics carried out RNA extraction and microarray hybridization, respectively. Human tissue samples were sourced from the Edinburgh Clinical Research Facility, and we acknowledge the financial support of National Health Service Research Scotland.

## Sources of Funding

P.M. Coan, M. Barrier, N. Alfazema, and A.G. Diaz are funded by an Advanced Grant ERC-2010-AdG_20100317 (ELABORATE) from the European Research Council awarded to T.J. Aitman. R.I. Menzies is supported by a British Heart Foundation Fellowship FS/15/60/31510. S.M. de Procé is funded by a Medical Research Council grant (MR/N005902/1), and R.H. Stimson is supported by the Medical Research Council (MR/K010271/1) and Chief Scientist Office (SCAF/17/02). R. Carter and N.M. Morton are funded by a Wellcome Trust New Investigator grant 100981/Z/13/Z awarded to N.M. Morton. Radiotelemetry equipment was funded by a Wellcome Trust Institutional Strategic Support Fund (ISSF2) award J22737 with additional support from the BHF Centre for Research Excellence, University of Edinburgh.

## Disclosures

T.J. Aitman has received speaker honoraria from and has research collaborations with Illumina and has received consultancy fees from AstraZeneca. The other authors report no conflicts.

## Supplementary Material

## Novelty and Significance

### What Is New?

- Camk2n1 negatively regulates blood pressure, left ventricle mass, and insulin sensitivity and is required for full adipogenic capacity in the spontaneously hypertensive rat.

### What Is Relevant?

- *Camk2n1* deletion has effects on multiple organ systems: lowering blood pressure and reducing renal CaMKII (Ca^2+^/calmodulin-dependent kinase II) activity, which is associated with increased ACE2 (angiotensin II–converting enzyme)-Ang-(1–7)-Mas activity, enhancing nitric oxide-related vasoreactivity; in the left ventricle, *Camk2n1* deletion reduced cardiac CaMKII activity, downregulating hypertrophy-related pathways, and preventing stress-induced increased myocardial load; and in visceral fat, Camk2n1, improved insulin sensitivity and reduced adipogenesis, by downregulating cell cycle pathways, independent of CaMKII.

### Summary

Camk2n1 regulates multiple organ systems associated with metabolic syndrome, and its ablation improved MetS features in spontaneously hypertensive rat. In humans, visceral fat *CAMK2N1* expression correlated with adiposity, and SNPs that regulate *CAMK2N1* expression are associated with coronary artery disease and type 2 diabetes mellitus.

## References

[1] Long MT, Fox CS (2016). The Framingham heart study–67 years of discovery in metabolic disease.. Nat Rev Endocrinol.

[2] Aitman TJ, Critser JK, Cuppen E (2008). Progress and prospects in rat genetics: a community view.. Nat Genet.

[3] Langley SR, Bottolo L, Kunes J, Zicha J, Zidek V, Hubner N, Cook SA, Pravenec M, Aitman TJ, Petretto E (2013). Systems-level approaches reveal conservation of trans-regulated genes in the rat and genetic determinants of blood pressure in humans.. Cardiovasc Res.

[4] Morrissey C, Grieve IC, Heinig M, Atanur S, Petretto E, Pravenec M, Hubner N, Aitman TJ (2011). Integrated genomic approaches to identification of candidate genes underlying metabolic and cardiovascular phenotypes in the spontaneously hypertensive rat.. Physiol Genomics.

[5] Shimoyama M, De Pons J, Hayman GT, Laulederkind SJ, Liu W, Nigam R, Petri V, Smith JR, Tutaj M, Wang SJ, Worthey E, Dwinell M, Jacob H (2015). The rat genome database 2015: genomic, phenotypic and environmental variations and disease.. Nucleic Acids Res.

[6] Vest RS, Davies KD, O’Leary H, Port JD, Bayer KU (2007). Dual mechanism of a natural CaMKII inhibitor.. Mol Biol Cell.

[7] Prasad AM, Morgan DA, Nuno DW, Ketsawatsomkron P, Bair TB, Venema AN, Dibbern ME, Kutschke WJ, Weiss RM, Lamping KG, Chapleau MW, Sigmund CD, Rahmouni K, Grumbach IM (2015). Calcium/calmodulin-dependent kinase II inhibition in smooth muscle reduces angiotensin II-induced hypertension by controlling aortic remodeling and baroreceptor function.. J Am Heart Assoc.

[8] Wang Hy, Goligorsky MS, Malbon CC (1997). Temporal activation of Ca2+-calmodulin-sensitive protein kinase type II is obligate for adipogenesis.. J Biol Chem.

[9] Yip MF, Ramm G, Larance M, Hoehn KL, Wagner MC, Guilhaus M, James DE (2008). CaMKII-mediated phosphorylation of the myosin motor Myo1c is required for insulin-stimulated GLUT4 translocation in adipocytes.. Cell Metab.

[10] Dewenter M, Neef S, Vettel C (2017). Calcium/Calmodulin-dependent protein kinase II activity persists during chronic β-adrenoceptor blockade in experimental and human heart failure.. Circ Heart Fail.

[11] Zhang R, Khoo MS, Wu Y (2005). Calmodulin kinase II inhibition protects against structural heart disease.. Nat Med.

[12] Pellicena P, Schulman H (2014). CaMKII inhibitors: from research tools to therapeutic agents.. Front Pharmacol.

[13] Kreusser MM, Lehmann LH, Keranov S (2014). Cardiac CaM Kinase II genes δ and γ contribute to adverse remodeling but redundantly inhibit calcineurin-induced myocardial hypertrophy.. Circulation.

[14] Ozcan L, Cristina de Souza J, Harari AA, Backs J, Olson EN, Tabas I (2013). Activation of calcium/calmodulin-dependent protein kinase II in obesity mediates suppression of hepatic insulin signaling.. Cell Metab.

[15] Li H, Li W, Gupta AK, Mohler PJ, Anderson ME, Grumbach IM (2010). Calmodulin kinase II is required for angiotensin II-mediated vascular smooth muscle hypertrophy.. Am J Physiol Heart Circ Physiol.

[16] Padmanabhan S, Joe B (2017). Towards precision medicine for hypertension: a review of genomic, epigenomic, and microbiomic effects on blood pressure in experimental rat models and humans.. Physiol Rev.

[17] Warren HR, Evangelou E, Cabrera CP, International Consortium of Blood Pressure (ICBP) 1000G Analyses; BIOS Consortium; Lifelines Cohort Study; Understanding Society Scientific group; CHD Exome+ Consortium; ExomeBP Consortium; T2D-GENES Consortium; GoT2DGenes Consortium; Cohorts for Heart and Ageing Research in Genome Epidemiology (CHARGE) BP Exome Consortium; International Genomics of Blood Pressure (iGEN-BP) Consortium; UK Biobank CardioMetabolic Consortium BP working group (2017). Genome-wide association analysis identifies novel blood pressure loci and offers biological insights into cardiovascular risk.. Nat Genet.

[18] Patel SK, Wai B, Ord M, MacIsaac RJ, Grant S, Velkoska E, Panagiotopoulos S, Jerums G, Srivastava PM, Burrell LM (2012). Association of ACE2 genetic variants with blood pressure, left ventricular mass, and cardiac function in caucasians with type 2 diabetes.. Am J Hypertens.

[19] Zhou XJ, Vaziri ND, Zhang J, Wang HW, Wang XQ (2002). Association of renal injury with nitric oxide deficiency in aged SHR: prevention by hypertension control with AT1 blockade.. Kidney Int.

[20] Emdin CA, Khera AV, Klarin D (2018). Phenotypic consequences of a genetic predisposition to enhanced nitric oxide signaling.. Circulation.

[21] Liu Z, Huang XR, Chen HY, Fung E, Liu J, Lan HY (2017). Deletion of angiotensin-converting enzyme-2 promotes hypertensive nephropathy by targeting smad7 for ubiquitin degradation.. Hypertension.

[22] Murthy S, Koval OM, Ramiro Diaz JM, Kumar S, Nuno D, Scott JA, Allamargot C, Zhu LJ, Broadhurst K, Santhana V, Kutschke WJ, Irani K, Lamping KG, Grumbach IM (2017). Endothelial CaMKII as a regulator of eNOS activity and NO-mediated vasoreactivity.. PLoS One.

[23] Prasad AM, Nuno DW, Koval OM, Ketsawatsomkron P, Li W, Li H, Shen FY, Joiner ML, Kutschke W, Weiss RM, Sigmund CD, Anderson ME, Lamping KG, Grumbach IM (2013). Differential control of calcium homeostasis and vascular reactivity by Ca2+/calmodulin-dependent kinase II.. Hypertension.

[24] Schneider JC, El Kebir D, Chéreau C, Lanone S, Huang XL, De Buys Roessingh AS, Mercier JC, Dall’Ava-Santucci J, Dinh-Xuan AT (2003). Involvement of Ca2+/calmodulin-dependent protein kinase II in endothelial NO production and endothelium-dependent relaxation.. Am J Physiol Heart Circ Physiol.

[25] Lambert DW, Clarke NE, Hooper NM, Turner AJ (2008). Calmodulin interacts with angiotensin-converting enzyme-2 (ACE2) and inhibits shedding of its ectodomain.. FEBS Lett.

[26] Li J, Zhang C, Xing Y, Janicki JS, Yamamoto M, Wang XL, Tang DQ, Cui T (2011). Up-regulation of p27(kip1) contributes to Nrf2-mediated protection against angiotensin II-induced cardiac hypertrophy.. Cardiovasc Res.

[27] Poolman RA, Brooks G (1998). Expressions and activities of cell cycle regulatory molecules during the transition from myocyte hyperplasia to hypertrophy.. J Mol Cell Cardiol.

[28] Zhang J, Qiao C, Chang L, Guo Y, Fan Y, Villacorta L, Chen YE, Zhang J (2016). Cardiomyocyte overexpression of FABP4 aggravates pressure overload-induced heart hypertrophy.. PLoS One.

[29] Passos-Silva DG, Verano-Braga T, Santos RA (2013). Angiotensin-(1-7): beyond the cardio-renal actions.. Clin Sci (Lond).

[30] Sato H, Taketomi Y, Ushida A (2014). The adipocyte-inducible secreted phospholipases PLA2G5 and PLA2G2E play distinct roles in obesity.. Cell Metab.

[31] Lee YH, Nair S, Rousseau E, Allison DB, Page GP, Tataranni PA, Bogardus C, Permana PA (2005). Microarray profiling of isolated abdominal subcutaneous adipocytes from obese vs non-obese Pima Indians: increased expression of inflammation-related genes.. Diabetologia.

[32] Coelho M, Oliveira T, Fernandes R (2013). Biochemistry of adipose tissue: an endocrine organ.. Arch Med Sci.

[33] Li S, Shin HJ, Ding EL, van Dam RM (2009). Adiponectin levels and risk of type 2 diabetes: a systematic review and meta-analysis.. JAMA.

[34] Yaghootkar H, Lamina C, Scott RA, GENESIS Consortium; RISC Consortium (2013). Mendelian randomization studies do not support a causal role for reduced circulating adiponectin levels in insulin resistance and type 2 diabetes.. Diabetes.

[35] Liu G, Ding M, Chiuve SE, Rimm EB, Franks PW, Meigs JB, Hu FB, Sun Q (2016). Plasma levels of fatty acid-binding protein 4, retinol-binding protein 4, high-molecular-weight adiponectin, and cardiovascular mortality among men with type 2 diabetes: a 22-year prospective study.. Arterioscler Thromb Vasc Biol.

[36] Iyer A, Lim J, Poudyal H, Reid RC, Suen JY, Webster J, Prins JB, Whitehead JP, Fairlie DP, Brown L (2012). An inhibitor of phospholipase A2 group IIA modulates adipocyte signaling and protects against diet-induced metabolic syndrome in rats.. Diabetes.

[37] Naaz A, Holsberger DR, Iwamoto GA, Nelson A, Kiyokawa H, Cooke PS (2004). Loss of cyclin-dependent kinase inhibitors produces adipocyte hyperplasia and obesity.. FASEB J.

[38] Lopez-Mejia IC, Castillo-Armengol J, Lagarrigue S, Fajas L (2018). Role of cell cycle regulators in adipose tissue and whole body energy homeostasis.. Cell Mol Life Sci.

[39] Doran AC, Meller N, Cutchins A, Deliri H, Slayton RP, Oldham SN, Kim JB, Keller SR, McNamara CA (2008). The helix-loop-helix factors Id3 and E47 are novel regulators of adiponectin.. Circ Res.

[40] Zhang J, Wright W, Bernlohr DA, Cushman SW, Chen X (2007). Alterations of the classic pathway of complement in adipose tissue of obesity and insulin resistance.. Am J Physiol Endocrinol Metab.

