# Supplementary material for: Camk2n1 Is a Negative Regulator of Blood Pressure, Left Ventricular Mass, Insulin Sensitivity, and Promotes Adiposity
Source: Hypertension. 2019 Jul 22;74(3):687–96. doi: 10.1161/HYPERTENSIONAHA.118.12409 (PMC6686962; doi:10.1161/HYPERTENSIONAHA.118.12409)
Supplement: Supplementary file 1 [file hyp-74-687-s001.pdf]

ONLINE/DATA SUPPLEMENT

Camk2n1 is a negative regulator of blood pressure, left ventricular mass, insulin sensitivity and promotes adiposity

Neza Alfazema[1†], Marjorie Barrier[1†], Sophie Marion de Procé[1], Robert I. Menzies[2], Roderick Carter[2], Kevin Stewart[2], Ana Garcia Diaz[3], Ben Moyon[3], Zoe Webster[3], Christopher O.C. Bellamy[4], Mark J. Arends[4], Roland H. Stimson[2], Nicholas M. Morton[2], Timothy J. Aitman[1] and Philip M. Coan[1*]

[1] MRC Institute of Genetics & Molecular Medicine, Edinburgh, EH4 2XU, UK.
[2] Centre for Cardiovascular Science, Queen's Medical Research Institute, University of Edinburgh, EH16 4TJ, UK.
[3] MRC London Institute of Medical Sciences, Imperial College London, London, W12 0NN, UK.
[4] Division of Pathology & Centre for Comparative Pathology, Edinburgh CRUK Cancer Centre, Edinburgh, EH4 2XR, UK.
Short title: Camk2n1 regulates metabolic syndrome traits
[†]Equal contribution
*Corresponding author:
Dr Philip M. Coan, MRC Institute for Genetics and Molecular Medicine, Edinburgh, EH4 2XU, UK.
+44(0)131-242-6690
p.m.coan.02@cantab.net

Methods

All data, analytical methods and study materials have been presented within this manuscript.

Rats

SHR-*Camk2n1*$^{-/-}$ knockout rats (referred to hereafter as *Camk2n1*$^{-/-}$ rats) were generated on an SHR/NCrl background (Charles River, Margate, UK), by microinjecting zinc-finger nuclease (ZFN) mRNA (Sigma), targeted to exon 1 of *Camk2n1*, into one-cell stage SHR/NCrl embryos that were implanted into pseudopregnant rats. Heterozygous progeny, from a founder harboring a 38bp deletion in *Camk2n1*, were intercrossed to generate homozygous knockout rats, confirmed by whole genome sequencing (WGS), PCR and Immunoblot (Fig. S1A-C). A search for off-target events, conducted by WGS of F_0 and analysed as described previously [1], detected three predicted non-synonymous coding variants; sequencing of progeny showed that two of these were not inherited. The third variant (Arg236His), in *Slc16a12*, confirmed by Sanger sequencing, was present in 11 rats that were phenotyped. *In silico* analysis of the variant showed no homology between *Slc16a12* and *Camk2n1*, that the amino acid encoded by the variant is poorly conserved between species and leads to a predicted non-deleterious conservative amino acid change. Further, no significant effects of the variant were detected on any of the phenotypes characterized in these studies. We, therefore, analysed all *Camk2n1*$^{-/-}$ rats together in this report. Age-matched 6- to 15-week-old rats were housed in open cages with free access to food and water. All procedures were carried out in accordance with UK Home Office regulations. Where possible assignment of animals to groups and analysis was random and blinded. End points and exclusion criteria were established prior to initiating experiments.

Human participants

Visceral adipose tissue was obtained intraoperatively from 28 subjects who were attending the Royal Infirmary of Edinburgh for elective abdominal surgery for nonmalignant disease. Subjects were divided into three groups (lean non-diabetic, obese non-diabetic, obese with T2DM; n=10, 9 and 9 respectively); T2DM was diagnosed based on clinical criteria. Adipose tissue collection was performed under the Lothian NRS Human Annotated Bioresource (15/ES/0094) following ethical approval. Written informed consent from each participant was obtained. Tissues were stored on dry ice then at -80°C.

Body weight and food intake

Food intake was measured in group-housed rats daily for 4 weeks from adolescences to adulthood (6-10 weeks). Body weights were measured at 15 weeks for tissue analysis, oral glucose testing and telemetric analyses.

Serum, urine and tissue analysis

Serum biochemistry and urinalysis (n=10/group) was carried out by the Veterinary Pathology Laboratory, Edinburgh. In-house ELISAs were used to determine serum leptin (Merck Millipore), high-molecular-weight (HMW) adiponectin (AMSBiotech) and nitric oxide (NO) concentrations (Abcam ab65328) and secretory phospholipase A2 (sPLA2) activity kit (Abcam) (n=5-7/group). Concentrations of endothelial nitric oxide synthase (eNOS) (AMS Biotechnology Ltd) and angiotensin-(1-7) (Ang-(1-7)

(Cusabio) and angiotensin II converting enzyme (ACE2) activity (BioVision) were also assayed in serum and kidney (n=5-7/group). Concentrations of fatty acid binding protein 4 (FABP4) were determined in left ventricle protein isolates (AMS Biotech). Tissue wet masses (Table S1) were normalized to body weight.

Cardiovascular physiology and cardiac morphology
Blood pressure and heart rate data were collected by radiotelemetry from transmitters implanted into rats (n=13/group) according to manufacturer's instructions (HD-S10, Data Sciences International), isoproterenol was infused to induce hypertrophy (n=5-8/group) and rate pressure product (RPP) calculated as described previously [1].
Vascular reactivity *in vivo* was performed as described previously [2] and the effect of acetylcholine (5g/kg; Sigma-Aldrich) on blood pressure was determined before and following administration of N-nitro-L-arginine methyl ester hydrochloride (L-NAME; 10mg/kg; Sigma-Aldrich). Saline vehicle or compound was administered (150µl/injection, 0.1, 1.0 & 10µg/kg) followed by catheter flushing with saline equal to the catheter volume (n=5-7/group). A second experiment was performed in another cohort of rats, in which L-NAME was substituted withthe Mas receptor antagonist A-779 (10mg/kg, Tocris Bioscience, Abingdon, UK).
LV histological analysis was carried out as reported previously [1] with cardiomyocyte number estimated using QuPath [3].

Glucose homeostasis and adipose tissue metabolism
Oral glucose tolerance tests (OGTT), mean adipocyte cell volume and volume-weighted cell number were carried out blinded as described previously (n=7/group) [1]. Mitochondrial oxygen consumption in primary epididymal adipocytes (n=6/group) was assessed, blinded, as described previously in an XFe24 Seahorse Bioanalyser (Agilent) and oxygen consumption rate data calculated according to the manufacturer's instructions (Agilent Technologies LDA UK, Cheshire, UK) [1].

Adipocyte culture
3T3-L1 preadipocytes were cultured according to supplier's instructions (Zen Bio Inc.). At 80% confluence, cells were transfected using lipofectamine RNaiMAX (Thermo Fisher Scientific) and either TriFECTa DsiRNA kit (mm.Ri.Camk2n1.13) containing a pool of 3 predesigned Dicer substrate siRNAs (DsiRNA) targeting both exons of *Camk2n1* or scrambled negative control DsiRNA (Intergrated DNA Technologies). Formation of DsiRNA complexes was achieved by adding the DsiRNA mixture made with reduced serum media (Opti-MEM, Thermo Fisher Scientific) and lipofectamine mixture, containing Opti-MEM, for 20 min at room temperature. Culture medium was replaced by Opti-MEM before adding DsiRNA-lipid complexes (10nM) and incubating for 16 h at 37°C, 5% CO_2. Following knockdown, medium was replaced with differentiation media 1 (DMI: DMEM, 1% Penicillin streptomycin (Thermo Fisher Scientific), 10% FBS, 5 µg/ml insulin, 100 nM dexamethasone, 1 µM rosiglitazone and 250 µM IBMX (Sigma)). After 72 h DMI was replaced with differentiation media 2 (DMII: DMI without rosiglitazone and IBMX). After 48 h DMII was replaced with adipocyte maintenance medium (DMEM, 10% FBS and 1% penicillin-streptomycin) and incubated at 37°C, 5% CO_2. Media were changed every two days. Cells were harvested at 1, 4, and 9 days post-differentiation for analysis.

The proportion of lipid containing cells was estimated using a point grid randomly superimposed on three random light micrograph images of 3T3-L1 adipocytes stained with Oil red O of 2 wells per treatment from 3 separate experiments (images were blinded prior to statistical analysis). Points superimposed onto cells containing lipid droplets, cells without visible lipid droplets and background were counted (189 per well). Average point counts for each group were calculated per plate for statistical analysis [4].

CaMKII activity

Autonomous CaMKII activity was analysed in tissue homogenates from whole kidney, LV and epididymal fat using the SignaTECT Calcium/Calmodulin-Dependent Protein Kinase Assay System and following the manufacturer's instructions (Promega #TB279). [γ-^{32}P]ATP was purchased from Perkin Elmer (NEG002A100UC).

Transcriptomic and gene co-expression network analyses

Transcriptome analysis was carried out from total RNA extracted from whole LV and epididymal adipose tissue (EAT) (n=4/group), using RNeasy fibrous tissue and lipid mini kits (Qiagen). RNA was hybridized to Affymetrix Rat Gene 2.1 ST arrays and analysed by Affymetrix GeneTitan MC Scanner (Affymetrix Inc.). Data analysis was performed using R software v3.4.0 and Bioconductor packages. Raw expression data were log2 transformed, normalized by quantile normalization, and summarized at the transcript level [5]. Transcripts were considered robustly expressed if the median of the log2 transformed normalized expression was ≥4. Differential expression between *Camk2n1*$^{-/-}$ and SHR was assessed by linear regression followed by Benjamini-Hochberg correction, and validated using separate samples by qPCR [6, 7].

The 17,768 robustly expressed genes from the microarray analysis were analysed further by a weighted gene co-expression network reconstruction algorithm to create co-expression networks (WGCNA)[8]. Separately, the EAT and LV transcriptomes were analysed, and 27 sets of parameters tested in order to detect consistent patterns in the co-expression network modules and determine which were significantly associated with *Camk2n1*. For each of these modules, KEGG and GO functional enrichment was assessed using the enrichR package [9] to identify known biological networks and functions of the genes within the modules. A statistical threshold of $P<0.05$ was used to establish significant associations with Benjamini and Hochberg correction for multiple comparisons.

qPCR analysis

For targeted gene expression studies, RNA was extracted from 3T3-L1 adipocytes and rat tissues for qPCR, as described previously [10]. qPCR was performed using primers listed in Table S2. *Prpl1* was used as a reference gene for 3T3-L1, whereas *Actb* was used for adipose tissue and left ventricle (LV). LV transcripts from telemetric studies were normalized to *Hprt*, due to effects of isoproterenol on *Actb* expression. Ct values were compared using the $2^{-\Delta\Delta Ct}$ method.

Human *CAMK2N1* analysis

The GTEx Portal (https://gtexportal.org/home/) was used to identify tissue-specific significant *cis*-eQTLs associated with human *CAMK2N1* expression. Using R software and GTex data Release V7 (dbGaP Accession phs000424.v7.p2)

CAMK2N1 cis-eQTLs were compared to 1000 sets (equal in size to the total number of CAMK2N1 *cis*-eQTLs) of randomly selected *cis*-eQTLs not associated with *CAMK2N1* (non-*CAMK2N1 cis*-eQTLs), to determine whether there was enrichment in *CAMK2N1 cis*-eQTLs for a specific tissue.
The Cardiovascular Disease and Type 2 Diabetes Knowledge portals (01-04-2019: http://broadcvdi.org/home/portalHome ; http://www.type2diabetesgenetics.org/) were mined to determine whether *CAMK2N1 cis*-eQTLs from the GTEx portal were associated with cardiometabolic traits in humans. In addition, one set of *cis*-eQTLs not associated with *CAMK2N1*, but of equal size to the total number of *CAMK2N1 cis*-eQTLs was used to analyse cardiometabolic trait associations in Adipose-Visceral *cis*-eQTLs. Adipose-Visceral *CAMK2N1* and non-*CAMK2N1 cis*-eQTL cardimetabolic trait associations were compared.

Statistical analysis
Unpaired t-tests, 1-way or 2-way ANOVA followed by Fisher's LSD post hoc test were used to assess differences between SHR, *Camk2n1*$^{-/-}$ and treatment. Where possible, data collection and analysis were blinded. P<0.05 was considered significant. For vascular reactivity, U-tests were used to analyse mean blood pressure and a 2-way ANOVA was used to analyse area-under-the-blood-pressure curve (AUC_{BP}). For *in vitro* studies, either t-tests or Kruskall-Wallis tests were used. For Adipose-Visceral *CAMK2N1* and non-*CAMK2N1 cis*-eQTL trait comparison, chi-squared analysis was used. For human visceral fat analysis, unpaired t-tests and simple regression analysis were carried out. All statistics was performed using Minitab Express (v1.5.1).

Supplemental References

1. Coan PM, Barrier M, Alfazema N, et al. Complement factor b is a determinant of both metabolic and cardiovascular features of metabolic syndrome. *Hypertension*. 2017;70:624-633
2. Menzies RI, Zammit-Mangion A, Hollis LM, Lennen RJ, Jansen MA, Webb DJ, Mullins JJ, Dear JW, Sanguinetti G, Bailey MA. An anatomically unbiased approach for analysis of renal bold magnetic resonance images. *Am J Physiol Renal Physiol*. 2013;305:F845-852
3. Bankhead P, Loughrey MB, Fernandez JA, Dombrowski Y, McArt DG, Dunne PD, McQuaid S, Gray RT, Murray LJ, Coleman HG, James JA, Salto-Tellez M, Hamilton PW. Qupath: Open source software for digital pathology image analysis. *Sci Rep*. 2017;7:16878
4. Howard V, Reed MG. *Unbiased stereology : Three-dimensional measurement in microscopy*. Abingdon: BIOS Scientific; 2005.
5. Carvalho BS, Irizarry RA. A framework for oligonucleotide microarray preprocessing. *Bioinformatics*. 2010;26:2363-2367
6. Ritchie ME, Phipson B, Wu D, Hu Y, Law CW, Shi W, Smyth GK. Limma powers differential expression analyses for rna-sequencing and microarray studies. *Nucleic Acids Res*. 2015;43:e47
7. Phipson B, Lee S, Majewski IJ, Alexander WS, Smyth GK. Robust hyperparameter estimation protects against hypervariable genes and improves power to detect differential expression. *Ann Appl Stat*. 2016;10:946-963
8. Langfelder P, Horvath S. Wgcna: An r package for weighted correlation network analysis. *BMC Bioinformatics*. 2008;9:559
9. Kuleshov MV, Jones MR, Rouillard AD, Fernandez NF, Duan Q, Wang Z, Koplev S, Jenkins SL, Jagodnik KM, Lachmann A, McDermott MG, Monteiro CD, Gundersen GW, Ma'ayan A. Enrichr: A comprehensive gene set enrichment analysis web server 2016 update. *Nucleic Acids Res*. 2016;44:W90-97
10. Coan PM, Hummel O, Diaz AI, Barrier M, Alfazema N, Norsworthy PJ, Pravenec M, Petretto E, Huebner N, Aitman TJ. Genetic, physiological and comparative genomic studies of hypertension and insulin resistance in the spontaneously hypertensive rat. *Dis Model Mech*. 2017;10:297-306
11. Chu AY, Deng X, Fisher VA, et al. Multiethnic genome-wide meta-analysis of ectopic fat depots identifies loci associated with adipocyte development and differentiation. *Nat Genet*. 2017;49:125-130
12. Walford GA, Gustafsson S, Rybin D, et al. Genome-wide association study of the modified stumvoll insulin sensitivity index identifies bcl2 and fam19a2 as novel insulin sensitivity loci. *Diabetes*. 2016;65:3200-3211

Tables

Table S1 Tissue wet masses

Organ (g/kg)	SHR	*Camk2n1*$^{-/-}$
Heart	3.95 ±0.01	3.87 ±0.05
Lungs	4.17 ±0.04	4.18 ±0.10
Adrenal glands	0.150 ±0.004	0.153 ±0.003
Kidneys	6.90 ±0.11	6.92 ±0.11
Liver	27.1 ±0.3	27.4 ±0.2
Spleen	1.79 ±0.02	1.86 ±0.03

Mean ±SEM n=10.

Table S2. Primers

Transcript	Forward primer	Reverse primer
Acta	CGCCATCAGGAACCTCGAGAA	TCCCACGATGGATGGGAAAACA
Actb	ATGTACCCAGGCATTGCTGAC	GAGTACTTGCGCTCAGGAGGA
ACTB	CTATAAAACCCAGCGGCGCGA	ATCATCCATGGTGAGCTGGCGG
Actc	CAAAGCACGCCTACAGATCCCA	GAAGACAGCTCTGGGAGCATCA
Adipoq	CTCCACCCAAGGAAACTTGTGC	TTAGGACCAAGAACACCTGCGT
Atf3	GGCGGCGGGAAAGAAACAAAAT	TCTGACTCCTTCTGCAGGCACT
Bbs1	CTGCTGCTTCTGGTGCTAAG	TGGAGGTAGGTGGATCAGGA
Btg2	GCCGTAGGTTTCCTCACCAGTC	TTGTAATGATCGGTCAGTGCGT
Camk2n1	GGAGCAAGCGCGTTGTTATTGA	ACAGCCCGCCACTCTTCTTATT
CAMK2N1	CGGAGCAAGCGGGTTGTTATT	GCCAATAACTGTTACCGCCGTT
Camk2n1-KO	TGCAGGACACCAACAACTTC	GTGCTTTCTCCTCCTCATGC
Ccnb2	AAGGAAGAGAGCCTCTGCCAAG	GGACTGCAAAGCCTCAAGCTG
Cebpa	AGGTGCTGGAGTTGACCAGT	CAGCCTAGAGATCCAGCGAC
Fam134b	GAGCCTCAGTGAAAGCTGGGAA	AGGCAAAACTTGCCAGGGCT
Fasn	CATTCCAGGTAAATGGGCCAGC	TCCCAGAGGAAGTCGGGTGATA
Grtp1	AGAGGGCGATCAAATGGTCCAA	CGGTTCAAGTCTGTCACGCTTT
Hprt	TCAGTCCCAGCGTCGTGATTAG	TCGAGCAAGTCTTTCAGTCCTGT
Jun	CCTTCTACGACGATGCCCTCAA	AGGTTCAAGGTCATGCTCTGCT
Lep	CAGCAGCTGCAAGGTCCAAGA	TAGGACCAAAGCCACAGGAACC
Mgll	GACTTTGAAGGTCCTTGCTGCC	CAGATGAGTGGGTCGGAGTTGT
Myo1c	CTTGCTGACCAGAAGACCAGGA	ACATGGTCTCCTTCAGGTTCCG
Nppb	ACAATCCACGATGCAGAAGCTG	GAAGGCGCTGTCTTGAGACCTA
Pkhd1l1	TCACGGTCTGCCTATTCCTGTG	ACTGTGTCTGTGTCCTGTGCTT
Pla2g5	CCTGTGTTGCAGCTTGTAGACC	GCGCTTCATTTCTTGGGTTCTTTT
Pla2g2a	AAGGCAGGCCCTTGAACAAGAA	TGCTGTCAGCTCTCTTGGATGG
Pparg	GGCTGAGGAGAAGTCACACTCT	ACCGCTTCTTTCAAATCTTGTCTGT
Prrt3	GTTCAGGCAGGTCTCAAACG	ATTAGGGTGATGGTGCCTCC
Prlp1	*GGCTGCCGAAGATGGCGGAG*	*GCCTTCACAGCGTACGACCACC*
Serpin3n	AACCCTGAACATCAGGAGTCGG	CTTCAGGCTGCAGAGCCGATA
Slc2a4	TTTGCACACCACTTCCGAAGGC	GGTTCCCCATCTTCAGAGCCGAT
Slc39a8	ACGTCACCCAGATAACCAGCTC	GCCCCAGACTTCGGAAAAACTG
Tnf	CTGTGCCTCAGCCTCTTCTCAT	AGCCCATTTGGGAACTTCTCCT
Rbfox3	ATTCCAACTTACGGAGCGGCAC	AAACGGACAAGAGAGTGGTGGG

Table S3. Primary epididymal adipocyte mitochondrial respiration from SHR v *Camk2n1*$^{-/-}$ rats

Respiration (pmol/min/cell)	SHR	*Camk2n1*$^{-/-}$
Basal	0.15 ±0.03	0.13 ±0.04
Maximal	0.46 ±0.07	0.46 ±0.14
ATP-linked	0.09 ±0.02	0.08 ±0.02
Reserve	0.31 ±0.05	0.31 ±0.10
Leak	0.06 ±0.01	0.04 ±0.02

Mean ±SEM n=6.

Table S4. Differentially expressed genes in EAT and LV samples from SHR and *Camk2n1*$^{-/-}$ rats by microarray and qPCR analysis

Transcript ID	FC* (Affymetrix)	P_{adj} †	FC (qPCR)	*P*-value
Btg2	-2.68	3.40e-04	-1.25	2.30e-02
Jun	-2.08	5.60e-03	-1.54	2.50e-02
Fam134b	4.43	6.10e-03	2.60	4.00e-03
Rbfox3	-2.04	1.45e-03	-3.13	1.06e-06
Parpbp	-2.09	7.45e-03	-2.00	7.01e-03
Pla2g5	-2.35	7.45e-03	-1.45	2.34e-02
Pla2g2a	-6.46	7.45e-03	-2.78	1.31e-04
Atf3	-4.76	8.34e-03	-2.63	2.04e-05
Cldn15	-3.20	1.51e-02	-1.54	3.12e-02
Slc39a8	-2.27	1.11e-02	-1.47	3.61e-05
Serpina3n	5.41	1.61e-02	2.78	3.11e-02
Pkhd1l1	-3.10	3.99e-02	-1.86	2.00e-02

Abbreviations: EAT, epididymal adipose tissue; qPCR, quantitative PCR; LV, left ventricle.
*Fold change (FC), *Camk2n1*$^{-/-}$/SHR; †Benjamini-Hochberg adjusted *P*-value.

Table S5. Significantly differentially expressed genes in *Camk2n1*$^{-/-}$ vs SHR LV

Gene ID	FC*	t	Raw P-value	P_{adj} †	B‡
Btg2	-2.68	9.04	1.11E-08	9.88E-05	9.75
Ccnb2	2.71	-8.20	5.61E-08	3.32E-04	8.31
Med27	-1.76	7.80	1.24E-07	4.41E-04	7.60
Hist1h1d	2.02	-7.68	1.61E-07	4.76E-04	7.37
Tjp1	3.70	-7.17	4.59E-07	6.28E-04	6.42
Ces2	4.30	-6.91	8.05E-07	9.35E-04	5.91
Mmp28	1.99	-6.89	8.42E-07	9.35E-04	5.86
Fchsd2	4.13	-6.85	9.17E-07	9.47E-04	5.79
Hist2h2ac	1.60	-6.83	9.60E-07	9.47E-04	5.74
Kdsr	4.28	-6.80	1.02E-06	9.55E-04	5.69
Gpx6	3.14	-6.59	1.58E-06	1.41E-03	5.28
RGD1560883	8.81	-6.52	1.85E-06	1.47E-03	5.14
Cfi	4.68	-6.51	1.90E-06	1.47E-03	5.12
Mir210	-2.78	6.53	1.84E-06	1.47E-03	5.15
Hist1h2bf	2.43	-6.28	3.17E-06	1.94E-03	4.64
Eif4e	-1.72	6.20	3.78E-06	2.24E-03	4.48
Hist1h2bcl1	2.27	-6.04	5.47E-06	2.68E-03	4.14
Jun	-2.08	6.03	5.58E-06	2.68E-03	4.12
Hdac4	2.97	-5.98	6.18E-06	2.75E-03	4.03
Ube2c	2.01	-5.97	6.41E-06	2.78E-03	3.99
Fam134b	4.43	-5.84	8.55E-06	3.30E-03	3.72
Hist2h3c2	1.81	-5.82	8.91E-06	3.37E-03	3.69
Fabp4	-7.07	-5.73	1.09E-05	3.81E-03	3.49
Hist1h1a	1.75	-5.73	1.09E-05	3.81E-03	3.50
Tmem144	3.04	-5.68	1.24E-05	3.90E-03	3.38
Hist1h3a	2.08	-5.69	1.21E-05	3.90E-03	3.40
Csnka2ip	41.93	-5.58	1.54E-05	4.43E-03	3.17
Plcb4	-2.73	5.57	1.59E-05	4.45E-03	3.15
5srrna	2.70	-5.55	1.67E-05	4.56E-03	3.10
Suclg2	36.03	-5.49	1.91E-05	4.58E-03	2.98
Smpd4	12.25	-5.50	1.86E-05	4.58E-03	3.00
Slc30a7	5.34	-5.51	1.83E-05	4.58E-03	3.02
Ect2	2.16	-5.51	1.83E-05	4.58E-03	3.02
LOC102549061	2.13	-5.52	1.80E-05	4.58E-03	3.03
Atf3	-4.76	5.48	1.94E-05	4.60E-03	2.96
Mir3597-1	5.79	-5.45	2.08E-05	4.80E-03	2.90
Jmjd6	-1.76	5.40	2.36E-05	5.30E-03	2.78
Elavl2	24.93	-5.38	2.48E-05	5.51E-03	2.73
Grtp1	-2.38	5.33	2.77E-05	5.92E-03	2.63
Arhgap11a	1.83	-5.27	3.23E-05	6.44E-03	2.49
Mir466d	-8.41	5.28	3.15E-05	6.44E-03	2.51

Cenpe	1.75	-5.25	3.35E-05	6.56E-03	2.45
Kif18b	2.30	-5.19	3.82E-05	7.08E-03	2.33
Timm17a	-1.51	5.19	3.83E-05	7.08E-03	2.33
N5	-1.96	5.21	3.68E-05	7.08E-03	2.36
LOC102550734	-2.41	5.21	3.72E-05	7.08E-03	2.35
Tcrva8	1.89	-5.15	4.19E-05	7.60E-03	2.24
Abcc12	7.38	-5.13	4.39E-05	7.72E-03	2.20
Hspa8	-2.24	5.11	4.60E-05	7.93E-03	2.16
Top2a	2.18	-5.10	4.80E-05	8.20E-03	2.12
Mki67	2.12	-5.09	4.90E-05	8.29E-03	2.10
RGD1307443	25.73	-5.07	5.11E-05	8.48E-03	2.06
Cdkn3	1.99	-5.07	5.09E-05	8.48E-03	2.06
Zfp560	4.01	-5.03	5.57E-05	8.94E-03	1.98
Arl6ip1	1.40	-5.03	5.60E-05	8.94E-03	1.97
LOC689458	2.24	-5.01	5.84E-05	9.18E-03	1.93
LOC684828	1.69	-4.99	6.11E-05	9.36E-03	1.89
LOC100910714	-3.66	4.97	6.43E-05	9.76E-03	1.84
Fermt2	3.82	-4.92	7.31E-05	1.06E-02	1.72
Vom1r74	2.23	-4.87	8.11E-05	1.16E-02	1.62
E2f8	1.71	-4.86	8.38E-05	1.17E-02	1.59
LOC100911282	1.70	-4.87	8.25E-05	1.17E-02	1.61
Ifrd1	-1.47	4.85	8.51E-05	1.17E-02	1.58
Bcl2l1	-3.58	4.82	9.15E-05	1.21E-02	1.51
Hbq1b	2.02	-4.79	9.80E-05	1.27E-02	1.45
Klhl18	-1.51	4.78	1.01E-04	1.28E-02	1.42
Nav2	2.09	-4.77	1.04E-04	1.31E-02	1.39
Tspyl2	-1.63	4.76	1.07E-04	1.33E-02	1.37
Cyp2c22	-2.60	4.75	1.09E-04	1.35E-02	1.35
Zbtb10	-1.42	4.74	1.11E-04	1.36E-02	1.33
Gcgr	-1.59	4.74	1.13E-04	1.37E-02	1.32
Irak3	3.87	-4.73	1.15E-04	1.40E-02	1.29
Kirrel3	11.83	-4.71	1.19E-04	1.41E-02	1.27
Prdm9	-1.82	4.71	1.20E-04	1.42E-02	1.26
Ccne1	1.56	-4.69	1.25E-04	1.45E-02	1.22
Gna1	2.09	-4.65	1.37E-04	1.57E-02	1.13
Kif23	1.67	-4.65	1.38E-04	1.57E-02	1.13
Igf2r	-1.70	4.64	1.42E-04	1.60E-02	1.10
Dkk2	1.53	-4.63	1.44E-04	1.61E-02	1.09
Gnb4	1.45	-4.62	1.47E-04	1.63E-02	1.07
Fbxo5	1.51	-4.60	1.56E-04	1.67E-02	1.01
Serpinh1	6.50	-4.59	1.58E-04	1.67E-02	1.00
Atp6v0a2	2.19	-4.58	1.62E-04	1.70E-02	0.97
AABR07027342.1	1.83	-4.56	1.72E-04	1.77E-02	0.92

Capn3	-3.02	-4.53	1.83E-04	1.85E-02	0.86
Ucp3	1.66	-4.53	1.84E-04	1.85E-02	0.86
Rad54b	3.01	-4.51	1.91E-04	1.91E-02	0.82
AABR07049040.1	3.28	-4.48	2.06E-04	1.94E-02	0.75
Serpina3n	2.16	-4.50	1.99E-04	1.94E-02	0.79
Sucla2	1.93	-4.50	1.99E-04	1.94E-02	0.78
LOC685716	1.77	-4.50	1.99E-04	1.94E-02	0.79
LOC690840	-2.69	4.49	2.01E-04	1.94E-02	0.77
Cdk1	1.97	-4.47	2.10E-04	1.95E-02	0.73
Adh5	-1.45	4.48	2.08E-04	1.95E-02	0.74
Cenpf	1.89	-4.44	2.26E-04	2.07E-02	0.66
Soga3	-2.96	4.43	2.34E-04	2.12E-02	0.63
Zfp697	-1.71	4.41	2.47E-04	2.19E-02	0.58
AABR07025010.1	-1.81	4.40	2.49E-04	2.20E-02	0.58
AABR07007130.1	2.36	-4.39	2.57E-04	2.24E-02	0.54
LOC100912233	2.07	-4.37	2.68E-04	2.30E-02	0.51
Bbs5	-1.51	4.35	2.79E-04	2.38E-02	0.47
RGD1563231	3.11	-4.35	2.82E-04	2.38E-02	0.46
Jchl1	3.47	-4.34	2.88E-04	2.40E-02	0.44
Mir324	1.84	-4.34	2.92E-04	2.43E-02	0.43
Thumpd1	3.60	-4.33	2.97E-04	2.43E-02	0.41
Cldn22	1.83	-4.33	2.97E-04	2.43E-02	0.41
LOC102554602	2.32	-4.31	3.10E-04	2.48E-02	0.37
LOC684841	1.58	-4.31	3.10E-04	2.48E-02	0.37
Hydin	1.35	-4.31	3.11E-04	2.48E-02	0.37
Nop58	-3.69	4.31	3.10E-04	2.48E-02	0.37
Rps12	1.53	-4.30	3.15E-04	2.50E-02	0.36
Adam1a	-1.44	4.30	3.17E-04	2.50E-02	0.35
Kif20a	1.78	-4.28	3.30E-04	2.58E-02	0.31
LOC100359616	-1.77	4.27	3.38E-04	2.63E-02	0.29
Yipf5	-1.42	4.25	3.56E-04	2.72E-02	0.24
Alox12b	2.16	-4.22	3.86E-04	2.78E-02	0.17
Fam64a	1.88	-4.23	3.79E-04	2.78E-02	0.18
Dapl1	1.65	-4.22	3.87E-04	2.78E-02	0.16
Kif11	1.52	-4.22	3.88E-04	2.78E-02	0.16
Cog3	-1.34	4.24	3.68E-04	2.78E-02	0.21
Eml3	-1.45	4.22	3.87E-04	2.78E-02	0.16
LOC100359563	-2.57	4.22	3.89E-04	2.78E-02	0.16
Mapkap1	1.66	-4.21	3.98E-04	2.81E-02	0.14
Crym	1.63	-4.20	4.05E-04	2.84E-02	0.12
LOC100912026	3.84	-4.19	4.12E-04	2.87E-02	0.10
Lage3	1.51	-4.19	4.13E-04	2.87E-02	0.10
Adamts1	-1.72	4.19	4.15E-04	2.87E-02	0.10

LOC688899	1.83	-4.17	4.36E-04	2.97E-02	0.05
Hmgcr	-1.50	4.16	4.41E-04	2.99E-02	0.04
Tll1	-1.63	4.15	4.55E-04	3.04E-02	0.01
Mir99a	-1.87	4.15	4.51E-04	3.04E-02	0.02
Angptl4	1.76	-4.14	4.68E-04	3.09E-02	-0.02
Lrfn5	1.65	-4.12	4.93E-04	3.20E-02	-0.06
Ier3	-1.46	4.12	4.94E-04	3.20E-02	-0.07
Mirlet7b	2.65	-4.11	5.05E-04	3.26E-02	-0.09
Fev	1.42	-4.09	5.22E-04	3.35E-02	-0.12
Iqgap3	1.82	-4.08	5.36E-04	3.41E-02	-0.14
Hspa9	-1.78	4.08	5.34E-04	3.41E-02	-0.14
Lrig2	-1.54	4.08	5.40E-04	3.43E-02	-0.15
Taf1d	-1.89	4.08	5.44E-04	3.44E-02	-0.16
LOC100360754	1.66	-4.06	5.62E-04	3.54E-02	-0.19
Mcfd2	-1.37	4.05	5.79E-04	3.61E-02	-0.22
Rnf144b	-3.78	4.05	5.80E-04	3.61E-02	-0.22
Cep295	2.22	-4.03	6.01E-04	3.71E-02	-0.25
Ccar1	-1.46	4.03	6.07E-04	3.72E-02	-0.26
Elf4	-1.55	4.02	6.16E-04	3.72E-02	-0.27
Taf1b	2.57	-4.02	6.21E-04	3.74E-02	-0.28
Urad	1.65	-4.01	6.30E-04	3.78E-02	-0.29
Apcdd1	1.68	-4.00	6.49E-04	3.88E-02	-0.32
Mir181d	1.82	-4.00	6.57E-04	3.91E-02	-0.33
Fv1	-2.12	3.99	6.70E-04	3.93E-02	-0.35
Sumo2	1.62	-3.98	6.86E-04	4.00E-02	-0.37
Triobp	1.49	-3.97	6.95E-04	4.03E-02	-0.38
Bphl	-1.42	3.97	7.01E-04	4.03E-02	-0.39
Cyp2a1	2.66	-3.97	7.08E-04	4.04E-02	-0.40
Tp73	1.47	-3.97	7.08E-04	4.04E-02	-0.40
Apbb3	-1.34	3.96	7.10E-04	4.04E-02	-0.40
Arhgap15	-1.52	3.96	7.15E-04	4.06E-02	-0.41
Ermard	-1.46	3.95	7.29E-04	4.09E-02	-0.43
Eftud1	1.68	-3.94	7.47E-04	4.10E-02	-0.45
Zcchc7	-1.50	3.94	7.52E-04	4.10E-02	-0.46
Exosc4	-1.55	3.94	7.51E-04	4.10E-02	-0.46
Btbd11	-1.65	3.94	7.51E-04	4.10E-02	-0.46
Aptx	-1.50	3.93	7.62E-04	4.14E-02	-0.47
Cenpt	1.62	-3.93	7.67E-04	4.14E-02	-0.48
Mcts1	-1.63	3.92	7.85E-04	4.22E-02	-0.50
LOC100911291	2.47	-3.91	8.00E-04	4.27E-02	-0.52
Itpr2	-1.23	3.90	8.23E-04	4.32E-02	-0.54
LOC100362987	-1.48	3.89	8.41E-04	4.36E-02	-0.56
Ralgapb	-2.32	3.89	8.41E-04	4.36E-02	-0.56

LOC100360439	-1.99	3.89	8.53E-04	4.37E-02	-0.58
Cnot11	-1.33	3.88	8.64E-04	4.40E-02	-0.59
Cdc20	1.82	-3.88	8.77E-04	4.43E-02	-0.60
U2surp	1.49	-3.87	8.96E-04	4.43E-02	-0.62
Lamtor2	-1.29	3.87	8.92E-04	4.43E-02	-0.62
Ap3b1	-1.36	3.87	8.96E-04	4.43E-02	-0.62
LOC499339	-1.50	3.87	8.92E-04	4.43E-02	-0.62
Yars2	-1.64	3.87	8.95E-04	4.43E-02	-0.62
Med14	-1.74	3.87	8.88E-04	4.43E-02	-0.61
Mdm4	-1.41	3.86	9.15E-04	4.48E-02	-0.64
LOC363337	3.11	-3.85	9.26E-04	4.51E-02	-0.65
AABR07059215.1	2.20	-3.84	9.48E-04	4.59E-02	-0.67
LOC100363177	-1.88	3.83	9.81E-04	4.67E-02	-0.71
Foxo3	-1.88	3.82	9.92E-04	4.69E-02	-0.72
Tomm5	-1.46	3.81	1.02E-03	4.78E-02	-0.74
Ube2g2	-1.34	3.80	1.05E-03	4.88E-02	-0.77
Amt	-1.38	3.80	1.04E-03	4.88E-02	-0.76
Mcpt1l2	3.27	-3.80	1.06E-03	4.90E-02	-0.78
Cit	1.41	-3.80	1.06E-03	4.90E-02	-0.78
Zfp385a	1.39	-3.80	1.06E-03	4.90E-02	-0.78
Uxt	-1.31	3.79	1.09E-03	4.98E-02	-0.80
Ddx3	-1.31	3.78	1.11E-03	5.04E-02	-0.82

*FC, adjusted fold change in expression, ratio of *Camk2n1*$^{-/-}$ to SHR; †Benjamini-Hochberg adjusted p-value. ‡Beta-coefficient.

Table S6. Significant module KEGG pathways and genes in LV associated with *Camk2n1*

KEGG pathway	Significant pathway genes
Steelblue	
Cell cycle	*Cdc20, Ccna2, Ccne1, Cdk1, Bub1b, Cdc25b*
Oocyte meiosis	*Cdc20, Ppp2r1b, Ccne1, Cdk1, Fbxo5*
Viral carcinogenesis	*Cdc20, Hist2h4a, Ccna2, Ccne1, Cdk1, Hist1h2bb*
darkturquoise	
Antigen processing and presentation	*Cd74, Psme2, Klrd1, Ifi30, B2m*
Black	
Thyroid hormone signaling pathway	*Kat2b, Med14, Pik3ca, Notch4, Slc16a10, Gata4, Atp1a1*
Regulation of actin cytoskeleton	*Fgf16, Pik3ca, Limk2, Pdgfc, Arpc5l, Myh9, Braf, Fgfr3, Dock1*
Blue	
AMPK signaling pathway	*Prkab2, Pfkfb4, Pdpk1, Tsc2, Hmgcr, Eef2, Adipor2, Elavl1, Mtor, Prkag3, Ppp2ca, Rab10, Creb3, Creb1, Rheb, Akt2, Akt1, Ulk1, Cd36*
Non-small cell lung cancer	*Rb1, Cdk6, Pdpk1, Akt2, Araf, Rarb, Akt1, Plcg1, Stk4, Tp53, Mapk3*
Turquoise	
Ribosome	*Rpl4, Mrps17, Mrps15, Rpl32, Mrps12, Rpl10l, Mrpl17, Mrpl15, Mrpl12, Rpl10a, Rpl6, Mrpl11, Mrpl33, Rps4x, Rpl7a, Mrpl2, Rps16, Rpl13, Rplp2, Rpl38, Rpl15, Mrpl9, Rpl37, Rpl18, Rps11, Rps27a, Rpl41, Rpl36a-Hnrnph2, Rpl22, Mrpl27, Mrpl28, Mrps21, Mrpl24, Rpl3l, Mrpl21, Mrps18c, Rps28, Rpl27, Rps21, Rsl24d1*
Spliceosome	*Sf3b5, Sf3b2, Sf3b3, Eif4a3, Usp39, Prpf8, Zmat2, Snrpd1, Dhx38, Snrpd3, Hnrnpa1, Sf3b1, Srsf10, Ctnnbl1, Sf3a1, Hnrnpa3, Hspa1l, Ccdc12, Alyref, Bud31, Thoc1, Thoc3, Crnkl1, Lsm3, Cdc40, Cherp, Lsm8, Prpf4, Snrnp40, Phf5a, Ddx39b, Syf2, Prpf3, Acin1, Ppih, Srsf5, Snrpa, Snrpb, Rbm22*
Oxidative phosphorylation	*Ndufb9, Cox7b, Ndufb8, Ndufa13, Ndufb10, Uqcrb, Ndufa11, Ndufb11, Ndufb5, Ndufa12, Cox17, Cox4i2, Ndufb3, Ndufb2, Cox7a2, Atp5g3, Cox6a1, Atp5o, Atp5g2, Cox5b, Cox7c, Ndufv3, Atp6v1d, Atp6v1c1, Atp6v1f, Ndufa9, Ndufa8, Ndufa7, Ndufa6, Ndufa4, Ndufa3, Ndufc1, Cox6c, Cox7a2l, Ndufs8, Uqcrc1, Ndufs3, Atp6v0d1*
Alzheimer's disease	*Ndufb9, Cox7b, Ndufb8, Ndufa13, Ndufb10, Uqcrb, Ndufa11, Ndufb11, Ndufb5, Ndufa12, Cox4i2, Ndufb3, Ndufb2, Itpr1, Itpr2, Cox7a2, Ide, Atp5g3, Cox6a1, Atp5o, Atp5g2, Cox5b, Cox7c, Aph1a, Ppp3cc, Ndufv3, Apoe, Ndufa9, Ndufa8, Ndufa7, Ndufa6, Ndufa4, Ndufa3, Ndufc1, Cox6c, Ern1, Cox7a2l, Ndufs8, Uqcrc1, Ndufs3, Mapt, Calm1*
Non-alcoholic fatty liver disease	*Ndufb9, Cox7b, Ndufb8, Ndufa13, Ndufb10, Uqcrb, Ndufa11, Ndufb11, Ndufb5, Ndufa12, Cox4i2, Ndufb3, Ndufb2, Cox7a2, Cox6a1, Cox5b, Cox7c, Cdc42, Rxra, Akt3, Ndufv3, Il6r, Map3k5, Ndufa9, Ndufa8, Ndufa7, Ndufa6, Ndufa4, Ndufa3, Traf2, Ndufc1, Cox6c, Ern1, Cox7a2l, Ndufs8, Ddit3, Uqcrc1, Ndufs3, Ppara*

Parkinson's disease	*Ndufb9, Cox7b, Ndufb8, Ndufa13, Ndufb10, Uqcrb, Ndufa11, Ndufb11, Ndufb5, Ndufa12, Cox4i2, Ndufb3, Ndufb2, Cox7a2, Atp5g3, Cox6a1, Ube2j2, Atp5o, Atp5g2, Cox5b, Cox7c, Ndufv3, Ndufa9, Ndufa8, Ndufa7, Ndufa6, Ndufa4, Ndufa3, Ndufc1, Ube2g2, Cox6c, Cox7a2l, Ndufs8, Adora2a, Uqcrc1, Ndufs3*
Huntington's disease	*Ndufb9, Cox7b, Ndufa13, Ndufb8, Hdac2, Ndufa11, Ndufb10, Uqcrb, Ndufa12, Ndufb11, Ndufb5, Cox4i2, Ndufb3, Ndufb2, Itpr1, Clta, Htt, Cox7a2, Atp5g3, Cox5b, Cox6a1, Atp5o, Atp5g2, Cox7c, Polr2d, Polr2e, Ndufv3, Polr2l, Ndufa9, Ndufa8, Ndufa7, Gpx1, Tbp, Ndufa6, Ndufa4, Ndufa3, Ndufc1, Cox6c, Cox7a2l, Ndufs8, Uqcrc1, Ndufs3, Tbpl1*
Metabolic pathways	*Pank4, Cda, Ndufa13, Dgkd, Ndufa11, Galnt16, Ndufa12, Uxs1, Dgka, Galnt18, Xylt2, Cox6a1, Nadsyn1, Ndst2, Urod, Fpgs, Dgat1, Cmbl, Dgkz, Dpm1, Acly, Pla2g16, Polg2, Dgkq, Mthfd2l, Uqcrc1, Atp6v0d1, Mtmr3, Nnmt, Uqcrb, Ndufb10, Maob, Ndufb11, Maoa, Cox17, Uap1, Ak4, Pla2g5, Pla2g6, Atp5o, Ak6, Mtmr6, Abo, Mtm1, Hlcs, Rdh10, Afmid, Pip5k1a, St3gal5, Inpp5k, B4galnt1, Ndufv3, Fuk, Atp6v1d, Atp6v1c1, St3gal2, Pck2, Atp6v1f, Tgds, Pmm2, Coq7, Coq5, Qdpr, Ext2, Coq3, Pi4ka, Ppt1, Pemt, Cryl1, Pigu, Cox7b, Hexb, Cox4i2, Apip, Gpt, Pygl, Atp5g3, Atp5g2, Cox7c, Smpd2, Atic, Sptlc1, Spr, Nmrk2, Pgm2, Upp1, Enoph1, Polg, Acad8, Pla2g12a, Sphk2, Pgam2, Akr1a1, Pla2g4a, Nme3, Ndufc1, Itpkb, Itpkc, Ugdh, Ndufs8, Piga, Ehhadh, Agps, Ndufs3, Rgn, B3gnt2, Pik3c3, Pigf, Dctpp1, B4galt7, B4galt4, Bcat2, Prps2, Ndufb9, Pdxk, Ndufb8, Acss3, Dtymk, Ahcy, Mvk, Galt, Acss2, Sgms1, Ndufb5, Prim1, Ndufb3, Lama3, Ndufb2, Cox5b, Ppox, Ltc4s, Pts, Pold3, Hmgcl, Pold4, Mat2a, Mthfd1l, Polr2d, Polr2e, Bpnt1, Cept1, Polr2l, Cbr3, Ndufa9, Ndufa8, Ndufa7, Ndufa6, Ndufa4, Tpk1, B3gat3, Ndufa3, Nmnat3, Cox6c, Dhodh, Gstz1, Galc, Gale, Chpt1, Plcd3, Lpin2*
Proteasome	*Psmb10, Psmb9, Psmb6, Psmd8, Psma6, Psmb7, Psmd6, Psmc6, Psmb5, Psmb2, Psmd4, Psma2, Psmb1, Psme1, Psmd1, Psmf1*
Ubiquitin mediated proteolysis	*Ube2d3, Ube2d1, Klhl13, Rchy1, Ube2j2, Cbl, Ube2q1, Ube2q2, Btrc, Ube2i, Ube2b, Smurf1, Ube2e3, Huwe1, Ube2e1, Wwp2, Ube2g2, Rbx1, Ube2s, Nedd4, Ube2n, Uba2, Anapc5, Stub1, Ube2m*
Protein processing in endoplasmic reticulum	*Ube2d3, Ube2d1, Ube2j2, Herpud1, Sec61a2, Erlec1, Hsph1, Sec61g, Sil1, Sec23b, Sec31b, Map3k5, Pdia3, Bcap31, Sec24a, Hspa1l, Amfr, Ssr2, Ube2e3, Ube2e1, Traf2, Ube2g2, Rbx1, Ern1, Dnajc3, Ddit3, Stub1, P4hb, Nfe2l2*
Peroxisome	*Pex16, Pecr, Abcd2, Mvk, Ech1, Mpv17l, Pex2, Hmgcl, Nudt7, Prdx5, Pex6, Ehhadh, Pxmp2, Agps, Hacl1, Crat, Paox*
Fc gamma R-mediated phagocytosis	*Syk, Sphk2, Prkce, Prkcd, Arpc5l, Pla2g4a, Asap2, Pla2g6, Cdc42, Hck, Ptprc, Arpc2, Arpc3, Akt3, Rps6kb2, Pip5k1a, Raf1, Wasf2*
Phosphatidylinositol signaling system	*Mtmr3, Dgkd, Dgka, Itpr1, Itpr2, Dgkz, Mtmr6, Mtm1, Itpkb, Itpkc, Dgkq, Pi4ka, Inpp5k, Pip5k1a, Pik3c3, Calm1, Plcd3, Ip6k1*

Table S7. Weighted gene co-expression network analysis module KEGG pathways and GO terms most significantly correlated with *Camk2n1* in LV

LV KEGG pathways (*P*-value)	LV GO terms (*P*-value)
Steelblue	
Cell cycle (3.47e-03)<br>Oocyte meiosis (1.73e-02)<br>Viral carcinogenesis (1.77e-02)	*Molecular function*:<br>microtubule motor activity (2.87e-05)<br>*Cellular component:*<br>kinetochore (4.04e-10)<br>*Biological process:*<br>mitotic cell cycle (2.17e-10)
Darkturquoise	
Antigen processing and presentation (6.05e-03)	*Molecular function*:<br>RNA binding (1.76e-03)<br>*Cellular component*:<br>nucleolus (2.82e-03)<br>*Biological process*:<br>RNA secondary structure unwinding (2.46e-03)
Black	
Thyroid hormone signaling pathway (2.23e-02)	*Cellular composition*:<br>autolysosome (8.18e-03)
Blue	
Regulation of actin cytoskeleton (2.23e-02)<br>AMPK signaling pathway (7.42e-03)<br>Non-small cell lung cancer (1.90e-02)	*Molecular function*:<br>RNA binding (7.03e-09)<br>*Cellular component:*<br>nucleolus (9.35e-05)<br>*Biological process:*<br>positive regulation of peptidyl-serine phosphorylation (3.29e-04)
Turquoise	
Ribosome (1.37e-09)<br>Spliceosome (1.41e-09)<br>Oxidative phosphorylation (3.18e-09)<br>Alzheimer's disease (2.37e-08)<br>Non-alcoholic fatty liver disease (2.88e-08)<br>Parkinson's disease (1.89e-07)<br>Huntington's disease (3.72e-07)<br>Metabolic pathways (1.16e-05)<br>Proteasome (1.42e-05)<br>Ubiquitin mediated proteolysis (9.16e-03)<br>Protein processing in endoplasmic reticulum (9.16e-03)<br>Peroxisome (1.80e-02)<br>Fc gamma R-mediated phagocytosis (2.37e-02)<br>Phosphatidylinositol signaling system (4.1e-02)	*Molecular function*:<br>RNA binding (2.18e-15)<br>*Cellular component:*<br>mitochondrial inner membrane (1.49e-12)<br>*Biological process:*<br>translation (2.60e-08)

Table S8. Significantly differentially expressed genes in *Camk2n1*$^{-/-}$ vs SHR EAT

Gene ID	FC*	t	Raw P-value	P_{adj} †	β‡
Pcdh7	-2.08	7.38	2.96E-07	3.35E-03	6.62
Pla2g5	-2.35	7.04	6.00E-07	3.35E-03	6.00
Ptpn13	-1.83	6.96	7.11E-07	3.35E-03	5.86
Parpbp	-2.09	6.79	1.03E-06	3.35E-03	5.53
Ncapg	-2.33	6.76	1.10E-06	3.35E-03	5.48
Pla2g2a	-6.46	6.75	1.13E-06	3.35E-03	5.45
Slc39a8	-2.27	6.49	2.01E-06	5.10E-03	4.94
Rbfox3	-2.04	6.36	2.64E-06	5.87E-03	4.70
Arhgap8	-1.73	6.17	4.08E-06	7.25E-03	4.31
Cldn15	-3.20	6.17	4.08E-06	7.25E-03	4.31
Id3	2.02	-6.13	4.49E-06	7.25E-03	4.23
Serpina3n	5.41	-6.06	5.19E-06	7.69E-03	4.10
Gcnt2	-2.05	5.81	9.24E-06	1.12E-02	3.58
Rcbtb1	1.69	-5.79	9.61E-06	1.12E-02	3.55
Mx2	-2.37	5.78	9.90E-06	1.12E-02	3.52
Kntc1	-1.92	5.75	1.06E-05	1.12E-02	3.46
Lama2	1.42	-5.74	1.07E-05	1.12E-02	3.45
Sdc4	-1.70	5.70	1.18E-05	1.16E-02	3.37
Dsg2	-2.20	5.68	1.24E-05	1.16E-02	3.32
Slc26a3	-2.71	5.66	1.30E-05	1.16E-02	3.27
Klf5	-1.97	5.60	1.47E-05	1.25E-02	3.16
Aurkb	-1.91	5.46	2.03E-05	1.57E-02	2.87
Lmna	-1.41	5.44	2.17E-05	1.57E-02	2.82
Gata5	-2.12	5.42	2.24E-05	1.57E-02	2.78
Prodh	1.78	-5.42	2.25E-05	1.57E-02	2.78
Fbxo5	-1.63	5.41	2.30E-05	1.57E-02	2.76
Pkhd1l1	-3.10	5.37	2.51E-05	1.65E-02	2.68
Cdh11	-1.97	5.34	2.69E-05	1.68E-02	2.62
Msln	-3.10	5.34	2.74E-05	1.68E-02	2.60
Psip1	-1.58	5.31	2.89E-05	1.71E-02	2.56
Oasl2	-2.07	5.26	3.24E-05	1.81E-02	2.45
Casc5	-1.81	5.26	3.30E-05	1.81E-02	2.44
LOC100910934	-2.04	5.25	3.37E-05	1.81E-02	2.42
Wfdc21	-5.76	5.21	3.68E-05	1.89E-02	2.34
Upk1b	-3.99	5.21	3.72E-05	1.89E-02	2.33
Ccp110	-1.64	5.18	3.96E-05	1.93E-02	2.27
Baiap2l1	-2.28	5.17	4.05E-05	1.93E-02	2.25
Parp12	-1.47	5.16	4.17E-05	1.93E-02	2.22
Ccl21	1.91	-5.14	4.38E-05	1.93E-02	2.18
Gpd2	1.81	-5.13	4.42E-05	1.93E-02	2.17
Tpx2	-2.08	5.13	4.47E-05	1.93E-02	2.16

Paqr6	1.59	-5.11	4.64E-05	1.93E-02	2.13
Prr5l	-1.73	5.11	4.67E-05	1.93E-02	2.12
Nt5dc2	-1.46	5.09	4.89E-05	1.93E-02	2.08
AABR07059632.1	-1.92	5.08	4.94E-05	1.93E-02	2.07
Csf1r	1.54	-5.08	5.00E-05	1.93E-02	2.06
Cdkn1a	1.88	-5.07	5.16E-05	1.95E-02	2.03
Gtpbp2	-1.60	5.06	5.26E-05	1.95E-02	2.01
Slfn3	-2.28	5.04	5.49E-05	1.95E-02	1.98
Emp1	-1.43	5.04	5.49E-05	1.95E-02	1.98
Colgalt2	-1.73	5.01	5.91E-05	2.03E-02	1.91
5SrRNA	-2.45	5.01	5.94E-05	2.03E-02	1.90
Fras1	-1.99	4.96	6.64E-05	2.17E-02	1.80
Mki67	-2.08	4.95	6.70E-05	2.17E-02	1.79
Slfn13	-1.65	4.93	7.07E-05	2.24E-02	1.75
S100a6	-1.46	4.92	7.26E-05	2.26E-02	1.72
Ska2	-1.65	4.91	7.46E-05	2.28E-02	1.70
Cobl	-1.64	4.89	7.83E-05	2.36E-02	1.65
Myo1d	-1.50	4.87	8.17E-05	2.42E-02	1.61
C2cd2	-1.40	4.86	8.38E-05	2.42E-02	1.59
C1s	-1.46	4.86	8.46E-05	2.42E-02	1.58
Fgf9	-2.33	4.84	8.77E-05	2.47E-02	1.55
Ncapd2	-1.49	4.81	9.40E-05	2.61E-02	1.49
Dpep1	1.69	-4.80	9.60E-05	2.61E-02	1.47
Uchl3	-1.42	4.80	9.70E-05	2.61E-02	1.46
Ect2	-1.95	4.78	1.02E-04	2.69E-02	1.42
Enpp5	1.44	-4.75	1.09E-04	2.83E-02	1.35
Cdc37	-1.70	4.75	1.10E-04	2.83E-02	1.35
Vnn1	-1.61	4.73	1.14E-04	2.86E-02	1.32
Hs3st1	-1.81	4.73	1.14E-04	2.86E-02	1.31
Asf1b	-1.94	4.71	1.19E-04	2.92E-02	1.27
Fmo2	1.97	-4.71	1.20E-04	2.92E-02	1.26
Kif15	-1.73	4.68	1.28E-04	2.99E-02	1.21
Fbxl8	-1.45	4.66	1.33E-04	2.99E-02	1.17
Aspm	-2.06	4.66	1.34E-04	2.99E-02	1.16
Kif20a	-1.87	4.66	1.35E-04	2.99E-02	1.16
Zfp697	-1.76	4.66	1.36E-04	2.99E-02	1.15
Clic3	-2.00	4.65	1.38E-04	2.99E-02	1.14
Diaph3	-2.29	4.65	1.39E-04	2.99E-02	1.13
Ddah1	-1.97	4.65	1.39E-04	2.99E-02	1.13
Plk1	-1.72	4.64	1.42E-04	2.99E-02	1.11
Mical2	-1.63	4.63	1.43E-04	2.99E-02	1.10
Plk4	-1.90	4.63	1.44E-04	2.99E-02	1.10
Lrrn4	-3.74	4.63	1.44E-04	2.99E-02	1.10

Dlgap5	-1.46	4.62	1.47E-04	2.99E-02	1.08
Ttc9c	-1.48	4.62	1.47E-04	2.99E-02	1.08
Rtp4	-1.83	4.62	1.48E-04	2.99E-02	1.07
Stmn1	-1.99	4.61	1.52E-04	3.02E-02	1.05
Upk3b	-2.99	4.61	1.53E-04	3.02E-02	1.04
Hey1	1.58	-4.60	1.56E-04	3.05E-02	1.03
Lsr	-1.73	4.59	1.58E-04	3.06E-02	1.01
Gpm6a	-2.29	4.58	1.63E-04	3.11E-02	0.99
Tube1	-1.58	4.58	1.64E-04	3.11E-02	0.98
Iqgap3	-1.95	4.53	1.83E-04	3.42E-02	0.88
Prmt1	-1.77	4.51	1.93E-04	3.56E-02	0.83
Crim1	-1.39	4.51	1.94E-04	3.56E-02	0.83
P4ha3	-2.75	4.50	1.97E-04	3.56E-02	0.81
Rnf144b	-1.62	4.50	1.98E-04	3.56E-02	0.81
Pdk3	-1.46	4.49	2.04E-04	3.63E-02	0.78
Arhgap11a	-1.67	4.47	2.12E-04	3.73E-02	0.75
Lrrc1	-1.95	4.47	2.14E-04	3.74E-02	0.74
Usp18	-2.37	4.46	2.19E-04	3.76E-02	0.72
Ndufs8	-1.43	4.45	2.20E-04	3.76E-02	0.71
Nebl	1.56	-4.43	2.31E-04	3.89E-02	0.67
Rbl1	-1.51	4.43	2.32E-04	3.89E-02	0.67
Hbb	1.84	-4.43	2.36E-04	3.91E-02	0.65
LOC100912517	-1.82	4.41	2.43E-04	3.99E-02	0.62
NA	2.89	-4.40	2.54E-04	4.13E-02	0.58
Camk2n1[§]	-0.65	-4.39	2.60E-04	4.13E-02	0.56
NA	2.42	-4.38	2.62E-04	4.13E-02	0.55
Gpnmb	1.69	-4.38	2.63E-04	4.13E-02	0.55
C4	-1.85	4.38	2.63E-04	4.13E-02	0.55
Irgm	-1.63	4.36	2.77E-04	4.26E-02	0.50
LOC498265	-1.52	4.36	2.77E-04	4.26E-02	0.50
Ninj2	1.55	-4.36	2.78E-04	4.26E-02	0.50
Rtn4r	-1.69	4.35	2.85E-04	4.31E-02	0.48
Pcna	-1.47	4.34	2.86E-04	4.31E-02	0.47
Hist1h4m	-1.54	4.34	2.89E-04	4.31E-02	0.47
Ska3	-1.88	4.30	3.17E-04	4.69E-02	0.38
Bst2	-1.57	4.30	3.22E-04	4.71E-02	0.37
Cdk1	-1.92	4.29	3.24E-04	4.71E-02	0.36
NA	2.10	-4.29	3.26E-04	4.71E-02	0.35
Kpna2	-1.46	4.29	3.29E-04	4.71E-02	0.35
RGD1307704	-1.37	4.27	3.38E-04	4.77E-02	0.32
Top2a	-1.92	4.27	3.39E-04	4.77E-02	0.32
Rcan1	-1.45	4.25	3.59E-04	5.01E-02	0.27
Mmp2	1.30	-4.25	3.61E-04	5.01E-02	0.26

Prim1	-1.79	4.24	3.64E-04	5.02E-02	0.25
Klf3	1.32	-4.24	3.69E-04	5.04E-02	0.24

*FC, adjusted fold change in expression, ratio of *Camk2n1*$^{-/-}$ to SHR.
†Benjamini-Hochberg adjusted p-value.
‡β-coefficient.
§Probe sets complementary to exon1 (upstream of the 38bp deletion) of *Camk2n1*, detected transcripts and showed downregulation in the *Camk2n1*$^{-/-}$ rats. These data most likely residual transcription upstream of the deletion and not evidence of full-length transcript, as we showed the presence of the 38bp deletion in *Camk2n1* genomic DNA, which introduces a premature stop codon 12 codons after the deletion (Fig. S1A). We further show the presence of the 38bp deletion in cDNA, and the absence of Camk2n1 protein confirming the knockout (Fig. 1A-C).

Table S9. Significant module KEGG pathways and genes in EAT associated with *Camk2n1*

KEGG_2016	Pathway genes
Turquoise	
Cell cycle	*Pcna, Mcm7, Bub1b, Cdc20, Fzr1, Ccnb1, E2f1, Bub1, Ywhah, Plk1, Cdc25b, Rbx1, Ccna2, Rbl2, Wee1, Rbl1, Cdk6, Ccne2, Cdk1, Mcm4, Mcm5, Atm, Anapc1, Mad2l1, Atr*
DNA replication	*Rfc5, Pola1, Pold4, Pcna, Mcm7, Rfc2, Prim1, Pold1, Rpa1, Mcm4, Mcm5*
Mismatch repair	*Rfc5, Pold4, Pcna, Rfc2, Pold1, Rpa1*
Sienna3	
Apoptosis	*Spta1, Tmem127, Dnajc11, Ctsv, Trib2, Mpzl2*
Tan	
Complement and coagulation cascades	*C1s, C1qb, Vtn, Thbd, F8, C4, C5ar1, C1qc*
Darkgreen	
Leishmaniasis	*Hla-Dma, Itgb2, Hla-Dra, Fos, Rela*
HTLV-I infection	*Egr1, Hla-Dma, Cdc26, Itgb2, Hla-Dra, Adcy1, Fos, Ran, Rela*
Antigen processing and presentation	*Cd74, Cd4, Hla-Dma, Hla-Dra, Klrd1*
Greenyellow	
Influenza A	*Ifih1, Cxcl10, Rsad2, Oas1, Ddx58, Oas2, Stat1, Stat2, Mx1, Irf7, Irf9, Tlr3*
Hepatitis B	*Oas1, Bad, Ddx58, Oas2, Stat1, Stat2, Ifit1b, Irf7, Irf9, Tlr3*
Hepatitis C	*Ifih1, Oas1, Ddx58, Oas2, Stat1, Stat2, Ifit1b, Irf7, Tap1, Irf9, Tlr3*
Herpes simplex infection	*Ifih1, Oas1, Ddx58, Oas2, Stat1, Stat2, Mx1, Irf7, Irf9*
Measles	*Ifih1, Cxcl10, Ddx58, Irf7, Isg15*
RIG-I-like receptor signaling pathway	*Ifih1, Bad, Ddx58, Stat1, Stat2, Irf7, Tlr3*
RNA degradation	*Exosc6, Ttc37, Lsm4, Skiv2l2, Edc3*

Table S10. Weighted gene co-expression network analysis module KEGG pathways and GO terms most significantly correlated with *Camk2n1* in EAT

EAT KEGG pathways (*P*-value)	EAT GO terms (*P*-value)
Turquoise	
Cell cycle (3.17e-08)<br>DNA replication (2.94e-05)<br>Mismatch repair (3.11e-02)	*Molecular function*:<br>RNA binding (1.23e-04)<br>*Cellular component:*<br>condensin complex (3.28e-05)<br>*Biological process:*<br>sister chromatid cohesion (5.88e-10)
Sienna3	
Apoptosis (3.80e-03)	none
Tan	
Complement and coagulation cascades (1.41e-02)	*Biological process*:<br>ketone body biosynthetic process (2.30e-02)
Darkgreen	
Leishmaniasis (3.91e-02)<br>HTLV-I infection (3.91e-02)<br>Antigen processing and presentation (3.92e-02)	*Molecular function:*<br>MHC class II protein complex binding (4.54e-02)<br>*Cellular component*:<br>integral component of plasma membrane (5.16e-04)
Greenyellow	
Influenza A (8.23e-05)<br>Hepatitis B (2.93e-02)<br>Hepatitis C (1.61e-04)<br>Herpes simplex infection (3.27e-04)<br>Measles (7.39e-04)<br>RIG-I-like receptor signaling pathway (2.93e-02)<br>RNA degradation (3.43e-02)	*Molecular function:*<br>double-stranded RNA binding (3.54e-03)<br>*Biological process*:<br>type I interferon signaling pathway (2.69e-12)

Table S11. GTEx *cis*-eQTLs for *CAMK2N1* in relevant cardio-metabolic tissues.

Variant Id	SNP Id	*P*-value	Effect Size
Adipose - Subcutaneous			
1_20538163_C_T_b37	rs4654840	3.60e-09	-0.31
1_20524056_C_T_b37	rs10799613	3.30e-08	0.30
1_20543974_T_G_b37	rs6426621	4.20e-08	-0.29
1_20527064_A_G_b37	rs6691280	4.50e-08	0.30
1_20539175_C_A_b37	rs11807163	7.10e-08	-0.29
1_20523601_C_T_b37	rs12129129	7.70e-08	0.30
1_20537226_C_T_b37	rs10916721	8.00e-08	-0.28
1_20544835_A_G_b37	rs2213801	9.40e-08	-0.29
1_20550123_A_T_b37	rs6685397	1.10e-07	-0.29
1_20544451_G_A_b37	rs3921122	1.10e-07	-0.29
1_20510681_T_C_b37	rs12409350	1.20e-07	0.30
1_20539100_C_T_b37	rs10916722	1.70e-07	-0.28
1_20539102_T_C_b37	rs10916723	1.70e-07	-0.28
1_20526237_A_G_b37	rs10753484	1.70e-07	0.30
1_20541999_G_C_b37	rs6687047	1.80e-07	-0.28
1_20528256_C_G_b37	rs6426619	2.00e-07	0.30
1_20520732_T_C_b37	rs7542020	2.90e-07	0.29
1_20530208_A_G_b37	rs3820320	3.10e-07	0.29
1_20542393_A_G_b37	rs10737446	3.50e-07	-0.27
1_20513040_T_A_b37	rs7535938	3.60e-07	0.28
1_20539523_C_A_b37	rs35862517	3.70e-07	-0.27
1_20526047_G_A_b37	rs6668468	3.80e-07	0.29
1_20543744_T_G_b37	rs7536516	4.70e-07	-0.27
1_20511808_C_G_b37	rs6426617	5.00e-07	0.28
1_20524374_T_A_b37	rs10916720	8.10e-07	0.29
1_20550472_A_T_b37	rs10916725	1.00e-06	-0.26
1_20550899_A_G_b37	rs56340248	1.00e-06	-0.26
1_20539853_A_C_b37	rs10737445	1.10e-06	-0.26
1_20550372_C_T_b37	rs10916724	1.10e-06	-0.27
1_20540557_A_G_b37	rs2213800	1.10e-06	-0.26
1_20545070_T_C_b37	rs10799616	1.20e-06	-0.26
1_20546241_G_GAGA_b37	rs34864541	1.30e-06	-0.26
1_20542730_C_G_b37	rs739274	1.40e-06	-0.25
1_20541370_A_G_b37	rs10799615	1.60e-06	-0.24
1_20510752_T_C_b37	rs12141257	1.80e-06	0.28
1_20510755_T_C_b37	rs12141258	1.80e-06	0.28
1_20531422_T_C_b37	rs6659296	1.90e-06	0.24
1_20543486_A_G_b37	rs7533911	1.90e-06	-0.26
1_20540099_A_AT_b37	rs397814723	2.30e-06	-0.26
1_20540724_G_A_b37	rs2227227	2.60e-06	-0.25

1_20514018_G_A_b37	rs10157172	4.20e-06	0.27
1_20513999_G_GC_b37	rs386366405	4.20e-06	0.27
1_20539637_C_A_b37	rs10799614	6.70e-06	0.40
1_20519808_G_A_b37	rs1061588	7.50e-06	0.26
1_20533678_A_C_b37	rs719683	8.80e-06	0.23
1_20478448_A_G_b37	rs61768664	1.60e-05	0.45
1_20624063_C_T_b37	rs10916754	1.80e-05	0.24
1_20555321_G_C_b37	rs34668419	2.10e-05	-0.20
1_20732396_G_T_b37	rs9661160	2.90e-05	-0.23
1_20618450_G_A_b37	rs1028298	3.30e-05	0.20
1_20732475_A_G_b37	rs9660217	3.50e-05	-0.23
1_21513911_A_G_b37	rs72654867	3.80e-05	0.42
Adipose - Visceral			
1_20538163_C_T_b37	rs4654840	1.90e-15	-0.43
1_20539523_C_A_b37	rs35862517	2.20e-15	-0.43
1_20539100_C_T_b37	rs10916722	4.00e-15	-0.43
1_20539102_T_C_b37	rs10916723	4.00e-15	-0.43
1_20544835_A_G_b37	rs2213801	5.60e-15	-0.43
1_20540724_G_A_b37	rs2227227	6.10e-15	-0.41
1_20542393_A_G_b37	rs10737446	6.80e-15	-0.42
1_20543974_T_G_b37	rs6426621	6.90e-15	-0.42
1_20539175_C_A_b37	rs11807163	1.10e-14	-0.42
1_20539853_A_C_b37	rs10737445	1.30e-14	-0.42
1_20542730_C_G_b37	rs739274	1.30e-14	-0.41
1_20537226_C_T_b37	rs10916721	1.40e-14	-0.41
1_20544451_G_A_b37	rs3921122	1.80e-14	-0.43
1_20540557_A_G_b37	rs2213800	1.80e-14	-0.42
1_20540099_A_AT_b37	rs397814723	3.40e-14	-0.42
1_20550123_A_T_b37	rs6685397	3.60e-14	-0.42
1_20545070_T_C_b37	rs10799616	3.80e-14	-0.41
1_20543744_T_G_b37	rs7536516	4.90e-14	-0.41
1_20541999_G_C_b37	rs6687047	7.20e-14	-0.40
1_20546241_G_GAGA_b37	rs34864541	9.70e-14	-0.41
1_20550472_A_T_b37	rs10916725	5.20e-13	-0.39
1_20550372_C_T_b37	rs10916724	6.20e-13	-0.40
1_20550899_A_G_b37	rs56340248	7.80e-13	-0.39
1_20541370_A_G_b37	rs10799615	9.70e-13	-0.37
1_20543486_A_G_b37	rs7533911	1.80e-12	-0.40
1_20533678_A_C_b37	rs719683	6.50e-11	0.32
1_20552485_A_G_b37	rs35015448	4.60e-09	-0.29
1_20519808_G_A_b37	rs1061588	4.80e-09	0.35
1_20552612_A_T_b37	rs12725354	4.90e-09	-0.29
1_20531422_T_C_b37	rs6659296	5.50e-09	0.29

1_20551745_C_T_b37	rs2097532	5.90e-09	-0.29
1_20530208_A_G_b37	rs3820320	8.80e-09	0.34
1_20524374_T_A_b37	rs10916720	1.10e-08	0.35
1_20514018_G_A_b37	rs10157172	1.90e-08	0.33
1_20528256_C_G_b37	rs6426619	2.50e-08	0.33
1_20513999_G_GC_b37	rs386366405	2.50e-08	0.33
1_20552319_T_TG_b37	rs397686962	2.80e-08	-0.27
1_20510752_T_C_b37	rs12141257	3.40e-08	0.33
1_20510755_T_C_b37	rs12141258	3.40e-08	0.33
1_20526237_A_G_b37	rs10753484	3.50e-08	0.33
1_20555321_G_C_b37	rs34668419	5.20e-08	-0.26
1_20554964_CG_C_b37	rs397979475	7.40e-08	-0.26
1_20526047_G_A_b37	rs6668468	8.90e-08	0.32
1_20520732_T_C_b37	rs7542020	3.80e-07	0.29
1_20527064_A_G_b37	rs6691280	4.30e-07	0.30
1_20523601_C_T_b37	rs12129129	9.90e-07	0.28
1_20510681_T_C_b37	rs12409350	1.20e-06	0.29
1_20513040_T_A_b37	rs7535938	1.30e-06	0.28
1_20511808_C_G_b37	rs6426617	2.40e-06	0.27
1_20524056_C_T_b37	rs10799613	5.70e-06	0.26
1_20539637_C_A_b37	rs10799614	1.90e-05	0.40
Artery - Aorta			
1_20834610_C_A_b37	rs6700034	1.70e-07	-0.45
1_20857630_G_A_b37	rs2000366	1.20e-05	-0.26
1_20849041_G_GC_b37	rs397979482	1.60e-05	-0.34
1_20798620_A_G_b37	rs12724366	1.90e-05	-0.34
1_20786097_G_T_b37	rs12760079	2.10e-05	-0.33
1_20788642_T_A_b37	rs12729605	2.10e-05	-0.33
1_20876920_C_T_b37	rs12040369	2.70e-05	-0.22
Artery - Tibial			
1_20834610_C_A_b37	rs6700034	2.60e-07	-0.32
1_20828900_G_C_b37	rs7512681	6.70e-07	-0.53
1_20783462_G_A_b37	rs12744385	2.30e-06	-0.26
1_20783494_T_G_b37	rs12730929	2.30e-06	-0.26
1_20783398_A_C_b37	rs12726785	2.60e-06	-0.26
1_20782983_G_C_b37	rs12743605	4.60e-06	-0.25
1_20782019_G_A_b37	rs12738179	5.20e-06	-0.27
1_20782750_T_G_b37	rs12729829	5.20e-06	-0.27
1_20695335_T_G_b37	rs12140218	5.60e-06	-0.29
1_20786097_G_T_b37	rs12760079	6.70e-06	-0.26
1_20788642_T_A_b37	rs12729605	6.70e-06	-0.26
1_20773908_G_A_b37	rs12723377	6.80e-06	-0.26
1_20798620_A_G_b37	rs12724366	7.30e-06	-0.26

1_20792736_G_GC_b37	rs397979478	1.30e-05	-0.26
Brain – Cerebellar Hemisphere			
1_20586380_A_G_b37	rs2187918	1.00e-06	-0.42
1_20586476_A_G_b37	rs1009942	1.00e-06	-0.42
1_20594390_T_TC_b37	rs35479874	1.20e-05	-0.39
Brain – Substantia nigra			
1_20775499_C_T_b37	rs112322975	5.60e-08	-0.78
1_20777021_G_A_b37	rs74335351	5.60e-08	-0.78
1_20786824_T_C_b37	rs112662287	5.60e-08	-0.78
1_20790806_C_T_b37	rs77167727	5.60e-08	-0.78
1_20796219_T_G_b37	rs112660177	5.60e-08	-0.78
1_20807073_G_T_b37	rs75810319	5.60e-08	-0.78
1_20869460_CT_C_b37	rs242	7.50e-08	-0.79
1_20846625_G_C_b37	rs112684754	1.40e-07	-0.75
1_20853036_G_A_b37	rs77547636	1.40e-07	-0.75
1_20860147_G_C_b37	rs75492067	1.40e-07	-0.75
1_20826243_C_T_b37	rs1044332	1.40e-07	-0.75
1_20829303_A_C_b37	rs111270978	1.40e-07	-0.75
1_20830127_G_A_b37	rs17412284	1.40e-07	-0.75
1_20808794_TC_T_b37	rs150001621	4.90e-07	-0.75
1_20875676_G_T_b37	rs74701752	5.70e-07	-0.76
1_20811184_C_T_b37	rs3767235	6.60e-07	-0.73
1_20819606_C_A_b37	rs77855246	6.60e-07	-0.73
1_20871750_C_T_b37	rs111235962	7.40e-07	-0.75
1_20875459_T_C_b37	rs4615814	7.40e-07	-0.75
1_20883582_G_A_b37	rs76440167	7.40e-07	-0.75
1_20883608_C_T_b37	rs79582714	7.40e-07	-0.75
1_20976535_G_A_b37	rs17414302	1.00e-06	-0.77
1_20821180_C_A_b37	rs76480145	1.80e-06	-0.66
1_20824366_G_A_b37	rs6658554	2.20e-06	-0.63
1_20814444_T_C_b37	rs56127503	2.40e-06	-0.66
1_20816766_G_A_b37	rs56100950	2.40e-06	-0.66
1_20817385_C_T_b37	rs12085103	2.40e-06	-0.66
1_20818659_G_C_b37	rs10916805	2.40e-06	-0.66
1_20851834_T_C_b37	rs7544348	2.50e-06	-0.64
1_20855188_A_G_b37	rs10916807	2.50e-06	-0.64
1_20855542_T_C_b37	rs10916808	2.50e-06	-0.64
1_20855879_C_T_b37	rs10916809	2.50e-06	-0.64
1_20856845_T_C_b37	rs11811983	2.50e-06	-0.64
1_20856037_C_T_b37	rs10916810	2.50e-06	-0.64
1_20831301_T_C_b37	rs80274432	4.00e-06	-0.63
1_20840568_T_TG_b37	rs146249783	4.00e-06	-0.63
1_20844074_T_C_b37	rs12094563	4.00e-06	-0.63

1_20850250_A_G_b37	rs77280889	4.00e-06	-0.63
1_20857064_G_A_b37	rs6698887	4.00e-06	-0.63
1_20857361_T_A_b37	rs6690792	4.00e-06	-0.63
1_20857643_G_A_b37	rs77110732	4.00e-06	-0.63
1_20821534_A_C_b37	rs74720529	4.00e-06	-0.63
1_20821965_A_G_b37	rs12076947	4.00e-06	-0.63
1_20822128_A_AG_b37	rs71684681	4.00e-06	-0.63
1_20832674_T_G_b37	rs12566937	4.00e-06	-0.63
1_20834789_T_C_b37	rs6669244	4.00e-06	-0.63
1_20835407_A_C_b37	rs79759487	4.00e-06	-0.63
1_20835492_T_C_b37	rs17412396	4.00e-06	-0.63
1_20837289_C_G_b37	rs12057500	4.00e-06	-0.63
1_20837541_A_C_b37	rs17403898	4.00e-06	-0.63
1_20852207_C_T_b37	rs7530736	4.00e-06	-0.63
1_20851931_G_A_b37	rs7552144	5.00e-06	-0.63
Heart – Left ventricle			
1_20759659_A_G_b37	rs10753503	7.22e-06	-0.27
1_20759231_C_A_b37	rs10753502	1.36e-05	-0.27
1_20759403_T_C_b37	rs10737449	1.43e-05	-0.27
Muscle - Skeletal			
1_20652798_G_T_b37	rs4590651	2.80e-07	-0.27
1_20659684_A_G_b37	rs12118463	3.50e-07	-0.27
1_20658649_C_A_b37	rs1076623	7.20e-07	-0.26
1_20660452_A_G_b37	rs34905880	7.20e-07	-0.26
1_20660795_G_A_b37	rs6666063	7.20e-07	-0.26
1_20651884_A_G_b37	rs4655195	7.70e-07	-0.26
1_20651901_C_T_b37	rs4655196	7.70e-07	-0.26
1_20661782_C_T_b37	rs4654853	8.70e-07	-0.26
1_20661495_T_C_b37	rs4654852	9.00e-07	-0.26
1_20634398_G_A_b37	rs10916758	1.60e-06	-0.23
1_20635875_G_A_b37	rs35931442	2.60e-06	-0.23
1_20637043_C_T_b37	rs12726937	2.60e-06	-0.23
1_20759231_C_A_b37	rs10753502	3.10e-06	-0.22
1_20759403_T_C_b37	rs10737449	3.10e-06	-0.22
1_20629227_G_C_b37	rs12408023	4.00e-06	-0.22
1_20634049_C_A_b37	rs11577734	4.00e-06	-0.22
1_20634661_G_A_b37	rs12409834	4.00e-06	-0.22
1_20636496_C_T_b37	rs72647040	4.10e-06	-0.24
1_20759659_A_G_b37	rs10753503	5.10e-06	-0.21
1_20662142_T_C_b37	rs12752465	8.90e-06	-0.24
1_20665538_T_C_b37	rs12125512	9.40e-06	-0.23
1_20678011_C_T_b37	rs2005957	1.00e-05	-0.24
1_20662938_A_G_b37	rs10916769	1.10e-05	-0.23

1_20660861_C_T_b37	rs66819832	1.10e-05	-0.23
1_20632407_G_A_b37	rs4654849	1.20e-05	0.22
1_20632908_C_T_b37	rs10916756	1.20e-05	0.22
1_20617419_T_C_b37	rs12738826	1.30e-05	-0.21
1_20657887_C_T_b37	rs1883165	1.30e-05	-0.23
1_20675933_G_A_b37	rs4655202	1.40e-05	-0.24
1_20619629_G_A_b37	rs16823816	1.40e-05	-0.21
1_20756045_G_A_b37	rs12087853	1.50e-05	-0.32
1_20756613_C_T_b37	rs12057443	1.50e-05	-0.32
1_20633305_G_T_b37	rs4655192	1.60e-05	0.20
1_20681004_G_C_b37	rs2281001	1.60e-05	-0.24
1_20620323_C_T_b37	rs2223225	1.90e-05	-0.18
1_20743546_C_T_b37	rs10916793	2.00e-05	-0.19
1_20633933_G_A_b37	rs6676490	2.20e-05	0.20
1_20651001_G_C_b37	rs11800294	2.40e-05	0.20
1_20658089_A_G_b37	rs4329481	2.50e-05	0.20
1_20636038_G_T_b37	rs12749098	2.50e-05	-0.20
1_20703026_C_T_b37	rs10916777	2.70e-05	-0.22
1_20639855_A_G_b37	rs12139016	2.70e-05	-0.21
1_20656824_A_G_b37	rs2072752	3.00e-05	-0.23
1_20661229_G_A_b37	rs55766191	3.10e-05	0.24
1_20668375_A_T_b37	rs10916770	3.10e-05	-0.23
1_20702104_G_C_b37	rs11587845	3.40e-05	-0.22
1_20634736_G_GA_b37	rs11444121	3.50e-05	0.20
1_20634739_CAA_C_b37	rs10546611	3.50e-05	0.20
1_20697104_G_A_b37	rs12081725	3.90e-05	-0.22
1_20699724_G_T_b37	rs11586341	3.90e-05	-0.22
1_20667619_C_T_b37	rs4654855	4.00e-05	-0.23
1_20624063_C_T_b37	rs10916754	4.10e-05	0.21
1_20684504_C_G_b37	rs12048103	4.10e-05	0.24
1_20687285_C_T_b37	rs12045792	4.10e-05	0.24
1_20687755_C_T_b37	rs12046639	4.10e-05	0.24
1_20688352_C_T_b37	rs60573766	4.10e-05	0.24
1_20688361_C_T_b37	rs75372910	4.10e-05	0.24
1_20717763_C_A_b37	rs1111366	4.30e-05	-0.18
1_20700777_G_A_b37	rs11587262	4.40e-05	-0.22
1_20701277_C_T_b37	rs10159448	4.40e-05	-0.22
1_20701293_T_TG_b37	rs57510627	4.40e-05	-0.22
1_20701297_T_C_b37	rs59638783	4.40e-05	-0.22
1_20720439_A_G_b37	rs1925668	4.50e-05	-0.18
1_20702420_C_CG_b37	rs397962122	5.10e-05	-0.21
1_20647164_G_A_b37	rs10916764	5.40e-05	-0.21
1_20757820_T_C_b37	rs55761633	5.40e-05	-0.31

Thyroid			
1_20813488_G_T_b37	rs1253918	2.00e-13	-0.36
1_20814522_A_G_b37	rs1152984	2.60e-13	-0.38
1_20816568_T_C_b37	rs1144966	2.90e-13	-0.37
1_20818917_GT_G_b37	rs869094803	5.90e-13	-0.37
1_20818115_A_G_b37	rs1152985	1.50e-12	-0.36
1_20818935_C_T_b37	rs1152986	8.00e-11	-0.30
1_20815402_A_C_b37	rs6683578	7.80e-08	-0.23
1_20883503_A_G_b37	rs7545658	8.70e-08	0.26
1_20821828_C_T_b37	rs35864454	2.70e-07	0.46
1_20866699_C_T_b37	rs645548	5.10e-07	0.43
1_20883522_G_C_b37	rs7555911	4.70e-06	0.21
1_20793655_C_T_b37	rs61781160	5.40e-06	0.37
1_20792099_C_T_b37	rs618041	6.10e-06	0.34
1_20883422_T_C_b37	rs675397	7.90e-06	0.19
1_20789645_A_G_b37	rs12023000	1.20e-05	0.35

Table S12. GTEx visceral adipose tissue *cis*-eQTLs significantly associated with *CAMK2N1* expression and cardio-metabolic traits

SNP Id	*P*-value*	Effect Size	Cardiometabolic trait†	*P*-value‡	OR/ β-coefficient§
rs4654840	1.90e-15	-0.43	Coronary heart disease	0.0164	OR=0.976
			T2D adjBMI	0.017	OR=0.959
			HbA1c	0.042	β=-0.0669
rs35862517	2.20e-15	-0.43	Coronary heart disease	0.017	OR=0.976
			T2D adjBMI	0.022	OR=0.96
rs10916722	4.00e-15	-0.43	T2D adjBMI Coronary	0.028	OR=0.962
			heart disease	0.0321	OR=0.978
rs10916723	4.00e-15	-0.43	Coronary heart disease	0.0154	OR=0.975
			T2D adjBMI	0.019	OR=0.96
rs2213801	5.60e-15	-0.43	T2D adjBMI	0.019	OR=0.96
			VAT volume[11]	0.029	n/a
			Coronary artery disease	0.0319	OR=0.978
rs3921122	1.80e-14	-0.43	T2DM adjBMI	0.03	OR=0.963
			Coronary artery disease	0.0325	OR=0.978
rs10737446	6.80e-15	-0.42	Coronary artery disease	0.0193	OR=0.976
			Fasting glucose	0.0194	β=-0.0153
			T2DM adjBMI	0.034	OR=0.964
			HbA1c	0.039	β=-0.0671
rs6426621	6.90e-15	-0.42	T2DM adjBMI Coronary	0.018	OR=0.96
			artery disease	0.0215	OR=0.977
rs11807163	1.10e-14	-0.42	Coronary artery disease	0.0136	OR=0.975
			T2DM adjBMI	0.019	OR=0.96
			HbA1c	0.043	β=-0.0666
rs10737445	1.30e-14	-0.42	Coronary artery disease	0.0169	OR=0.976
			T2DM adjBMI	0.021	OR=0.96
rs2213800	1.80e-14	-0.42	Coronary artery disease	0.0195	OR=0.976
			T2DM adjBMI	0.022	OR=0.961
rs6685397	3.60e-14	-0.42	T2DM adjBMI	0.017	OR=0.0958
			HbA1c	0.035	β=-0.00704
			Systolic blood pressure	0.043	β=0.0633
			Fasting glucose	0.0466	β=-0.0073
rs10799616	3.80e-14	-0.41	T2DM adjBMI	0.022	OR=0.961
			Coronary artery disease	0.0235	OR=0.977
rs7536516	4.90e-14	-0.41	T2DM adjBMI	0.02	OR=0.96
			Coronary artery disease	0.0203	OR=0.977
			HbA1c	0.045	β=-0.066
rs2227227	6.10e-15	-0.41	Coronary heart disease	0.0173	OR=0.976
			T2DM adjBMI	0.032	OR=0.964
			Fasting glucose	0.0452	β=-0.012
rs739274	1.30e-14	-0.41	Coronary artery disease	0.0218	OR=0.977
			T2DM adjBMI	0.03	OR=0.963
			HbA1c	0.047	β=-0.0652
rs10916721	1.40e-14	-0.41	Coronary artery disease	0.0216	OR=0.977
			T2DM adjBMI	0.028	OR=0.962
			HbA1c	0.0331	β=-0.0722
			VAT volume	0.045	n/a
rs6687047	7.20e-14	-0.40	T2DM adjBMI	0.019	OR=0.96
			Coronary artery disease	0.0229	OR=0.977
			HbA1c	0.045	β=-0.066
rs7533911	1.80e-12	-0.40	T2DM adjBMI	0.022	OR=0.961
			Coronary artery disease	0.0222	OR=0.977

rs10916724	6.20e-13	-0.40	T2DM adjBMI	0.021	OR=0.96
rs10916725	5.20e-13	-0.39	T2DM	0.021	OR=0.96
rs56340248	7.80e-13	-0.39	T2DM adjBMI	0.036	OR=0.964
rs10799615	9.70e-13	-0.37	HbA1c	0.016	β=-0.0779
			Fasting insulin	0.0173	β=0.0451
			VAT volume	0.042	n/a
rs35015448	4.60e-09	-0.29	T2DM adjBMI	0.011	OR=0.959
rs12725354	4.90e-09	-0.29	T2DM adjBMI	0.012	OR=0.959
rs2097532	5.90e-09	-0.29	Fasting glucose	0.0257	β=-0.0076
			Coronary artery disease	0.0462	OR=0.981
rs34668419	5.20e-08	-0.26	Coronary artery disease	0.023	OR=0.976
			T2DM adjBMI	0.043	OR=0.967
rs10799613	5.70e-06	0.26	Modified Stumvoli ISI[12]	0.0388	β=-0.025
			VAT volume	0.042	n/a
rs6426617	2.40e-06	0.27	T2DM adjBMI	0.012	OR=1.02
			VAT volume	0.014	n/a
			Modified Stumvoli ISI	0.0254	β=-0.027
rs7535938	1.30e-06	0.28	T2DM adjBMI	0.012	OR=1.02
			VAT volume	0.014	n/a
			Modified Stumvoli ISI	0.0238	β=-0.027
rs6659296	5.50e-09	0.29	T2DM adjBMI	0.0082	OR=1.04
			Coronary artery disease	0.0388	OR=1.02
rs7542020	3.80e-07	0.29	VAT volume	0.014	n/a
			Fasting insulin	0.0156	β=0.0458
			Modified Stumvoli ISI	0.0252	β=-0.026
			T2DM adjBMI	0.029	OR=1.02
			Fasting glucose	0.0372	β=0.0723
rs6691280	4.30e-07	0.30	Coronary artery disease	0.0273	OR=1.02
			T2DM adjBMI	0.048	OR=1.03
rs719683	6.50e-11	0.32	T2DM adjBMI	0.0052	OR=1.05
			Fasting glucose	0.0147	β=0.0088
			HbA1c	0.015	β=0.0778
			Coronary artery disease	0.0196	OR=1.02
rs6668468	8.90e-08	0.32	Coronary artery disease	0.038	OR=1.02
rs10157172	1.90e-09	0.33	T2DM adjBMI	0.019	OR=1.02
rs12141257	3.40e-08	0.33	T2DM	0.037	OR=1.02
rs12141258	3.40e-08	0.33	T2DM adjBMI	0.02	OR=1.02
rs6426619	2.50e-08	0.33	Coronary artery disease	0.0168	OR=1.03
rs10753484	3.50e-08	0.33	T2DM adjBMI	0.035	OR=1.04
			Coronary artery disease	0.0413	OR=1.02
rs3820320	8.80e-09	0.34	Coronary artery disease	0.0136	OR=1.03
			T2DM adjBMI	0.027	OR=1.04
			Fasting glucose	0.0382	β=0.0738
rs1061588	4.80e-09	0.35	T2D adjBMI	0.036	OR=1.04
rs10916720	1.10e-08	0.35	T2DM adjBMI	0.021	OR=1.04
			Coronary artery disease	0.0474	OR=1.02
rs10799614	1.90e-05	0.40	Two-hour glucose	0.0363	β=0.0762
			T2DM adjBMI	0.04	OR=1.03

*Significance of the association between the variant and the effect size of *CAMK2N1* expression.

†Cardiometabolic trait associations found in http://type2diabetesgenetics.org and http://www.broadcvdi.org. accessed 2019-04-01.

[‡]Significance of the association between the variant and the cardiometabolic trait.
[§]β-coefficient represents the magnitude and direction of association between a variant and a trait and is analogous to Odds Ratio.
Abbreviations: HbA1c, glycated haemoglobin; ISI, insulin sensitivity index; n/a information not available; OR, odds ratio; T2DM adjBMI, type 2 diabetes adjusted for body mass index; VAT, visceral adipose tissue.

Table S13. A set of GTex assigned *cis*-eQTLs in Adipose Visceral (Omentum) blindly selected from one of a 1000 sets of 263 random *cis*-eQTLs that are not associated with *CAMK2N1* expression and their association with cardio-metabolic traits in the T2D Knowledge Portal

SNP Id	Cardiometabolic disease trait*	*P*-value†	OR/ β-coefficient‡
rs4371471	T2DM	0.019	0.98
	Coronary artery disease	0.00278	1.03
rs1112463	T2DM	0.049	0.934
rs55777955	T2DM	0.03	0.952
	Coronary artery disease	0.00266	1.02
rs78245474	n/a§	n/a	n/a
rs55812375	n/t‖	n/t	NT
rs4776659	HbA1c	0.0354	n/a
rs59377023	T2DM	0.0197	1.07
rs10790169	T2DM	0.0037	0.948
rs1040806	T2DM	0.004	1.04
rs13091746	n/t	n/t	NT
rs397871779	n/a	n/a	n/a
rs35333201	n/t	n/t	NT
rs72833634	T2DM	0.00062	1.06
rs59278083	n/a	n/a	n/a
rs4291309	T2DM	0.023	1.02
rs9468204	n/t	n/t	NT
rs9596144	T2DM	0.0034	1.03
rs7204617	HbA1c	0.017	n/a
rs9371173	T2DM	0.0482	0.893
rs796383018	n/a	n/a	n/a
rs10127452	n/a	n/a	n/a
rs72760337	T2DM	0.024	0.978
rs2438272	n/t	n/t	NT
rs2050189	Fasting glucose	0.00963	n/a
rs4912439	Systolic blood pressure	0.031	n/a
rs8060108	Body fat %	0.0357	n/a
rs111579015	HbA1c	0.05	n/a
rs4963679	n/t	n/t	NT
rs1613078	T2DM	0.0259	0.932
	Coronary artery disease	0.0443	0.982
rs12515366	T2DM	0.0098	1.2
rs4608623	T2DM	0.00002	1.03
	VAT	0.0099	
rs62440561	n/t	n/t	NT
rs13292961	n/t	n/t	NT
rs9380500	n/t	n/t	NT
rs28365957	n/t	n/t	NT
rs59395091	n/a	n/a	n/a

rs13418943	T2DM	0.0295	0.93
rs12767	HbA1c	0.034	n/a
rs2892240	HbA1c	0.024	n/a
rs11053179	T2DM	0.0049	0.982
rs34993870	HbA1c	0.00041	n/a
rs12773034	n/t	n/t	NT
rs140506769	HbA1c	0.047	n/a
rs13280927	T2DM	0.032	1.02
rs76663184	n/t	n/t	NT
rs7088929	T2DM	0.0361	0.88
rs1942893	Systolic blood pressure	0.035	n/a
rs11656629	n/a	n/a	n/a
rs17826173	T2DM	0.001	0.963
rs8112380	T2DM	0.0087	0.922
rs10175281	HbA1c	0.0414	n/a
rs62133396	Systolic blood pressure	0.024	n/a
rs11023369	T2DM	0.0225	0.98
rs7133447	T2DM	0.045	0.985
rs761587	T2DM	0.011	0.831
rs62580717	n/t	n/t	NT
rs9904352	n/t	n/t	NT
rs73322250	HbA1c	0.039	n/a
rs6975345	T2DM	0.208	1.06

*Cardiometabolic trait associations found in http://type2diabetesgenetics.org and http://www.broadcvdi.org. accessed 2019-04-01.

†Significance of the association between the variant and the cardiometabolic trait.

‡β-coefficient represents the magnitude and direction of association between a variant and a trait.

§No data available from T2D Knowledge Portal.

||No traits relevant to the *Camk2n1*$^{-/-}$ rat cardiometabolic phenotypes.

Abbreviations: HbA1c, glycated haemoglobin; ISI, insulin sensitivity index; n/a information not available; OR, odds ratio;T2DM adjBMI, type 2 diabetes adjusted for body mass index; VAT, visceral adipose tissue.

Table S14. Contingency tables comparing the three main cardiometabolic disease trait associations observed with *CAMK2N1 cis*-eQTLs compared to non-*CAMK2N1 cis*-eQTLs in Adipose Visceral (Omentum)

**Observed**	T2DM SNP	non-T2DM SNP	TOTAL
CAMK2N1	44	8	52
non-*CAMK2N1*	24	35	59
TOTAL	68	43	111
**Expected**			
CAMK2N1	32	20	52
non-*CAMK2N1*	36	23	59
TOTAL	68	43	111
P (Chi-squared test)			2.12e-06
**Observed**	CAD SNP	non-CAD SNP	TOTAL
CAMK2N1	27	25	52
non-*CAMK2N1*	3	56	59
TOTAL	30	81	111
**Expected**			
CAMK2N1	14	38	52
non-*CAMK2N1*	16	43	59
TOTAL	30	81	111
P (Chi-squared test)			2.94e-08
**Observed**	VAT SNP	non-VAT SNP	TOTAL
CAMK2N1	7	45	52
non-*CAMK2N1*	1	58	59
TOTAL	8	103	111
**Expected**			
CAMK2N1	4	48	52
non-*CAMK2N1*	4	55	59
TOTAL	8	103	111
P (Chi-squared test)			1.68e-02

Abbreviations: *CAMK2N1*, GTex assigned cis-eQTLs in Adipose – Visceral (Omentum) associated with *CAMK2N1* expression; non-CAMK2N1, GTex assigned *cis*-eQTL in Adipose – Visceral (Omentum) selected from a random set of 263 *cis*-eQTLs in multiple tissues that are not associated with *CAMK2N1* expression; CAD,coronary artery disease; CAD SNP, GTex assigned *cis*-eQTL in Adipose – Visceral (Omentum) associated with CAD in the T2D Knowledge Portal; non-CAD SNP, *cis*-eQTL in Adipose – Visceral (Omentum) not associated with CAD; T2DM, type 2 diabetes mellitus; T2DM SNP, GTex assigned *cis*-eQTL in Adipose – Visceral (Omentum) associated with T2DM in the T2D Knowledge Portal; non-T2DM SNP, *cis*-eQTL in Adipose – Visceral (Omentum) not associated with T2DM; VAT, visceral adipose tissue volume; VAT SNP, GTex assigned *cis*-eQTL in Adipose – Visceral (Omentum) associated with VAT volume in the T2D Knowledge Portal; non-VAT SNP, *cis*-eQTL in Adipose – Visceral (Omentum) not associated with VAT volume.

Table S15. Patient anthropomorphic data

Parameter	Lean	Obese	Obese/T2D
Age (years)	53.7 ±5.31	59.1 ±2.33	61.4 ±2.89
Sex	4M, 6F	5M, 4F	6M, 3F
Height (m)	1.74 ±0.04	1.67 ±0.04	1.67 ±0.05
Weight (kg)	68.9 ±1.17	107.1 ±4.50‡	121.1 ±12.6*
BMI	22.9 ±0.52	37.9 ±1.83‡	45.0 ±3.08‡
Fat mass (kg)	19.60 ±1.48	42.7 ±3.83†	56.14 ±6.10‡
% Fat mass	29.32 ±2.33	38.59 ±2.09	42.1±1.51†
SBP (mmHg)	122.1 ±4.4	134.0 ±3.98	136.1 ±4.57
DBP (mmHg)	73.6 ±2.15	79.9 ±3.36	74.2 ±2.48*

Mean ±SEM. Statistical significances between lean and obese or obese/T2D: **P*<0.01, †*P*<0.001, ‡*P*<0.0001.

Abbreviations: BMI, body mass index; DBP, diastolic blood pressure; SBP, systolic blood pressure.

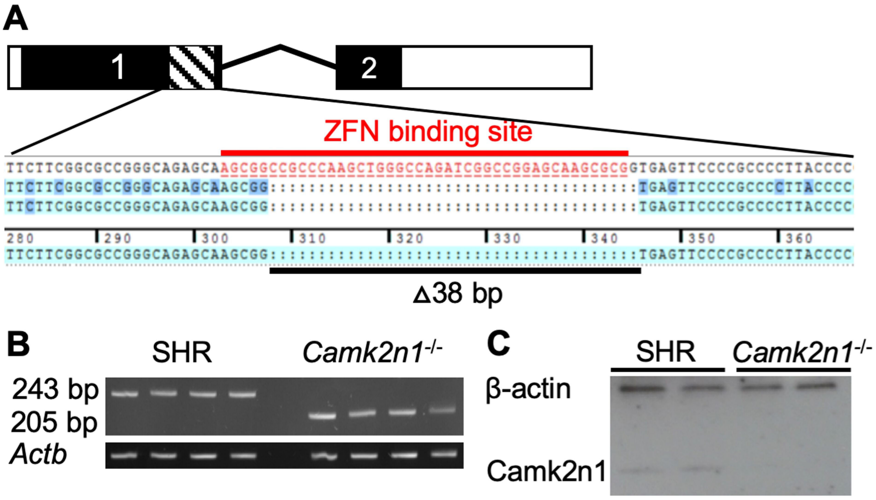

Figure S1. *Camk2n1* knockout generation and validation. (A) Schematic diagram of rat *Camk2n1* with genomic DNA sequence showing 38bp deletion in exon 1 of *Camk2n1*. (B) Agarose gel image of cDNA PCR products of *Camk2n1* using primers spanning ZFN deletion. (C) Autoradiograph detection of *Camk2n1* and β-actin protein extracted from whole brain.

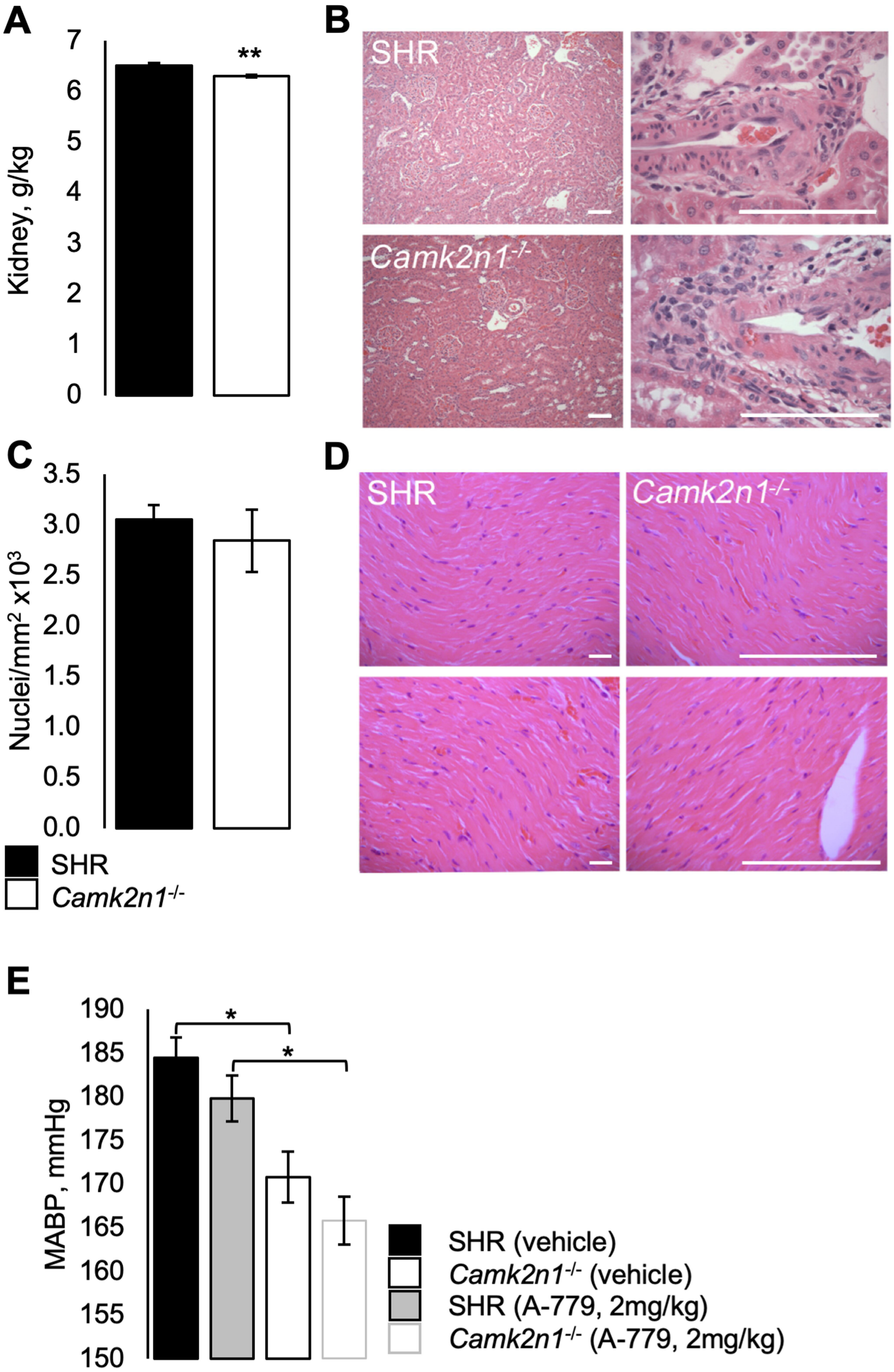

Figure S2. Histological analysis of renal and cardiac structure morphology and *in vivo* vasoreactivity with Mas receptor blockage in SHR and *Camk2n1*$^{-/-}$ rats. (A) Relative kidney wet mass. (B) Representative light micrographs of H&E stained kidney at x10 and x60 magnification (scale bar 100 μM). (C) Cardiomyocyte nuclear density. (D) Representative light micrographs of H&E stained left ventricle at x40 (scale bar 100 μM). (E) Mean arterial blood pressure (MABP) *in vivo* in the presence of Mas receptor antagonist A-779 (2mg/kg). Mean ± SEM n=5-7/group, tissue weights; n=3/group histological analysis. Significant differences between genotype **P*<0.01.

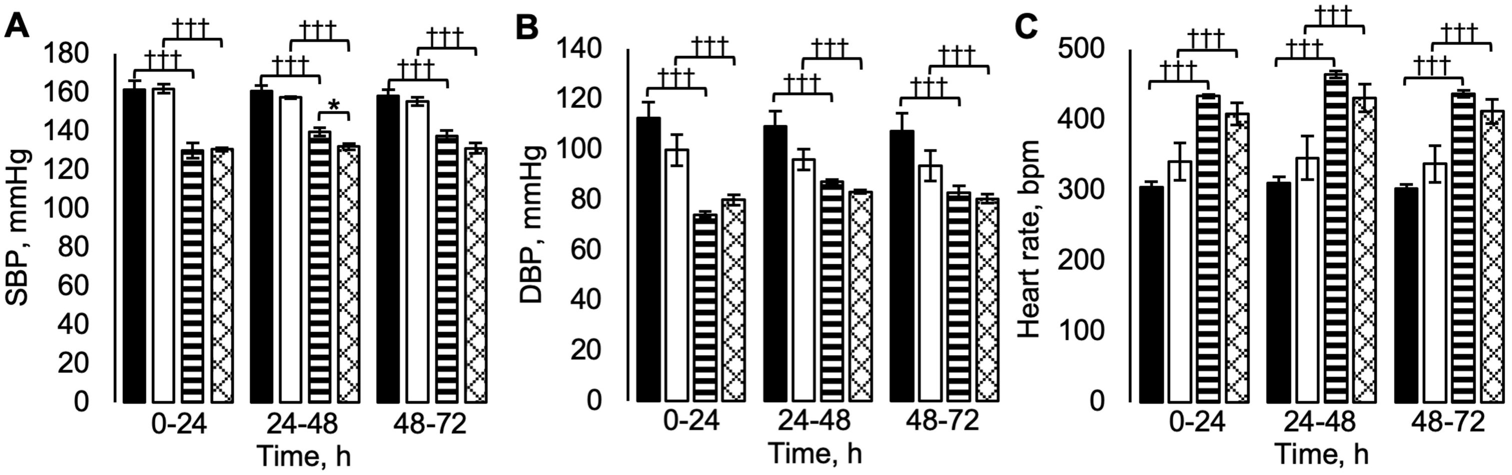

Figure S3. Isoproterenol-stimulated cardiovascular physiological and molecular phenotypes in SHR and *Camk2n1*$^{-/-}$ rats (A) Systolic (SBP) and (B) diastolic (DBP) blood pressures and (C) heart rate. Mean ± SEM n=5-7/group; significant differences between treatments (†*P*<0.05, ††*P*<0.01, †††*P*<0.001

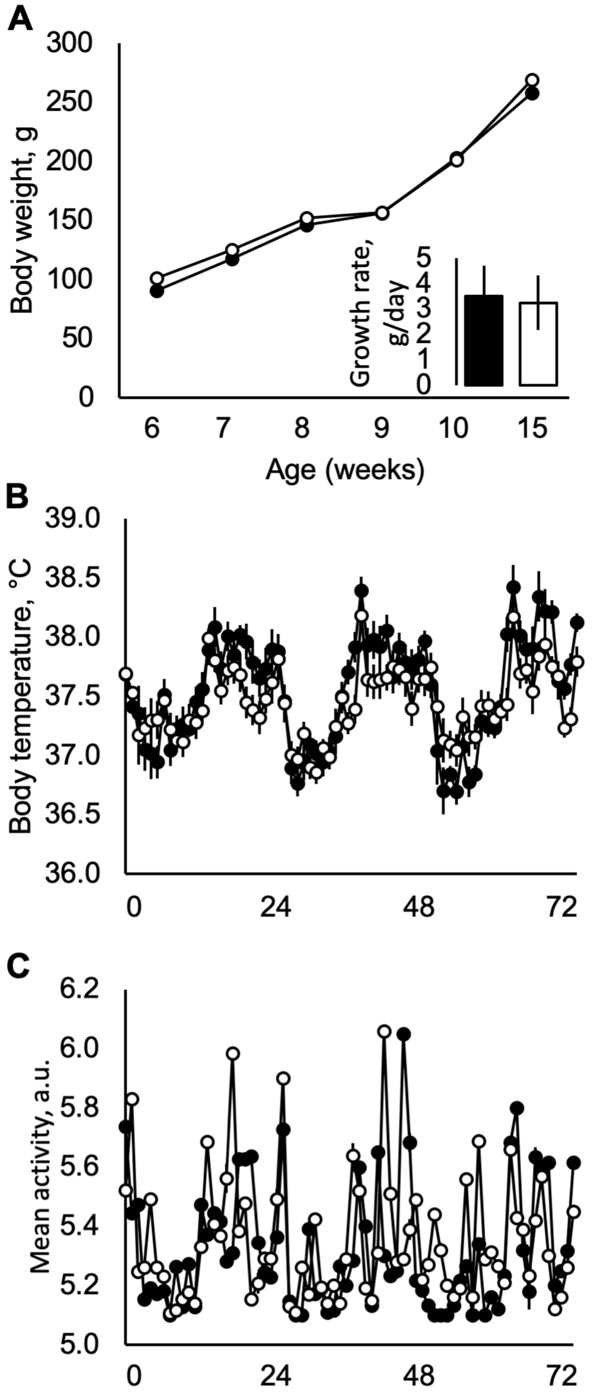

Figure S4. Body weight, core temperature and locomotor activity in SHR and *Camk2n1*$^{-/-}$ rats. (A) Body weight was measured from 6-15 weeks age. (B) Core body temperature and (C) locomotor activity were collected using radiotelemetry transmitters during blood pressure measurement.

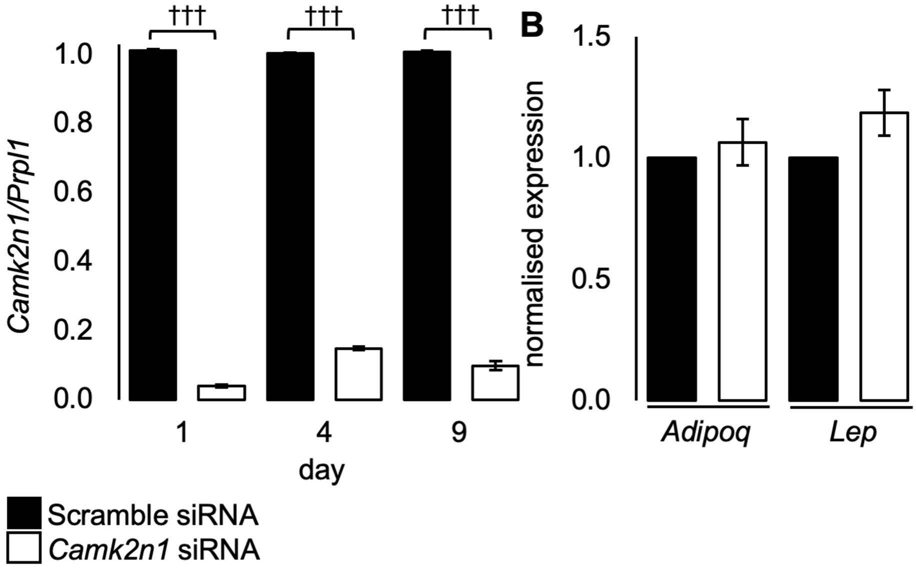

Figure S5. Expression of Camk2n1 throughout in vitro adipogenesis and adipokine expression at day 9 in 3T3-L1 differentiated fibroblasts. Cells incubated with vehicle, scramble, *Camk2n1* siRNA. (A) *Camk2n1* expression 1, 4, and 9 days after knockdown (KD). (B) Assessment of adipocyte differentiation 9 days following knockdown (KD) by quantifying *Adipoq* and *Lep* transcript levels at 9 days after KD. Mean ±SEM n=3 experiments. Significant differences between treatments (†††P<0.001).

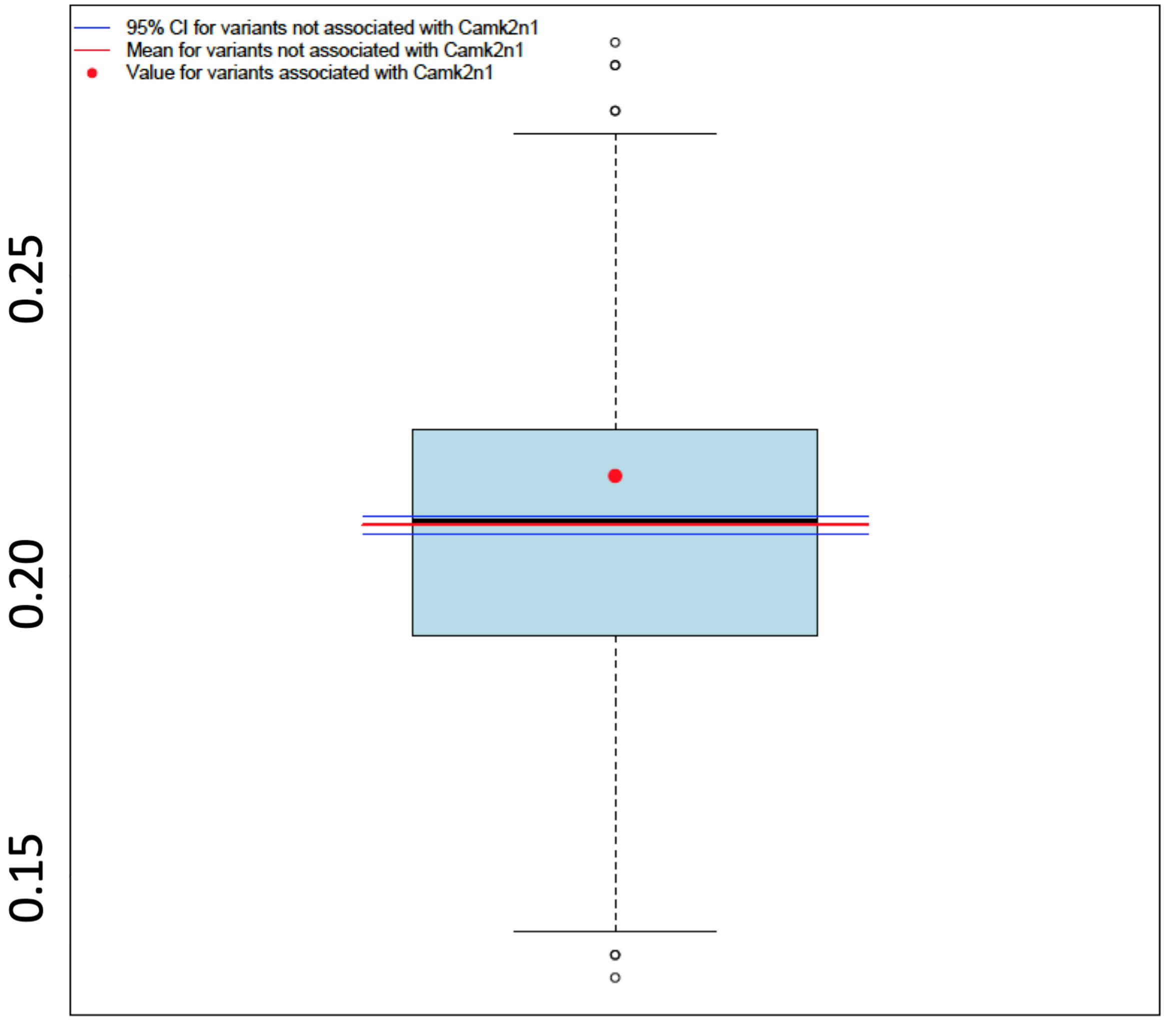

Figure S6. Box plot of proportion of genomic variants associated with or not associated with *CAMK2N1* and Adipose – Visceral (Omentum). Mean and 95% confidence intervals of the proportion of *cis*-eQTLs in 1000 sets of randomly selected *cis*-eQTLs not associated with *CAMK2N1* expression compared to the proportion of *CAMK2N1 cis*-eQTLs in Adipose – Visceral (Omentum) from the GTex Knowledge Portal.
