# Supplementary figures and images for: Camk2n1 Is a Negative Regulator of Blood Pressure, Left Ventricular Mass, Insulin Sensitivity, and Promotes Adiposity
Source: Hypertension. 2019 Jul 22;74(3):687–96. doi: 10.1161/HYPERTENSIONAHA.118.12409 (PMC6686962; doi:10.1161/HYPERTENSIONAHA.118.12409)

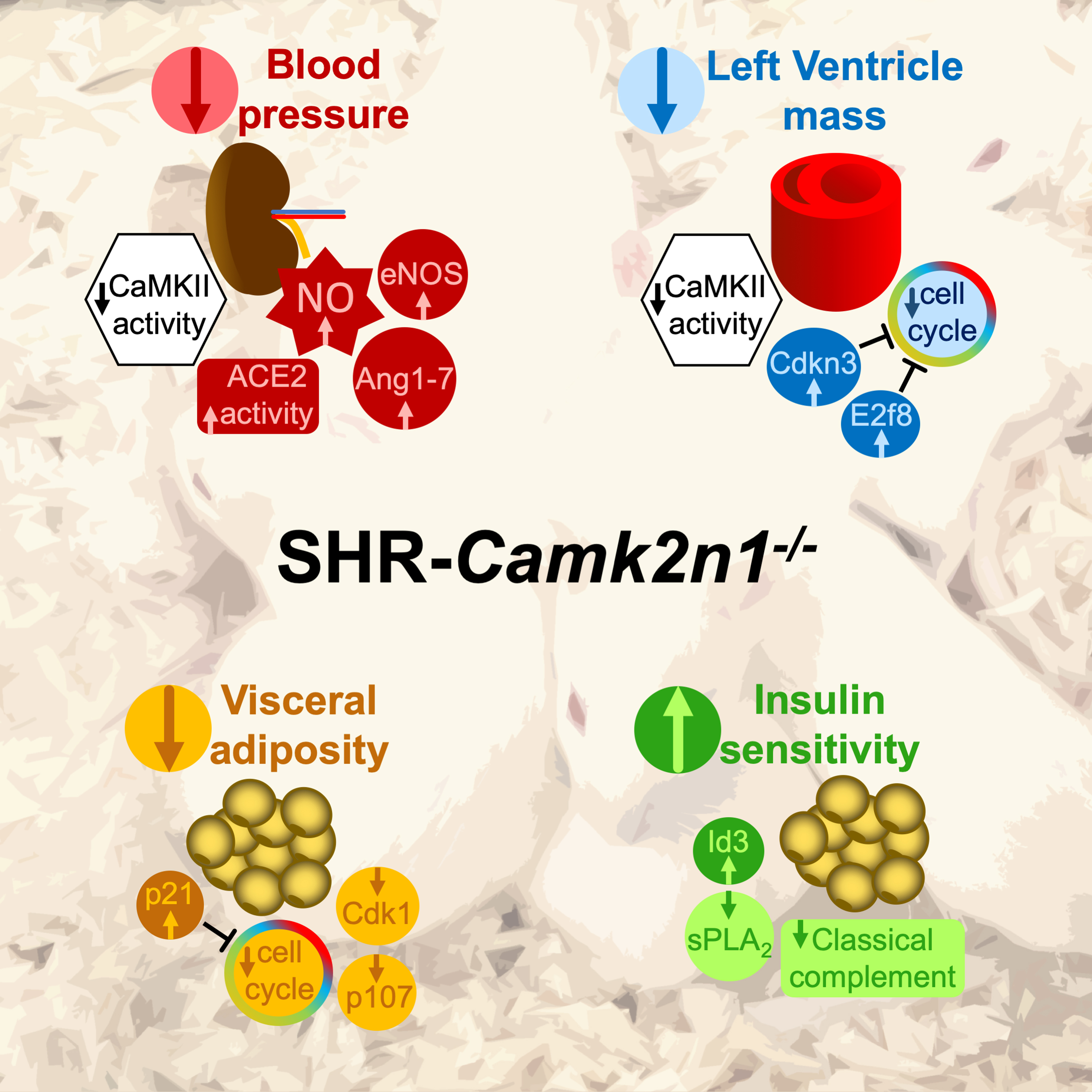

Supplement: Supplementary file 2 [file hyp-74-687-s002.jpg]
