# Supplementary material for: Camk2n1 Is a Negative Regulator of Blood Pressure, Left Ventricular Mass, Insulin Sensitivity, and Promotes Adiposity
Source: Hypertension. 2019 Jul 22;74(3):687–96. doi: 10.1161/HYPERTENSIONAHA.118.12409 (PMC6686962; doi:10.1161/HYPERTENSIONAHA.118.12409)
Supplement: Supplementary file 3 [file hyp-74-687-s003.pdf]

Change of Authorship Form

(Must be completed and signed by ALL authors)

Please check all that apply

✓ New author(s) have been added (in addition to this form, all new authors must complete the copyright transfer agreement and conflict of interest disclosure.

_____Change in order of authorship.

_____An author wishes to remove his/her name. An author's name may only be removed his/her own request and a letter signed by the author should accompany this form

Manuscript Number HYPE201812409

Manuscript Title Camk2n1 is a negative regulator of blood pressure, left ventricular mass, insulin sensitivity and promotes adiposity

Former Authorship

Please list ALL AUTHORS in the same order as the original submission. For more than 12, use an extra sheet.

Print Name	Print Name
Name (1) Neza Alfazema	Name (7) Ben Moyon
Name (2) Marjorie Barrier	Name (8) Zoe Webster
Name (3) Sophie Marion de Proce	Name (9) Christopher Bellamy
Name (4) Robert Menzies	Name (10) Mark Arends
Name (5) Roderick Carter	Name (11) Roland Stimson
Name (6) Ana Garcia Diaz	Name (12) Nicholas Morton

New Authorship

All authors must sign below agreeing to the changes in authorship. The authorship order must reflect the authorship order of the manuscript.

Name	Signature	Date
Name (1) Neza Alfazema	Signature N. Alfazema	Date 5/3/19
Name (2) Marjorie Barrier	Signature	Date
Name (3) Sophie Marion de Proce	Signature	Date
Name (4) Robert Menzies	Signature	Date
Name (5) Roderick Carter	Signature	Date
Name (6) Kevin Stewart	Signature	Date
Name (7) Ana Garcia Diaz	Signature	Date
Name (8) Ben Moyon	Signature	Date
Name (9) Zoe Webster	Signature	Date
Name (10) Christopher Bellamy	Signature	Date
Name (11) Mark Arends	Signature	Date
Name (12) Roland Stimson	Signature	Date

Please print form and fax to 214-706-1565.

Change of Authorship Form

(Must be completed and signed by ALL authors)

Please check all that apply

__✓__ New author(s) have been added (in addition to this form, all new authors must complete the copyright transfer agreement and conflict of interest disclosure.

_____ Change in order of authorship.

_____ An author wishes to remove his/her name. An author's name may only be removed his/her own request and a letter signed by the author should accompany this form

Manuscript Number HYPE201812409

Manuscript Title Camk2n1 is a negative regulator of blood pressure, left ventricular mass, insulin sensitivity and promotes adiposity

Former Authorship

Please list ALL AUTHORS in the same order as the original submission. For more than 12, use an extra sheet.

**Print Name**		**Print Name**	
Name (1)	Neza Alfazema	Name (7)	Ben Moyon
Name (2)	Marjorie Barrier	Name (8)	Zoe Webster
Name (3)	Sophie Marion de Proce	Name (9)	Christopher Bellamy
Name (4)	Robert Menzies	Name (10)	Mark Arends
Name (5)	Roderick Carter	Name (11)	Roland Stimson
Name (6)	Ana Garcia Diaz	Name (12)	Nicholas Morton

New Authorship

All authors must sign below agreeing to the changes in authorship. The authorship order must reflect the authorship order of the manuscript.

Name		Signature	Date
Name (1)	Neza Alfazema		
Name (2)	Marjorie Barrier		25-02-2019
Name (3)	Sophie Marion de Proce		
Name (4)	Robert Menzies		
Name (5)	Roderick Carter		
Name (6)	Kevin Stewart		
Name (7)	Ana Garcia Diaz		
Name (8)	Ben Moyon		
Name (9)	Zoe Webster		
Name (10)	Christopher Bellamy		
Name (11)	Mark Arends		
Name (12)	Roland Stimson		

Please print form and fax to 214-706-1565.

Change of Authorship Form

(Must be completed and signed by ALL authors)

Please check all that apply

✔ New author(s) have been added (in addition to this form, all new authors must complete the copyright transfer agreement and conflict of interest disclosure.

_____Change in order of authorship.

_____An author wishes to remove his/her name. An author's name may only be removed his/her own request and a letter signed by the author should accompany this form

Manuscript Number HYPE201812409

Manuscript Title Camk2n1 is a negative regulator of blood pressure, left ventricular mass, insulin sensitivity and promotes adiposity

Former Authorship

Please list ALL AUTHORS in the same order as the original submission. For more than 12, use an extra sheet.

Print Name		Print Name	
Name (1)	Neza Alfazema	Name (7)	Ben Moyon
Name (2)	Marjorie Barrier	Name (8)	Zoe Webster
Name (3)	Sophie Marion de Proce	Name (9)	Christopher Bellamy
Name (4)	Robert Menzies	Name (10)	Mark Arends
Name (5)	Roderick Carter	Name (11)	Roland Stimson
Name (6)	Ana Garcia Diaz	Name (12)	Nicholas Morton

New Authorship

All authors must sign below agreeing to the changes in authorship. The authorship order must reflect the authorship order of the manuscript.

Name		Signature	Date
Name (1)	Neza Alfazema		
Name (2)	Marjorie Barrier		
Name (3)	Sophie Marion de Proce	Mariondeproce	28th February 2019
Name (4)	Robert Menzies		
Name (5)	Roderick Carter		
Name (6)	Kevin Stewart		
Name (7)	Ana Garcia Diaz		
Name (8)	Ben Moyon		
Name (9)	Zoe Webster		
Name (10)	Christopher Bellamy		
Name (11)	Mark Arends		
Name (12)	Roland Stimson		

Please print form and fax to 214-706-1565.

Change of Authorship Form

(Must be completed and signed by ALL authors)

Please check all that apply

__✓__ New author(s) have been added (in addition to this form, all new authors must complete the copyright transfer agreement and conflict of interest disclosure.

_____ Change in order of authorship.

_____ An author wishes to remove his/her name. An author's name may only be removed his/her own request and a letter signed by the author should accompany this form

Manuscript Number HYPE201812409

Manuscript Title Camk2n1 is a negative regulator of blood pressure, left ventricular mass, insulin sensitivity and promotes adiposity

Former Authorship

Please list ALL AUTHORS in the same order as the original submission. For more than 12, use an extra sheet.

Print Name		Print Name	
Name (1)	Neza Alfazema	Name (7)	Ben Moyon
Name (2)	Marjorie Barrier	Name (8)	Zoe Webster
Name (3)	Sophie Marion de Proce	Name (9)	Christopher Bellamy
Name (4)	Robert Menzies	Name (10)	Mark Arends
Name (5)	Roderick Carter	Name (11)	Roland Stimson
Name (6)	Ana Garcia Diaz	Name (12)	Nicholas Morton

New Authorship

All authors must sign below agreeing to the changes in authorship. The authorship order must reflect the authorship order of the manuscript.

	Name	Signature	Date
Name (1)	Neza Alfazema	N. Alfazema	25-02-19
Name (2)	Marjorie Barrier		
Name (3)	Sophie Marion de Proce		
Name (4)	Robert Menzies	[illegible]	27-02-19
Name (5)	Roderick Carter		
Name (6)	Kevin Stewart		
Name (7)	Ana Garcia Diaz		
Name (8)	Ben Moyon		
Name (9)	Zoe Webster		
Name (10)	Christopher Bellamy		
Name (11)	Mark Arends		
Name (12)	Roland Stimson		

Please print form and fax to 214-706-1565.

Change of Authorship Form

(Must be completed and signed by ALL authors)

Please check all that apply

__✔__ New author(s) have been added (in addition to this form, all new authors must complete the copyright transfer agreement and conflict of interest disclosure.

_____ Change in order of authorship.

_____ An author wishes to remove his/her name. An author's name may only be removed his/her own request and a letter signed by the author should accompany this form

Manuscript Number HYPE201812409

Manuscript Title Camk2n1 is a negative regulator of blood pressure, left ventricular mass, insulin sensitivity and promotes adiposity

Former Authorship

Please list ALL AUTHORS in the same order as the original submission. For more than 12, use an extra sheet.

Print Name		Print Name	
Name (1)	Neza Alfazema	Name (7)	Ben Moyon
Name (2)	Marjorie Barrier	Name (8)	Zoe Webster
Name (3)	Sophie Marion de Proce	Name (9)	Christopher Bellamy
Name (4)	Robert Menzies	Name (10)	Mark Arends
Name (5)	Roderick Carter	Name (11)	Roland Stimson
Name (6)	Ana Garcia Diaz	Name (12)	Nicholas Morton

New Authorship

All authors must sign below agreeing to the changes in authorship. The authorship order must reflect the authorship order of the manuscript.

	Name	Signature	Date
Name (1)	Neza Alfazema		
Name (2)	Marjorie Barrier		
Name (3)	Sophie Marion de Proce		
Name (4)	Robert Menzies		
Name (5)	Roderick Carter	Roderick Carter	25/02/19
Name (6)	Kevin Stewart		
Name (7)	Ana Garcia Diaz		
Name (8)	Ben Moyon		
Name (9)	Zoe Webster		
Name (10)	Christopher Bellamy		
Name (11)	Mark Arends		
Name (12)	Roland Stimson		

Please print form and fax to 214-706-1565.

Change of Authorship Form

(Must be completed and signed by ALL authors)

Please check all that apply

✓ New author(s) have been added (in addition to this form, all new authors must complete the copyright transfer agreement and conflict of interest disclosure.

_____Change in order of authorship.

_____An author wishes to remove his/her name. An author's name may only be removed his/her own request and a letter signed by the author should accompany this form

Manuscript Number HYPE201812409

Manuscript Title Camk2n1 is a negative regulator of blood pressure, left ventricular mass, insulin sensitivity and promotes adiposity

Former Authorship

Please list ALL AUTHORS in the same order as the original submission. For more than 12, use an extra sheet.

Print Name		Print Name	
Name (1)	Neza Alfazema	Name (7)	Ben Moyon
Name (2)	Marjorie Barrier	Name (8)	Zoe Webster
Name (3)	Sophie Marion de Proce	Name (9)	Christopher Bellamy
Name (4)	Robert Menzies	Name (10)	Mark Arends
Name (5)	Roderick Carter	Name (11)	Roland Stimson
Name (6)	Ana Garcia Diaz	Name (12)	Nicholas Morton

New Authorship

All authors must sign below agreeing to the changes in authorship. The authorship order must reflect the authorship order of the manuscript.

Name		Signature	Date
Name (1)	Neza Alfazema		
Name (2)	Marjorie Barrier		
Name (3)	Sophie Marion de Proce		
Name (4)	Robert Menzies		
Name (5)	Roderick Carter		
Name (6)	Kevin Stewart	[illegible]	25.2.19
Name (7)	Ana Garcia Diaz		
Name (8)	Ben Moyon		
Name (9)	Zoe Webster		
Name (10)	Christopher Bellamy		
Name (11)	Mark Arends		
Name (12)	Roland Stimson		

Please print form and fax to 214-706-1565.

Change of Authorship Form

(Must be completed and signed by ALL authors)

Please check all that apply

__✓__ New author(s) have been added (in addition to this form, all new authors must complete the copyright transfer agreement and conflict of interest disclosure.

_____ Change in order of authorship.

_____ An author wishes to remove his/her name. An author's name may only be removed his/her own request and a letter signed by the author should accompany this form

Manuscript Number HYPE201812409

Manuscript Title Camk2n1 is a negative regulator of blood pressure, left ventricular mass, insulin sensitivity and promotes adiposity

Former Authorship

Please list ALL AUTHORS in the same order as the original submission. For more than 12, use an extra sheet.

Print Name	Print Name
Name (1) Neza Alfazema	Name (7) Ben Moyon
Name (2) Marjorie Barrier	Name (8) Zoe Webster
Name (3) Sophie Marion de Proce	Name (9) Christopher Bellamy
Name (4) Robert Menzies	Name (10) Mark Arends
Name (5) Roderick Carter	Name (11) Roland Stimson
Name (6) Ana Garcia Diaz	Name (12) Nicholas Morton

New Authorship

All authors must sign below agreeing to the changes in authorship. The authorship order must reflect the authorship order of the manuscript.

Name	Signature	Date
Name (1) Neza Alfazema	Signature	Date
Name (2) Marjorie Barrier	Signature	Date
Name (3) Sophie Marion de Proce	Signature	Date
Name (4) Robert Menzies	Signature	Date
Name (5) Roderick Carter	Signature	Date
Name (6) Kevin Stewart	Signature	Date
Name (7) Ana Garcia Diaz	Signature [signed]	Date
Name (8) Ben Moyon	Signature	Date
Name (9) Zoe Webster	Signature	Date
Name (10) Christopher Bellamy	Signature	Date
Name (11) Mark Arends	Signature	Date
Name (12) Roland Stimson	Signature	Date

Please print form and fax to 214-706-1565.

Change of Authorship Form

(Must be completed and signed by ALL authors)

Please check all that apply

✓ New author(s) have been added (in addition to this form, all new authors must complete the copyright transfer agreement and conflict of interest disclosure.

_____Change in order of authorship.

_____An author wishes to remove his/her name. An author's name may only be removed his/her own request and a letter signed by the author should accompany this form

Manuscript Number HYPE201812409

Manuscript Title Camk2n1 is a negative regulator of blood pressure, left ventricular mass, insulin sensitivity and promotes adiposity

Former Authorship

Please list ALL AUTHORS in the same order as the original submission. For more than 12, use an extra sheet.

Print Name		Print Name	
Name (1)	Neza Alfazema	Name (7)	Ben Moyon
Name (2)	Marjorie Barrier	Name (8)	Zoe Webster
Name (3)	Sophie Marion de Proce	Name (9)	Christopher Bellamy
Name (4)	Robert Menzies	Name (10)	Mark Arends
Name (5)	Roderick Carter	Name (11)	Roland Stimson
Name (6)	Ana Garcia Diaz	Name (12)	Nicholas Morton

New Authorship

All authors must sign below agreeing to the changes in authorship. The authorship order must reflect the authorship order of the manuscript.

Name		Signature	Date
Name (1)	Neza Alfazema		
Name (2)	Marjorie Barrier		
Name (3)	Sophie Marion de Proce		
Name (4)	Robert Menzies		
Name (5)	Roderick Carter		
Name (6)	Kevin Stewart		
Name (7)	Ana Garcia Diaz		
Name (8)	Ben Moyon		25/02/19
Name (9)	Zoe Webster		
Name (10)	Christopher Bellamy		
Name (11)	Mark Arends		
Name (12)	Roland Stimson		

Please print form and fax to 214-706-1565.

Change of Authorship Form

(Must be completed and signed by ALL authors)

Please check all that apply

✔ New author(s) have been added (in addition to this form, all new authors must complete the copyright transfer agreement and conflict of interest disclosure.

_____Change in order of authorship.

_____An author wishes to remove his/her name. An author's name may only be removed his/her own request and a letter signed by the author should accompany this form

Manuscript Number HYPE201812409

Manuscript Title Camk2n1 is a negative regulator of blood pressure, left ventricular mass, insulin sensitivity and promotes adiposity

Former Authorship

Please list ALL AUTHORS in the same order as the original submission. For more than 12, use an extra sheet.

**Print Name**	**Print Name**
Name (1) Neza Alfazema	Name (7) Ben Moyon
Name (2) Marjorie Barrier	Name (8) Zoe Webster
Name (3) Sophie Marion de Proce	Name (9) Christopher Bellamy
Name (4) Robert Menzies	Name (10) Mark Arends
Name (5) Roderick Carter	Name (11) Roland Stimson
Name (6) Ana Garcia Diaz	Name (12) Nicholas Morton

New Authorship

All authors must sign below agreeing to the changes in authorship. The authorship order must reflect the authorship order of the manuscript.

Name	Signature	Date
Name (1) Neza Alfazema		
Name (2) Marjorie Barrier		
Name (3) Sophie Marion de Proce		
Name (4) Robert Menzies		
Name (5) Roderick Carter		
Name (6) Kevin Stewart		
Name (7) Ana Garcia Diaz		
Name (8) Ben Moyon		
Name (9) Zoe Webster	[illegible]	26/2/19
Name (10) Christopher Bellamy		
Name (11) Mark Arends		
Name (12) Roland Stimson		

Please print form and fax to 214-706-1565.

Change of Authorship Form

(Must be completed and signed by ALL authors)

Please check all that apply

✓ New author(s) have been added (in addition to this form, all new authors must complete the copyright transfer agreement and conflict of interest disclosure.

_____Change in order of authorship.

_____An author wishes to remove his/her name. An author's name may only be removed his/her own request and a letter signed by the author should accompany this form

Manuscript Number HYPE201812409

Manuscript Title Camk2n1 is a negative regulator of blood pressure, left ventricular mass, insulin sensitivity and promotes adiposity

Former Authorship

Please list ALL AUTHORS in the same order as the original submission. For more than 12, use an extra sheet.

Print Name	Print Name
Name (1) Neza Alfazema	Name (7) Ben Moyon
Name (2) Marjorie Barrier	Name (8) Zoe Webster
Name (3) Sophie Marion de Proce	Name (9) Christopher Bellamy
Name (4) Robert Menzies	Name (10) Mark Arends
Name (5) Roderick Carter	Name (11) Roland Stimson
Name (6) Ana Garcia Diaz	Name (12) Nicholas Morton

New Authorship

All authors must sign below agreeing to the changes in authorship. The authorship order must reflect the authorship order of the manuscript.

Name	Signature	Date
Name (1) Neza Alfazema		
Name (2) Marjorie Barrier		
Name (3) Sophie Marion de Proce		
Name (4) Robert Menzies		
Name (5) Roderick Carter		
Name (6) Kevin Stewart		
Name (7) Ana Garcia Diaz		
Name (8) Ben Moyon		
Name (9) Zoe Webster		
Name (10) Christopher Bellamy	Chris Bellamy	5/3/19
Name (11) Mark Arends		
Name (12) Roland Stimson		

Please print form and fax to 214-706-1565.

Change of Authorship Form

(Must be completed and signed by ALL authors)

Please check all that apply

✓ New author(s) have been added (in addition to this form, all new authors must complete the copyright transfer agreement and conflict of interest disclosure.

_____ Change in order of authorship.

_____ An author wishes to remove his/her name. An author's name may only be removed his/her own request and a letter signed by the author should accompany this form

Manuscript Number HYPE201812409

Manuscript Title Camk2n1 is a negative regulator of blood pressure, left ventricular mass, insulin sensitivity and promotes adiposity

Former Authorship

Please list ALL AUTHORS in the same order as the original submission. For more than 12, use an extra sheet.

Print Name		Print Name	
Name (1)	Neza Alfazema	Name (7)	Ben Moyon
Name (2)	Marjorie Barrier	Name (8)	Zoe Webster
Name (3)	Sophie Marion de Proce	Name (9)	Christopher Bellamy
Name (4)	Robert Menzies	Name (10)	Mark Arends
Name (5)	Roderick Carter	Name (11)	Roland Stimson
Name (6)	Ana Garcia Diaz	Name (12)	Nicholas Morton

New Authorship

All authors must sign below agreeing to the changes in authorship. The authorship order must reflect the authorship order of the manuscript.

Name		Signature	Date
Name (1)	Neza Alfazema		
Name (2)	Marjorie Barrier		
Name (3)	Sophie Marion de Proce		
Name (4)	Robert Menzies		
Name (5)	Roderick Carter		
Name (6)	Kevin Stewart		
Name (7)	Ana Garcia Diaz		
Name (8)	Ben Moyon		
Name (9)	Zoe Webster		
Name (10)	Christopher Bellamy		
Name (11)	Mark Arends	[illegible]	25 FEB 2019
Name (12)	Roland Stimson		

Please print form and fax to 214-706-1565.

Change of Authorship Form

(Must be completed and signed by ALL authors)

Please check all that apply

✓ New author(s) have been added (in addition to this form, all new authors must complete the copyright transfer agreement and conflict of interest disclosure.

_____Change in order of authorship.

_____An author wishes to remove his/her name. An author's name may only be removed his/her own request and a letter signed by the author should accompany this form

Manuscript Number HYPE201812409

Manuscript Title Camk2n1 is a negative regulator of blood pressure, left ventricular mass, insulin sensitivity and promotes adiposity

Former Authorship

Please list ALL AUTHORS in the same order as the original submission. For more than 12, use an extra sheet.

Print Name		Print Name	
Name (1)	Neza Alfazema	Name (7)	Ben Moyon
Name (2)	Marjorie Barrier	Name (8)	Zoe Webster
Name (3)	Sophie Marion de Proce	Name (9)	Christopher Bellamy
Name (4)	Robert Menzies	Name (10)	Mark Arends
Name (5)	Roderick Carter	Name (11)	Roland Stimson
Name (6)	Ana Garcia Diaz	Name (12)	Nicholas Morton

New Authorship

All authors must sign below agreeing to the changes in authorship. The authorship order must reflect the authorship order of the manuscript.

Name		Signature	Date
Name (1)	Neza Alfazema		
Name (2)	Marjorie Barrier		
Name (3)	Sophie Marion de Proce		
Name (4)	Robert Menzies		
Name (5)	Roderick Carter		
Name (6)	Kevin Stewart		
Name (7)	Ana Garcia Diaz		
Name (8)	Ben Moyon		
Name (9)	Zoe Webster		
Name (10)	Christopher Bellamy		
Name (11)	Mark Arends		
Name (12)	Roland Stimson	[signature]	28/2/19

Please print form and fax to 214-706-1565.

Change of Authorship Form

(Must be completed and signed by ALL authors)

Please check all that apply

_✓_ New author(s) have been added (in addition to this form, all new authors must complete the copyright transfer agreement and conflict of interest disclosure.

_____ Change in order of authorship.

_____ An author wishes to remove his/her name. An author's name may only be removed his/her own request and a letter signed by the author should accompany this form

Manuscript Number HYPE201812409

Manuscript Title Camk2n1 is a negative regulator of blood pressure, left ventricular mass, insulin sensitivity and promotes adiposity

Former Authorship

Please list ALL AUTHORS in the same order as the original submission. For more than 12, use an extra sheet.

Print Name		Print Name	
Name (1)	(13) Timothy Aitman	Name (7)	
Name (2)	(14) Philip Coan	Name (8)	
Name (3)		Name (9)	
Name (4)		Name (10)	
Name (5)		Name (11)	
Name (6)		Name (12)	

New Authorship

All authors must sign below agreeing to the changes in authorship. The authorship order must reflect the authorship order of the manuscript.

Name		Signature	Date
Name (1)	(13) Nicholas Morton	[signature]	24/2/19
Name (2)	(14) Timothy Aitman		
Name (3)	(15) Philip Coan		
Name (4)			
Name (5)			
Name (6)			
Name (7)			
Name (8)			
Name (9)			
Name (10)			
Name (11)			
Name (12)			

Please print form and fax to 214-706-1565.

Change of Authorship Form

(Must be completed and signed by ALL authors)

Please check all that apply

__✓__ New author(s) have been added (in addition to this form, all new authors must complete the copyright transfer agreement and conflict of interest disclosure.

_____ Change in order of authorship.

_____ An author wishes to remove his/her name. An author's name may only be removed his/her own request and a letter signed by the author should accompany this form

Manuscript Number HYPE201812409

Manuscript Title Camk2n1 is a negative regulator of blood pressure, left ventricular mass, insulin sensitivity and promotes adiposity

Former Authorship

Please list ALL AUTHORS in the same order as the original submission. For more than 12, use an extra sheet.

**Print Name**		**Print Name**	
Name (1)	(13) Timothy Aitman	Name (7)	
Name (2)	(14) Philip Coan	Name (8)	
Name (3)		Name (9)	
Name (4)		Name (10)	
Name (5)		Name (11)	
Name (6)		Name (12)	

New Authorship

All authors must sign below agreeing to the changes in authorship. The authorship order must reflect the authorship order of the manuscript.

Name		Signature	Date
Name (1)	(13) Nicholas Morton		
Name (2)	(14) Timothy Aitman	Tim Aitman	26-02-2019
Name (3)	(15) Philip Coan		
Name (4)			
Name (5)			
Name (6)			
Name (7)			
Name (8)			
Name (9)			
Name (10)			
Name (11)			
Name (12)			

Please print form and fax to 214-706-1565.

Change of Authorship Form

(Must be completed and signed by ALL authors)

Please check all that apply

_✓_ New author(s) have been added (in addition to this form, all new authors must complete the copyright transfer agreement and conflict of interest disclosure.

_____ Change in order of authorship.

_____ An author wishes to remove his/her name. An author's name may only be removed his/her own request and a letter signed by the author should accompany this form

Manuscript Number HYPE201812409

Manuscript Title Camk2n1 is a negative regulator of blood pressure, left ventricular mass, insulin sensitivity and promotes adiposity

Former Authorship

Please list ALL AUTHORS in the same order as the original submission. For more than 12, use an extra sheet.

Print Name		Print Name	
Name (1)	(13) Timothy Aitman	Name (7)	
Name (2)	(14) Philip Coan	Name (8)	
Name (3)		Name (9)	
Name (4)		Name (10)	
Name (5)		Name (11)	
Name (6)		Name (12)	

New Authorship

All authors must sign below agreeing to the changes in authorship. The authorship order must reflect the authorship order of the manuscript.

	Name	Signature	Date
Name (1)	(13) Nicholas Morton		
Name (2)	(14) Timothy Aitman		
Name (3)	(15) Philip Coan	[signature]	26/02/19
Name (4)			
Name (5)			
Name (6)			
Name (7)			
Name (8)			
Name (9)			
Name (10)			
Name (11)			
Name (12)			

Please print form and fax to 214-706-1565.
